# Establishing Reference Ranges and Evaluating Clinical Factors of the Complete Blood Count in Neonates Admitted to a Neonatal Intensive Care Unit

**DOI:** 10.1111/ijlh.70014

**Published:** 2025-11-06

**Authors:** Emily Hyde, Jarob Saker, Stephen Kennedy, Mark Anthony, Manu Vatish

**Affiliations:** ^1^ Nuffield Department of Women's and Reproductive Health University of Oxford Oxford UK; ^2^ Medical Scientific Department, Sysmex Europe Norderstedt Germany; ^3^ Newborn Care Oxford University Hospitals NHS Trust Oxford UK

## Abstract

**Introduction:**

Accurate complete blood count (CBC) reference intervals are essential for neonatal care. However, existing reference intervals do not account for key clinical variables such as sex, postnatal age, gestational age at birth and corticosteroid exposure. This study aims to establish updated CBC reference intervals for neonates admitted to a Neonatal Intensive Care Unit (NICU) while evaluating the effects of these factors on the CBC.

**Methods:**

In this retrospective cohort study, all neonates admitted to the NICU at the John Radcliffe Hospital (Oxford, United Kingdom) between January 2022 and January 2023 were eligible for inclusion. Routine CBCs were included if there was no suspicion of infection or necrotising enterocolitis, no recent surgical interventions and no signs of clinical deterioration. The effects of sex, gestational age at birth and postnatal age were assessed for 48 parameters of the CBC using multivariate ANOVAs. Reference intervals were calculated at the 95% level.

**Results:**

Among 3490 CBC results from 587 neonates, 386 results from 196 neonates met inclusion criteria. Sex‐related differences were observed in nine parameters. Gestational and postnatal age both significantly influenced 34 parameters. Reference intervals were produced for all 48 CBC parameters, with histograms and boxplots illustrating variations by sex, postnatal age and gestational age. Secondary analyses highlighted the effects of corticosteroid exposure.

**Conclusions:**

We present reference intervals for 48 neonatal CBC parameters, highlighting the influence of sex, postnatal age, gestational age at birth and corticosteroid exposure. These findings improve the interpretation of neonatal CBCs and propose criteria for defining a sufficiently healthy neonatal population for diagnostic research.

## 1 Introduction

In the United Kingdom, approximately 1 in 10 newborns are admitted to Neonatal Intensive Care Units (NICUs) for specialised medical or surgical care due to prematurity, birth complications, congenital anomalies or suspected infection [1]. During admission, hospital‐acquired infections are common because of neonatal vulnerability and frequent invasive procedures. Identifying clinical deterioration early, particularly in cases of infection, is critical but requires reliable biomarkers that minimise the risk of iatrogenic anaemia.

The complete blood count (CBC) is widely used in neonatal care but remains underutilised as a diagnostic tool. Modern haematology analysers can measure numerous parameters beyond traditional white blood cell (WBC) counts, haemoglobin levels and platelet counts. These extended parameters, particularly those related to immune cell activation, may provide valuable diagnostic insights, yet their baseline values in neonates remain poorly defined, particularly in neonates admitted to the NICU who are clinically stable.

Previous reviews have established neonatal reference ranges for the more widely reported CBC parameters (see Christensen et al. review [2]), yet they often do not consider factors such as sex, gestational age at birth and postnatal age, nor do they include newer immune cell activation parameters.

In addition, the effect of corticosteroid exposure on neonatal CBC parameters is unknown, despite the widespread use of corticosteroids to treat chronic lung disease in preterm neonates [3]. The impacts of corticosteroids on the adult immune system and bone marrow function are well‐established [4], so it would be reasonable to determine if these effects also exist within neonates.

This study aimed to address these gaps by providing a comprehensive analysis of CBC results in neonates admitted to a NICU, who were recovering or growing but not acutely unwell, accounting for sex, gestational age at birth and corrected postnatal age. The effects of corticosteroid exposure were also evaluated.

## 2 Materials and Methods

In this retrospective cohort study, all neonates who had a CBC test while admitted to the NICU at the John Radcliffe Hospital (Oxford, UK) between 13 January 2022 and 13 January 2023 were eligible for inclusion. Ethics approval was obtained from the Oxford Research Ethics Committee (REC 08/H0606/139). The study followed an opt‐out consent process, whereby parents were informed through patient information leaflets, and consent was assumed unless explicitly declined. Parents could withdraw consent at any stage without affecting medical care. All data were anonymised before analysis.

CBC results measured as part of routine clinical care on a Sysmex (Kobe, Japan) XN‐2000 analyser in the haematology laboratory or XN‐450 in the NICU were retrospectively collected. Both analysers operate using fluorescence flow cytometry and impedance methods and were cross‐validated prior to study commencement. Results included both routinely reported parameters and parameters generated but not routinely reported, such as the white blood cell (WBC) differential, reticulocyte characterisation and extended inflammation parameters. Nucleated red blood cell (NRBC) counts from the XN‐450, which lack external validation, were manually cross‐validated, and corresponding WBC and lymphocyte counts were adjusted accordingly.

Matched clinical data were extracted from paper and electronic patient records. Data were summarised for each day of admission—where multiple CBC results were performed on the same day, the average was calculated.

CBC results were included only if there were no clinical or laboratory indicators suggesting infection or clinical deterioration. Exclusion criteria were: suspicion of maternal or neonatal infection at birth (if within the first 3 days of life); blood, respiratory or cerebrospinal fluid microbiology culture requested, regardless of the result (±5 days); surgical intervention or partial/full blood transfusion (+2 days); suspicion of necrotising enterocolitis (±2 days); viral PCR requested, regardless of the result (± two days); C‐reactive protein measurement > 10 mg/L (±1 day); lactate measurement > 4 mmol/L (±1 day); admission of antibiotics or corticosteroids on the same day; three or more signs of clinical deterioration on the same day (tachycardia, tachypnoea, step up in ventilation method, > 10% increase in oxygen requirement, > 1 unit increase in flow or mean airway pressure, temperature instability without therapeutic cooling or hypotension).

**FIGURE 1:**
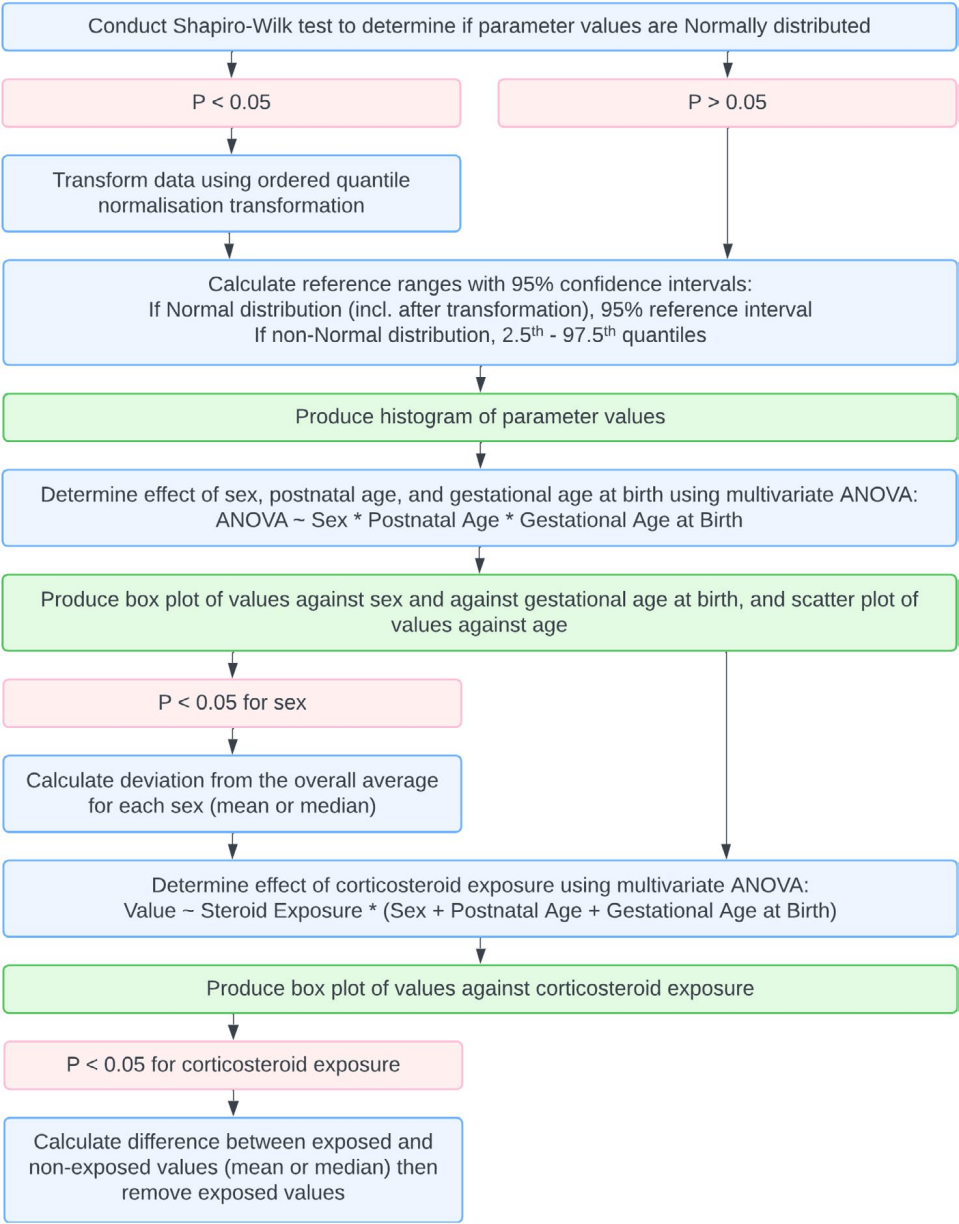
Flowchart of the statistical analysis steps performed in this study. Sequential ANOVAs were conducted to examine the effects of corticosteroid exposure, sex, postnatal age and gestational age at birth on each parameter of the neonatal complete blood count.

The statistical analysis workflow, detailed in Figure 1, follows Clinical and Laboratory Standards Institute (CLSI) guidelines for producing reference ranges (EP28 A3C) [5]. Forty‐eight parameters of the CBC were evaluated: 20 related to red blood cells (RBCs), 21 to WBCs and 7 to platelets.

Multivariate ANOVAs were conducted to assess the individual effects of and interactions between sex, gestational age at birth and postnatal age. An effect size (estimated using partial eta squared [PES]) greater than 0.06 was considered clinically relevant. Significant sex‐specific differences were calculated as deviations from the overall average.

Reference ranges were calculated parametrically (mean ± 1.96 × standard deviation) for normally distributed data (after transformation) and non‐parametrically (2.5% and 97.5% quantiles) otherwise. A minimum of 120 CBC results per parameter was required, per CLSI guidelines.

As a secondary analysis, the potential effects of corticosteroid exposure were evaluated separately using one‐way ANOVAs, and changes with exposure were calculated for any significantly affected parameters. This analysis was exploratory and not part of the core reference range generation. All analyses were conducted in RStudio (R version 4.3.0 (2023‐04‐21)) [6].

## 3 Results

During the study period, 3490 CBC results were collected from 587 neonates, with 386 results from 196 neonates meeting the inclusion criteria (Table 1). Ten neonates were excluded due to missing observational records. The distribution of males and females did not differ significantly by gestational age at birth, admission reason or discharge reason. Age distributions varied significantly across most factors, except discharge reason. While sex distributions were similar in corticosteroid‐exposed CBC results, these samples were obtained from older neonates (*p* < 0.001).

**TABLE 1:** Patient characteristics for neonates recruited from the Neonatal Intensive Care Unit at the John Radcliffe Hospital (Oxford, United Kingdom) to produce reference ranges for the complete blood count.

		All	Males	Females	Sex distribution *p*	Age at sampling (days)	Age distribution *p*
CBC results		330	176	154	0.23^a^	—	—
Neonates		184	98	86	0.38^a^	—	—
Age at sampling (days)		13 (7–36)	16 (9–48)	10 (4–28)	**< 0.001** ^b^	—	—
Gestational age at birth (weeks)		32.1 (27.6–37.3)	30.3 (27.4–36.3)	34.0 (27.6–37.6)	0.06^b^	—	—
Categories	Term (> 36 weeks)	53	22	31	0.11^a^	4 (1–9)	**< 0.001** ^c^
	Moderate preterm (32–36 weeks)	43	22	21		8 (2–13)	
	Very preterm (28–32 weeks)	33	22	11		23 (9–32)	
	Extreme preterm (< 28 weeks)	55	32	23		36 (13–64)	
Admission reason	Prematurity	95	54	41	0.57^a^	24 (9–46)	**< 0.001** ^c^
	Acute condition	44	23	21		6 (2–11)	
	Chronic condition	28	13	15		11 (2–23)	
	Planned surgery	10	7	3		63 (14–101)	
Discharge reason	Sent home	177	97	80	0.95^a^	14 (7–36)	0.17^c^
	Transferred	10	5	5		9 (2–14)	
	End‐of‐life care	2	1	1		36 (13–67)	
CBC results affected by steroids		56	36	20	0.17^a^	44 (29–69)	**< 0.001** ^c^

*Note:* Data are presented as counts or median (IQR). *p* values were calculated using ^a^chi‐squared goodness‐of‐fit test, ^b^Wilcoxon rank‐sum tests or ^c^Kruskal‐Wallis tests. Bold values indicates significant *p*‐value (< 0.05).

**FIGURE 2:**
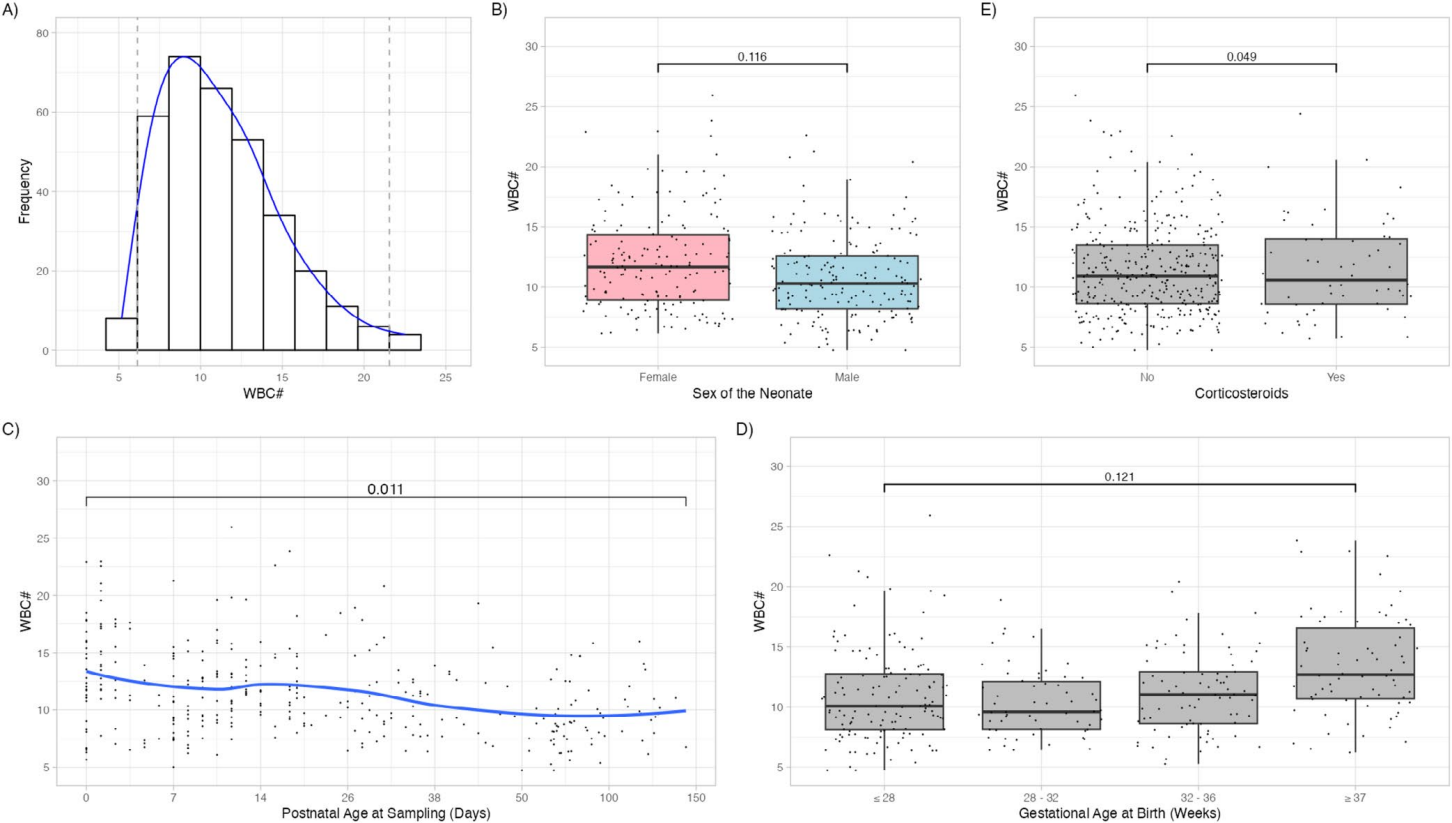
Analysis of the baseline profile of the neonatal white blood cell count (×10^9^/L). (A) Histogram with reference limits (grey dotted) and distribution curve (blue); (B) boxplot for sex; (C) scatterplot for age with a fitted curve (blue); (D) boxplot for gestational age at birth; and (E) boxplot for corticosteroid exposure.

The results of all ANOVA analyses are presented in Table 2, reference intervals and sex‐related deviations in Table 3, and a visual representation of these analyses for the WBC count in Figure 2. All S1: supplementary figures are available at https://4fsqon‐emily‐hyde.shinyapps.io/ReferenceRangeFigures/ [7].

**TABLE 2:** *p* value results of multivariate ANOVAs for the effects of corticosteroids, sex, postnatal age (days), and gestational age at birth (weeks) on parameters of the complete blood count in neonates admitted to the Newborn Care Unit.

	Parameter	Units	Sex		Age		GA		Sex and Age		Sex and GA		Age and GA		Sex, Age and GA		Corticosteroid exposure	
			*p*	PES	*p*	PES	*p*	PES	*p*	PES	*p*	PES	*p*	PES	*p*	PES	*p*	PES
Red blood cell parameters	Red blood cell count	×10^12^/L	0.01	0.020	0.07	0.010	< 0.001	0.197	0.40	0.002	0.03	0.015	< 0.001	0.234	< 0.001	0.064	0.59	0.001
	Haemoglobin concentration	g/L	0.09	0.009	< 0.001	0.107	< 0.001	0.163	0.16	0.006	0.009	0.021	< 0.001	0.212	< 0.001	0.074	0.64	0.001
	Haematocrit	%	0.14	0.007	< 0.001	0.076	< 0.001	0.159	0.13	0.007	0.01	0.020	< 0.001	0.239	< 0.001	0.069	0.73	0.000
	Mean cell volume	fL	0.23	0.004	< 0.001	0.319	0.22	0.005	0.02	0.016	0.27	0.004	0.02	0.016	0.74	0.000	0.002	0.024
	Mean cell haemoglobin	pg	0.25	0.004	< 0.001	0.405	0.24	0.004	0.06	0.011	0.36	0.003	0.12	0.008	0.35	0.003	0.006	0.020
	Mean cell haemoglobin concentration	g/L	0.79	0.000	< 0.001	0.192	0.56	0.001	0.82	0.000	0.58	0.001	0.30	0.003	0.34	0.003	0.19	0.005
	Red blood cell distribution width	fL	0.48	0.002	< 0.001	0.110	0.006	0.023	0.14	0.007	0.21	0.005	0.01	0.021	0.12	0.008	0.67	0.001
		%	0.30	0.003	0.29	0.004	0.003	0.028	0.64	0.001	0.21	0.005	0.005	0.024	0.08	0.009	0.19	0.004
	Nucleated red blood cell count	×10^9^/L	0.52	0.001	< 0.001	0.074	< 0.001	0.117	0.17	0.006	0.21	0.005	0.01	0.020	0.05	0.012	< 0.001	0.029
		%	0.35	0.003	< 0.001	0.065	< 0.001	0.127	0.16	0.006	0.20	0.005	0.04	0.014	0.12	0.008	< 0.001	0.036
	Reticulocyte count	×10^12^/L	0.86	0.000	0.96	0.000	0.94	0.000	0.84	0.000	0.29	0.003	< 0.001	0.061	0.002	0.029	0.003	0.023
		%	0.67	0.001	0.47	0.002	0.004	0.026	0.70	0.001	0.16	0.006	0.28	0.004	0.13	0.007	0.001	0.027
	Immature reticulocyte fraction	%	0.53	0.001	< 0.001	0.054	< 0.001	0.070	0.87	0.000	0.78	0.000	0.74	0.000	0.43	0.002	< 0.001	0.059
	Red blood cell haemoglobin equivalent	pg	0.06	0.011	< 0.001	0.300	< 0.001	0.044	0.15	0.007	0.31	0.003	0.05	0.011	0.23	0.004	0.94	0.000
	Reticulocyte haemoglobin equivalent	pg	0.02	0.016	0.04	0.014	< 0.001	0.205	0.50	0.001	0.50	0.001	0.04	0.014	< 0.001	0.044	< 0.001	0.131
	Haemoglobin equivalent difference	pg	0.57	0.001	< 0.001	0.128	< 0.001	0.085	0.86	0.000	0.57	0.001	0.30	0.003	0.002	0.030	< 0.001	0.158
	Hypochromic red blood cell count	%	< 0.001	0.039	0.18	0.006	< 0.001	0.255	1.00	0.000	0.83	0.000	< 0.001	0.036	0.58	0.001	< 0.001	0.086
	Hyperchromic red blood cell count	%	0.42	0.002	< 0.001	0.343	0.95	0.000	0.02	0.018	0.03	0.015	0.02	0.017	0.05	0.012	0.08	0.008
	Microcytic red blood cell count	%	0.02	0.018	< 0.001	0.111	< 0.001	0.111	0.86	0.000	0.19	0.005	0.006	0.024	0.70	0.001	0.21	0.004
	Macrocytic red blood cell count	%	0.80	0.000	< 0.001	0.206	0.43	0.002	0.002	0.029	0.04	0.014	< 0.001	0.039	0.36	0.003	0.02	0.014
White blood cell parameters	White blood cell count	×10^9^/L	0.12	0.008	0.01	0.020	0.12	0.007	0.61	0.001	0.50	0.001	0.12	0.008	0.14	0.007	0.05	0.010
	Neutrophil count	×10^9^/L	0.08	0.009	< 0.001	0.037	0.01	0.019	0.30	0.003	0.41	0.002	< 0.001	0.066	0.006	0.023	< 0.001	0.085
		%	0.09	0.009	0.001	0.032	< 0.001	0.034	0.11	0.008	0.42	0.002	< 0.001	0.126	< 0.001	0.037	< 0.001	0.161
	Lymphocyte count	×10^9^/L	0.82	0.000	0.08	0.010	0.04	0.013	0.71	0.000	0.96	0.000	< 0.001	0.046	0.34	0.003	< 0.001	0.068
		%	0.40	0.002	< 0.001	0.097	0.20	0.005	0.63	0.001	0.93	0.000	< 0.001	0.121	0.005	0.024	< 0.001	0.119
	Monocyte count	×10^9^/L	0.11	0.008	< 0.001	0.090	< 0.001	0.069	0.66	0.001	0.89	0.000	0.41	0.002	0.87	0.000	0.05	0.010
		%	0.31	0.003	< 0.001	0.061	< 0.001	0.137	0.52	0.001	0.98	0.000	0.008	0.022	0.09	0.009	0.18	0.005
	Eosinophil count	×10^9^/L	0.35	0.003	0.87	0.000	0.04	0.013	0.004	0.026	0.44	0.002	0.36	0.003	0.29	0.003	< 0.001	0.128
		%	0.14	0.007	0.20	0.005	0.007	0.023	0.004	0.025	0.24	0.004	0.15	0.006	0.16	0.006	< 0.001	0.155
	Basophil count	×10^9^/L	0.72	0.000	< 0.001	0.096	0.62	0.001	0.74	0.000	0.39	0.002	0.002	0.029	0.18	0.006	0.05	0.010
		%	0.10	0.009	< 0.001	0.102	0.60	0.001	0.84	0.000	0.12	0.008	< 0.001	0.040	0.29	0.003	0.002	0.025
	Immature granulocyte count	×10^9^/L	0.87	0.000	< 0.001	0.045	0.02	0.018	0.42	0.002	0.59	0.001	< 0.001	0.038	0.33	0.003	0.05	0.010
		%	0.76	0.000	< 0.001	0.039	0.07	0.010	0.17	0.006	0.19	0.005	< 0.001	0.034	0.41	0.002	0.16	0.005
	Neutrophil lymphocyte ratio	%	0.19	0.005	< 0.001	0.065	0.56	0.001	0.25	0.004	0.64	0.001	< 0.001	0.105	< 0.001	0.035	< 0.001	0.134
	Immature to total neutrophil ratio	%	0.22	0.005	0.01	0.020	0.44	0.002	0.03	0.015	0.05	0.012	0.25	0.004	0.01	0.019	0.02	0.015
	Neutrophil reactive intensity	FI	0.95	0.000	< 0.001	0.082	0.93	0.000	0.49	0.002	0.54	0.001	0.59	0.001	0.61	0.001	0.32	0.003
	Neutrophil granularity intensity	SI	0.22	0.005	0.008	0.022	< 0.001	0.177	0.15	0.006	0.34	0.003	0.08	0.009	0.40	0.002	0.002	0.025
	Reactive lymphocyte count	%L	0.03	0.015	< 0.001	0.037	< 0.001	0.078	0.52	0.001	0.34	0.003	0.51	0.001	0.76	0.000	< 0.001	0.063
	Antibody‐synthesising lymphocyte count	%L	0.31	0.003	0.89	0.000	< 0.001	0.042	0.53	0.001	0.37	0.003	0.61	0.001	0.05	0.012	0.03	0.013
	High fluorescing lymphocyte count	%L	0.24	0.004	0.74	0.000	< 0.001	0.060	0.24	0.004	0.27	0.004	0.03	0.014	0.12	0.008	0.30	0.003
	Reactive monocyte count	%M	0.04	0.013	0.17	0.006	0.01	0.019	0.46	0.002	0.96	0.000	0.10	0.008	0.09	0.009	0.25	0.004
Platelet parameters	Platelet count	×10^9^/L	0.008	0.021	0.25	0.004	0.42	0.002	0.29	0.003	0.96	0.000	< 0.001	0.077	0.61	0.001	< 0.001	0.047
	Platelet distribution width	%	0.50	0.002	0.09	0.010	< 0.001	0.053	0.39	0.003	0.17	0.007	0.12	0.009	0.05	0.014	0.09	0.009
	Mean platelet volume	fL	0.13	0.008	0.05	0.014	< 0.001	0.097	0.35	0.003	0.28	0.004	0.04	0.016	0.07	0.012	0.11	0.008
	Platelet large cell ratio	%	0.14	0.008	0.08	0.011	< 0.001	0.096	0.21	0.006	0.12	0.009	0.04	0.015	0.02	0.018	0.11	0.008
	Plateletcrit	%	0.32	0.004	0.42	0.002	< 0.001	0.057	0.27	0.004	0.71	0.001	< 0.001	0.071	0.54	0.001	0.003	0.026
	Immature platelet fraction	%	0.43	0.002	0.34	0.003	< 0.001	0.042	0.06	0.011	0.10	0.008	< 0.001	0.046	0.06	0.011	0.03	0.013
	Platelet to lymphocyte ratio	%	0.03	0.014	0.59	0.001	0.02	0.016	0.45	0.002	0.98	0.000	0.02	0.018	0.24	0.004	< 0.001	0.100

*Note:* Significant *p* values (*p* < 0.05) and effect sizes (PES > 0.06) are highlighted in orange.

Abbreviations: %L, percentage of lymphocytes; %M, percentage of monocytes; Age and GA, interaction between postnatal age and gestational age at birth (weeks); GA, gestational age at birth (weeks); *p*, ANOVA *p* value; PES, partial ETA squared (effect size); Sex and Age, interaction between sex and postnatal age; Sex and GA, interaction between sex and gestational age at birth (weeks); Sex, Age and GA, interaction between sex, postnatal age and gestational age at birth (weeks).

**TABLE 3:** Reference ranges for parameters of the complete blood count in neonates admitted to the Newborn Care Unit, and deviations from the average values with corticosteroid administration and for each sex.

	Parameter	Units	Lower	Average	Upper	Males	Females	Corticosteroids
Red blood cell parameters	Red blood cell count	×10^12^/L	2.43 (2.41 to 2.51)	3.74 (3.60 to 3.86)	6.03 (5.96 to 6.08)	−0.05	+0.10	
	Haemoglobin concentration	g/L	79 (78 to 81)	130 (122 to 139)	227 (224 to 229)			
	Haematocrit	%	21.9 (21.7 to 22.4)	35.6 (33.6 to 37.8)	60.7 (60.4 to 61.1)			
	Mean cell volume	fL	81.9 (81.6 to 82.8)	96.0 (94.8 to 96.9)	113.5 (113.0 to 113.9)			−7.7
	Mean cell haemoglobin	pg	28.8 (28.1 to 29.0)	34.9 (34.5 to 35.4)	42.9 (42.2 to 43.5)			−3.8
	Mean cell haemoglobin concentration	g/L	335 (333 to 336)	362 (361 to 364)	389 (388 to 391)			
	Red blood cell distribution width	fL	40.8 (40.6 to 41.8)	57.1 (56.2 to 57.9)	78.0 (76.6 to 82.6)			
		%	12.8 (12.5 to 13.3)	16.4 (16.2 to 16.8)	22.2 (21.8 to 22.6)			
	Nucleated red blood cell count	×10^9^/L	0.00 (0.00 to 0.00)	0.07 (0.06 to 0.10)	2.33 (1.64 to 2.54)			−0.04
		%	0.0 (0.0 to 0.0)	0.7 (0.5 to 1.0)	17.9 (13.0 to 21.7)			−0.4
	Reticulocyte count	×10^12^/L	0.027 (0.022 to 0.033)	0.129 (0.117 to 0.137)	0.363 (0.349 to 0.370)			−0.040
		%	0.8 (0.7 to 0.9)	3.7 (3.3 to 3.9)	9.8 (9.0 to 10.5)			−0.7
	Immature reticulocyte fraction	%	11.9 (10.7 to 13.1)	33.4 (32.2 to 34.5)	54.8 (53.6 to 56.0)			−7.4
	Red blood cell haemoglobin equivalent	pg	26.2 (25.8 to 26.7)	31.9 (31.6 to 32.3)	36.5 (36.5 to 36.7)			
	Reticulocyte haemoglobin equivalent	pg	22.2 (22.1 to 22.3)	30.2 (29.9 to 30.6)	37.2 (36.2 to 37.3)	−0.5	+0.8	+0.8
	Haemoglobin equivalent difference	pg	−8.2 (−8.8 to −8.1)	−1.4 (−1.8 to −1.2)	2.4 (2.1 to 2.5)			+2.9
	Hypochromic red blood cell count	%	0.3 (0.3 to 0.3)	1.5 (1.4 to 1.6)	6.2 (6.0 to 6.3)	+0.4	−0.3	−0.2
	Hyperchromic red blood cell count	%	0.1 (0.1 to 0.2)	1.4 (1.2 to 1.6)	11.4 (10.1 to 12.9)			
	Microcytic red blood cell count	%	1.1 (1.0 to 1.1)	2.9 (2.7 to 3.1)	10.2 (9.6 to 10.4)	+0.4	−0.6	
	Macrocytic red blood cell count	%	1.9 (1.8 to 2.0)	6.5 (6.0 to 7.0)	29.8 (28.6 to 31.5)			−2.5
White blood cell parameters	White blood cell count	×10^9^/L	6.14 (5.66 to 6.23)	10.82 (10.41 to 11.21)	21.55 (20.77 to 22.65)			+0.03
	Neutrophil count	×10^9^/L	1.11 (1.02 to 1.26)	3.79 (3.50 to 4.05)	13.84 (12.45 to 14.00)			+1.16
		%	15.7 (13.3 to 16.5)	36.2 (34.2 to 37.6)	66.1 (65.0 to 67.4)			+13.3
	Lymphocyte count	×10^9^/L	2.32 (2.21 to 2.39)	4.14 (4.04 to 4.24)	8.51 (8.24 to 8.64)			−0.71
		%	21.3 (20.8 to 22.0)	41.9 (40.7 to 43.0)	64.4 (62.7 to 66.4)			−11.2
	Monocyte count	×10^9^/L	0.55 (0.54 to 0.57)	1.56 (1.45 to 1.62)	3.47 (3.36 to 3.54)			+0.27
		%	5.8 (5.6 to 6.1)	14.3 (13.8 to 15.0)	26.0 (24.9 to 26.8)			
	Eosinophil count	×10^9^/L	0.06 (0.04 to 0.07)	0.39 (0.36 to 0.43)	1.80 (1.66 to 1.97)			−0.19
		%	0.5 (0.5 to 0.7)	3.9 (3.7 to 4.3)	16.1 (15.6 to 16.3)			−1.9
	Basophil count	×10^9^/L	0.02 (0.01 to 0.02)	0.10 (0.09 to 0.10)	0.24 (0.23 to 0.28)			−0.05
		%	0.2 (0.2 to 0.2)	0.7 (0.6 to 0.7)	1.8 (1.7 to 1.9)			−0.3
	Immature granulocyte count	×10^9^/L	0.02 (0.02 to 0.02)	0.10 (0.09 to 0.10)	1.57 (1.45 to 1.90)			−0.01
		%	0.3 (0.2 to 0.3)	1.1 (1.0 to 1.1)	9.0 (8.2 to 9.5)			
	Neutrophil lymphocyte ratio	%	0.25 (0.22 to 0.26)	1.03 (1.00 to 1.09)	3.16 (2.94 to 3.27)			+0.73
	Immature to total neutrophil ratio	%	0.01 (0.01 to 0.01)	0.03 (0.03 to 0.03)	0.17 (0.16 to 0.18)			−0.01
	Neutrophil reactive intensity	FI	36.2 (36.1 to 36.4)	42.6 (42.3 to 43.0)	53.0 (52.6 to 53.7)			
	Neutrophil granularity intensity	SI	137.9 (137.4 to 138.5)	147.7 (147.2 to 148.3)	157.6 (157.0 to 158.1)			+0.0
	Reactive lymphocyte count	%L	0.2 (0.2 to 0.3)	6.4 (5.7 to 7.7)	26.7 (25.6 to 27.4)	+0.0	+0.0	+9.4
	Antibody‐synthesising lymphocyte count	%L	0.0 (0.0 to 0.0)	0.3 (0.1 to 0.4)	1.1 (0.9 to 1.5)			+0.5
	High fluorescing lymphocyte count	%L	0.0 (0.0 to 0.0)	0.0 (0.0 to 0.0)	0.0 (0.0 to 0.0)			
	Reactive monocyte count	%M	0.0 (0.0 to 0.0)	0.2 (0.2 to 0.3)	4.6 (2.7 to 6.2)	+0.0	+0.0	
Platelet parameters	Platelet count	×10^9^/L	74 (70 to 86)	299 (286 to 312)	680 (662 to 705)	−10	+7	+122
	Platelet distribution width	%	9.7 (9.7 to 9.8)	13.0 (12.5 to 13.3)	23.4 (22.8 to 23.8)			
	Mean platelet volume	fL	9.5 (9.4 to 9.6)	11.4 (11.2 to 11.5)	13.5 (13.5 to 13.7)			
	Platelet large cell ratio	%	20.7 (20.5 to 21.0)	34.7 (34.1 to 35.5)	51.1 (50.9 to 53.0)			
	Plateletcrit	%	0.1 (0.1 to 0.1)	0.4 (0.3 to 0.4)	0.7 (0.7 to 0.8)			+0.2
	Immature platelet fraction	%	2.1 (2.0 to 2.4)	7.6 (7.2 to 8.0)	28.6 (27.7 to 29.0)			−0.5
	Platelet to lymphocyte ratio	%	0.017 (0.015 to 0.019)	0.068 (0.064 to 0.070)	0.171 (0.168 to 0.195)	−0.003	+0.002	+0.048

*Note:* Ranges are presented as mean (95% confidence interval) or median (95% quantiles). Positive changes from the reference with sex or corticosteroids are highlighted in green, while negative changes are highlighted in red.

Abbreviations: %L, percentage of lymphocytes; %M, percentage of monocytes.

Sex had a statistically significant effect on only eight parameters (*p* < 0.05), but none exceeded the threshold for clinical relevance (PES > 0.06; Figure 2B; Table 2). In contrast, postnatal age significantly influenced 31 parameters, with 20 showing clinically relevant effect sizes (Figure 2C). RBC and reticulocyte counts remained unaffected. Gestational age at birth significantly impacted 33 parameters, 16 of which demonstrated large effect sizes (Figure 2D), including increased nucleated RBC and monocyte counts in more preterm neonates.

In terms of interactions, six parameters were significantly influenced by the combined effect of sex and postnatal age, although none reached clinical relevance (Table 2). The interaction between sex and gestational age at birth significantly affected five parameters, all with small effect sizes. The interaction between postnatal age and gestational age at birth influenced 31 parameters, with 10 showing large effect sizes. The three‐way interaction between sex, postnatal age and gestational age at birth significantly affected 13 parameters, including RBC count, haemoglobin and haematocrit, all of which had large effect sizes.

Corticosteroid exposure significantly affected 31 parameters, with 12 surpassing the threshold for clinical relevance (PES > 0.06; Figure 2E; Table 2). Notably, lymphocyte counts decreased, while neutrophil counts and the platelet‐to‐lymphocyte ratio increased.

## 4 Discussion

In this study, we present reference ranges for 48 parameters of the CBC for neonates admitted to the NICU. Furthermore, we highlight the influence of sex, postnatal age, gestational age at birth and corticosteroid exposure on these parameters. Our findings highlight the dynamic nature of the neonatal CBC and emphasize the importance of accounting for contextual factors when interpreting results.

### 4.1 The Effect of Sex

Sexual dimorphism in the neonatal CBC has been described previously. Zierk et al. [8] reported sex differences in WBC counts during the first 60 days of life, with females presenting with higher values than males. Unlike Zierk et al., who included both inpatient and outpatient data from birth to 18 years of age, our study specifically examines neonates admitted to the NICU, offering a more focused perspective on this population. In the current study, no parameters showed clinically relevant differences between males and females, as shown by the low effect sizes.

### 4.2 The Effect of Postnatal Age

Age‐related changes in the neonatal CBC are well‐documented. RBC parameters, including counts, haemoglobin concentration and indices, typically decrease within the first 60 days of life, while platelet counts increase [8]. Similarly, WBC counts initially rise before declining [8]. Our findings align with this, although we observed more dynamic fluctuations in WBC counts during the first few weeks of life which were clinically not relevant (PES = 0.02). For nucleated RBCs and reticulocytes, our results also reflect known trends of age‐related decreases [9, 10], but visual analysis suggests a more nuanced pattern. These findings expand upon prior literature by characterising CBC parameters across the neonatal age spectrum.

### 4.3 The Effect of Gestational Age at Birth

The relationship between gestational age at birth and neonatal CBC parameters has been less thoroughly studied. Earlier preterm neonates exhibit higher nucleated RBC counts at birth, reflecting their developmental stage [10]. Our study confirms this trend and also identifies gestational age‐associated changes in monocyte counts, which have been previously described [11]. These findings provide further insight into the haematological profiles of preterm neonates and underscore the importance of adjusting reference ranges based on gestational age.

### 4.4 The Effect of Corticosteroid Exposure

Corticosteroids are commonly used in NICU settings to treat respiratory distress, particularly in preterm neonates [12]. While the effects of corticosteroids on the adult CBC are well‐documented [13], this study is the first to examine these effects in neonates. Consistent with adult studies, corticosteroids increased neutrophil counts and decreased lymphocyte counts. However, unlike in adults, corticosteroids did not affect overall RBC counts. Instead, reductions were observed in nucleated RBC and reticulocyte counts. These findings, while secondary to the core baseline evaluation, provide novel preliminary insights and warrant further investigation. To fully understand the effects of corticosteroid exposure, it would be necessary to establish specific reference intervals—this was not possible in the current study due to low sample numbers.

### 4.5 Overall Results

Despite its routine use, the CBC remains underutilised in the clinical management of neonates as only a few parameters are routinely reported. Historically, variability in the neonatal WBC count, compounded by limitations of early analysers to correctly identify nucleated RBCs from WBCs, led to scepticism about its diagnostic utility [14]. As such, the guidelines for diagnosing neonatal sepsis, published by the National Institute for Health and Care Excellence, do not recommend measuring the CBC [15].

Our findings challenge this perception. The calculated reference ranges were produced from a cohort of otherwise healthy neonates, such as preterm neonates or neonates admitted for observation after a traumatic birth, so deviations may have clinical significance. Furthermore, parameters of the CBC, such as neutrophil and lymphocyte activation markers, could serve as valuable indicators of neonatal deterioration, as shown in adult studies [16].

Although not a direct comparison, our findings echo those shown in the analysis of the cord blood baseline profile. For example, Sabnis et al. [17] observed no statistically significant differences between males and females in any parameters except mean cell haemoglobin content, while Angelo et al. [18] observed sex‐related differences only in the red cell distribution width. In the current study, we found six parameters that differed with sex, although none of these were clinically relevant.

### 4.6 Strengths and Limitations

This study provides robust, NICU‐specific reference ranges for 48 CBC parameters, derived using rigorous methodology. Although the data were obtained from a single centre, the conclusion that these factors should be considered when evaluating the CBC is generalizable and broadly applicable to NICUs globally. Furthermore, our approach is reproducible and can be applied in other centres. A key strength is the careful consideration of confounding factors, including corticosteroid exposure, sex, corrected postnatal age and gestational age at birth. However, there may be other factors that require evaluation in future research, for example, the effects of IUGR.

Another notable strength of our study is the careful selection of a subpopulation of NICU‐admitted neonates that were considered otherwise ‘healthy’ (Table 1). This subpopulation forms the basis for producing reliable reference ranges. Current studies on diagnostic tests for unwell neonates, such as those by Worku et al. [19] and Yin et al. [20], often rely on insufficient control definitions, including neonates with clinical suspicion of sepsis and negative blood cultures. Given the high false‐negative rate of blood cultures in this population, such control groups are likely to include infected neonates, undermining their validity. By contrast, our study employed stringent exclusion criteria (Table 1), setting a benchmark for future research.

One limitation of this study was the uncertainty surrounding the timings of results. For example, the exact timing of corticosteroid administration relative to CBC results was not known. To mitigate the possible longer‐lasting effects of corticosteroids, results taken within 5 days of a final dose of corticosteroids (*n* = 12) or from the first 5 days of life if the mother received antenatal corticosteroids (*n* = 10) were excluded. Longitudinal analysis of the effects of corticosteroid administration was not possible due to low sample numbers. Similarly, the source of individual blood samples (e.g., venous or heel prick) was not available.

The age at sampling varied across admission reasons, reflecting different NICU populations rather than longitudinal changes within individuals. The reference ranges presented here broadly apply to all neonates admitted to NICUs, although more specific reference ranges for individual subsets (e.g., age‐related patterns in term neonates) would require a larger sample of results and thus should be the focus of future research.

## 5 Conclusion

This study proposes reference ranges for 48 CBC parameters in neonates and underscores the importance of considering sex, postnatal age, gestational age at birth and corticosteroid exposure in their interpretation. We also define robust criteria that are sufficient to identify a ‘healthy’ subpopulation that serves as a benchmark for investigating diagnostic tests in unwell neonates admitted to the NICU. These findings contribute to a more nuanced understanding of neonatal haematology and provide a foundation for improving diagnostic tools and clinical care in the NICU.

## Author Contributions

Emily Hyde contributed to conceptualising the study and designing the methodology, conducting data collection, performing the statistical analyses, interpreting the study results and drafting the manuscript. Jarob Saker contributed to conceptualising the study and designing the methodology, interpreting the study results, supervising the study, providing feedback on data analysis and manuscript revisions and acquiring funding to support the research. Stephen Kennedy contributed to designing the methodology, supervising the study and providing feedback on manuscript revisions. Mark Anthony contributed to conceptualising the study and designing the methodology, conducting data collection and overseeing the acquisition of clinical data from patient records, interpreting the study results, supervising the study and providing feedback on data analysis and manuscript revisions. Manu Vatish contributed to conceptualising the study and designing the methodology, interpreting the study results, supervising the study, providing feedback on data analysis and manuscript revisions and acquiring funding to support the research. All authors have approved the final manuscript as submitted and agree to be accountable for all aspects of the work.

## Ethics Statement

This study protocol was reviewed and approved by the Oxford Research Ethics Committee, approval number [REC 08/H0606/139].

## Consent

An opt‐out informed consent protocol was used for the use of participant data for research purposes. This consent procedure was reviewed and approved by the Oxford Research Ethics Committee, approval number [08/H0606/139/AM33], date of decision [17 November 2021].

## Conflicts of Interest

Jarob Saker is a permanent employee of Sysmex Europe GmbH. Sysmex provided a haematology analyser and free reagents for the duration of the project.

## Supporting information


**Data S1:** ijlh70014‐sup‐0001‐supinfo.pdf.

## Data Availability

The data that support the findings of this study are available on request from the corresponding author. The data are not publicly available due to privacy or ethical restrictions.
