# Supplementary material for: Establishing Reference Ranges and Evaluating Clinical Factors of the Complete Blood Count in Neonates Admitted to a Neonatal Intensive Care Unit
Source: Int J Lab Hematol. 2025 Nov 6;48(1):81–92. doi: 10.1111/ijlh.70014 (PMC12809373; doi:10.1111/ijlh.70014)
Supplement: Supplementary file 1 — Data S1: ijlh70014‐sup‐0001‐supinfo.pdf. [file IJLH-48-81-s001.pdf]

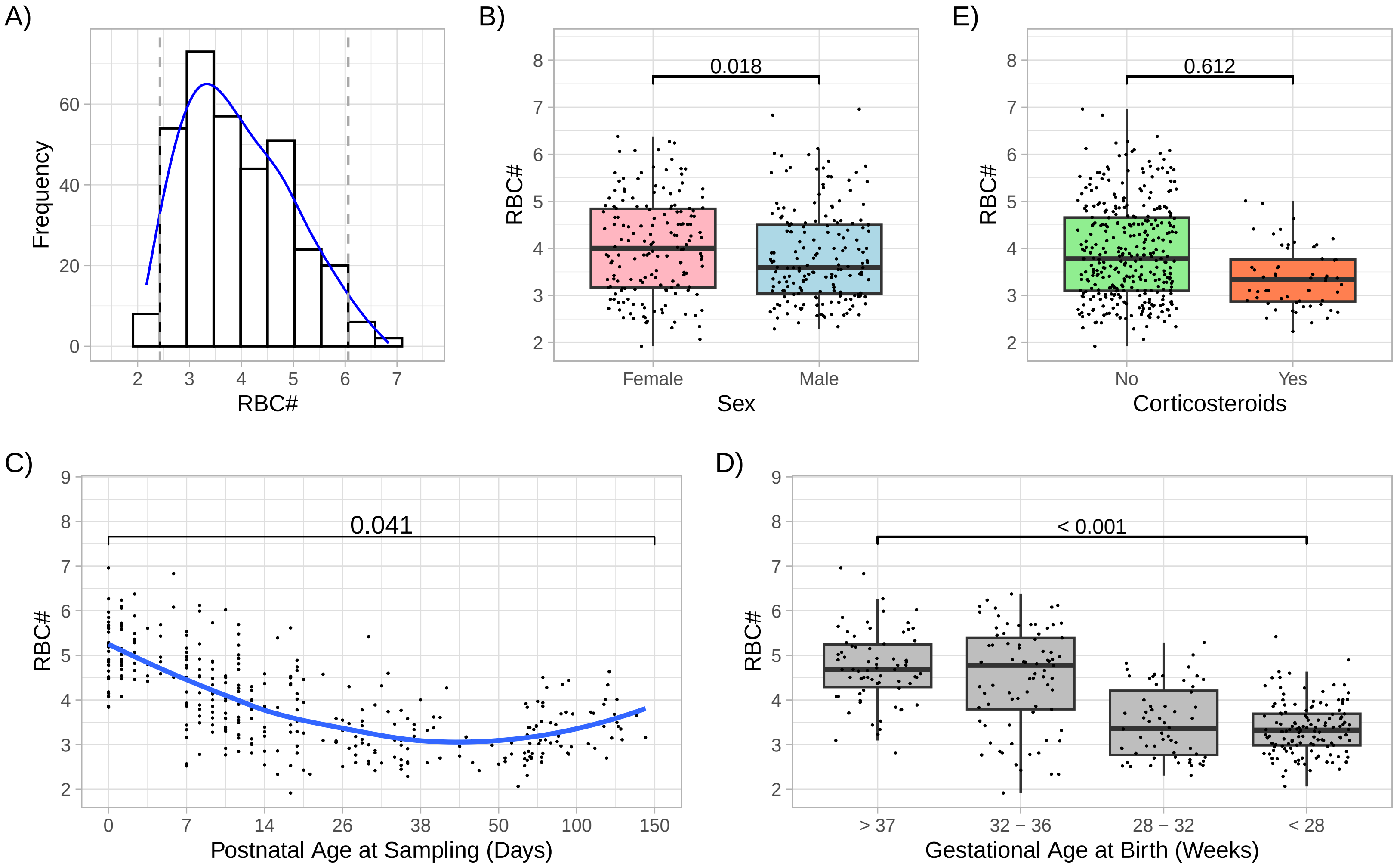

Figure S1: Evaluation of the baseline profile for red blood cell count (x10^12/L) in hospitalised but clinically well neonates. (A) Histogram with reference ranges (grey dotted lines) and a distribution curve (blue line). (B) Box plot of sex. (C) Scatter plot of postnatal age at sampling with a fitted curve (blue line). (D) Box plot of gestational age at birth. (E) Box plot of corticosteroid exposure. Corticosteroid exposed values were removed in plots A to D.

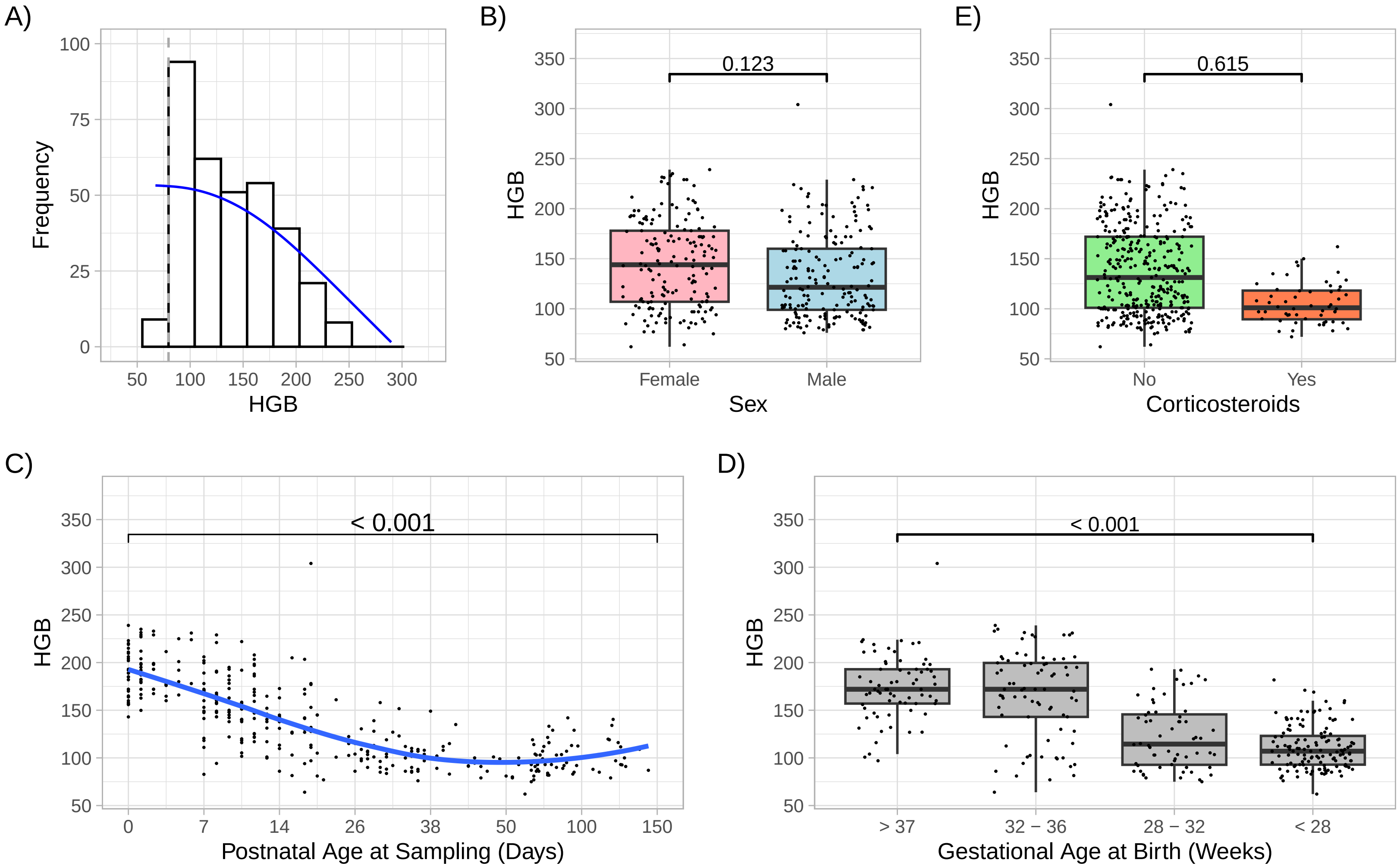

Figure S2: Evaluation of the baseline profile for haemoglobin concentration (g/L) in hospitalised but clinically well neonates. (A) Histogram with reference ranges (grey dotted lines) and a distribution curve (blue line). (B) Box plot of sex. (C) Scatter plot of postnatal age at sampling with a fitted curve (blue line). (D) Box plot of gestational age at birth. (E) Box plot of corticosteroid exposure. Corticosteroid exposed values were removed in plots A to D.

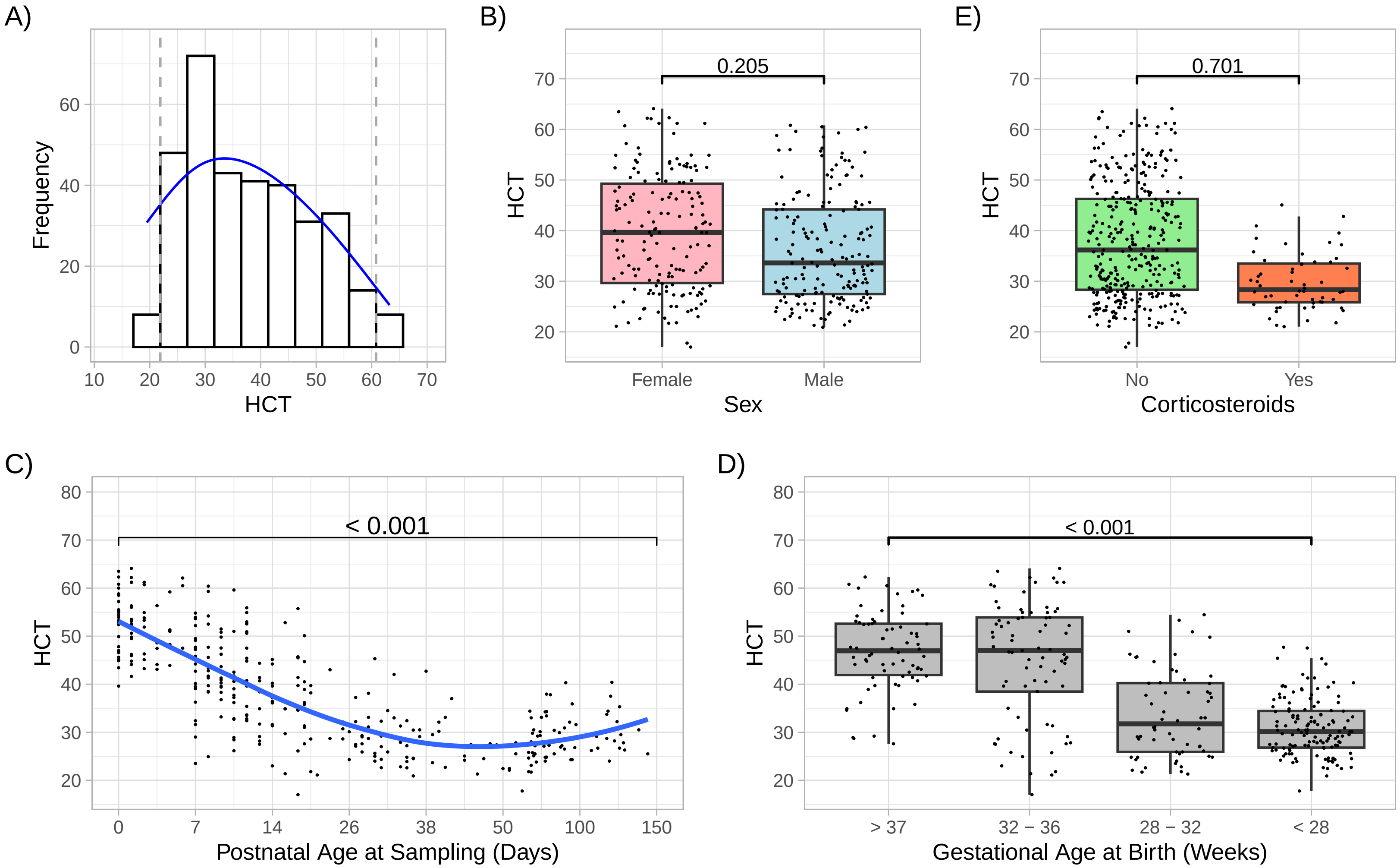

Figure S3: Evaluation of the baseline profile for haematocrit (%) in hospitalised but clinically well neonates. (A) Histogram with reference ranges (grey dotted lines) and a distribution curve (blue line). (B) Box plot of sex. (C) Scatter plot of postnatal age at sampling with a fitted curve (blue line). (D) Box plot of gestational age at birth. (E) Box plot of corticosteroid exposure. Corticosteroid exposed values were removed in plots A to D.

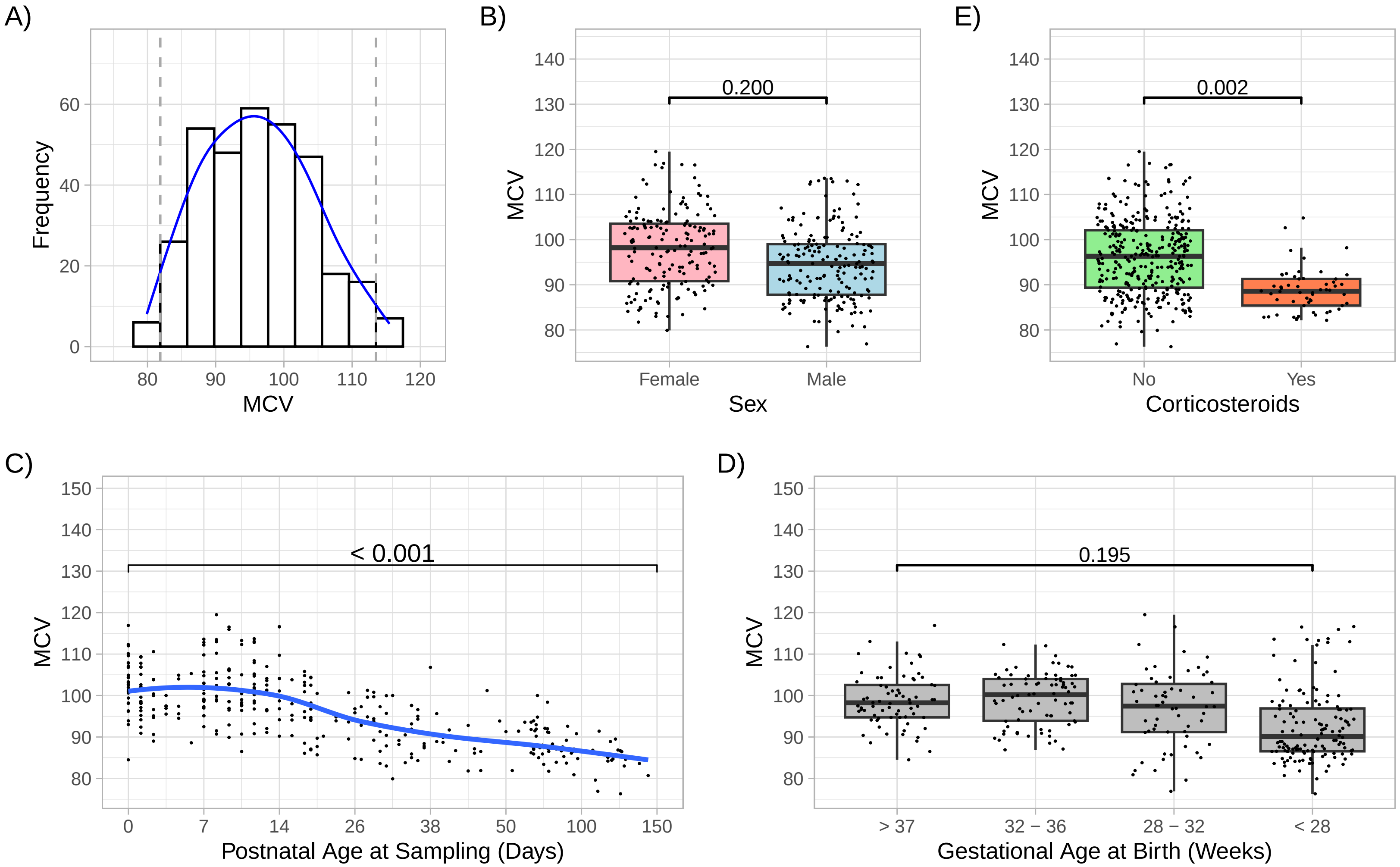

Figure S4: Evaluation of the baseline profile for mean cell volume (fL) in hospitalised but clinically well neonates. (A) Histogram with reference ranges (grey dotted lines) and a distribution curve (blue line). (B) Box plot of sex. (C) Scatter plot of postnatal age at sampling with a fitted curve (blue line). (D) Box plot of gestational age at birth. (E) Box plot of corticosteroid exposure. Corticosteroid exposed values were removed in plots A to D.

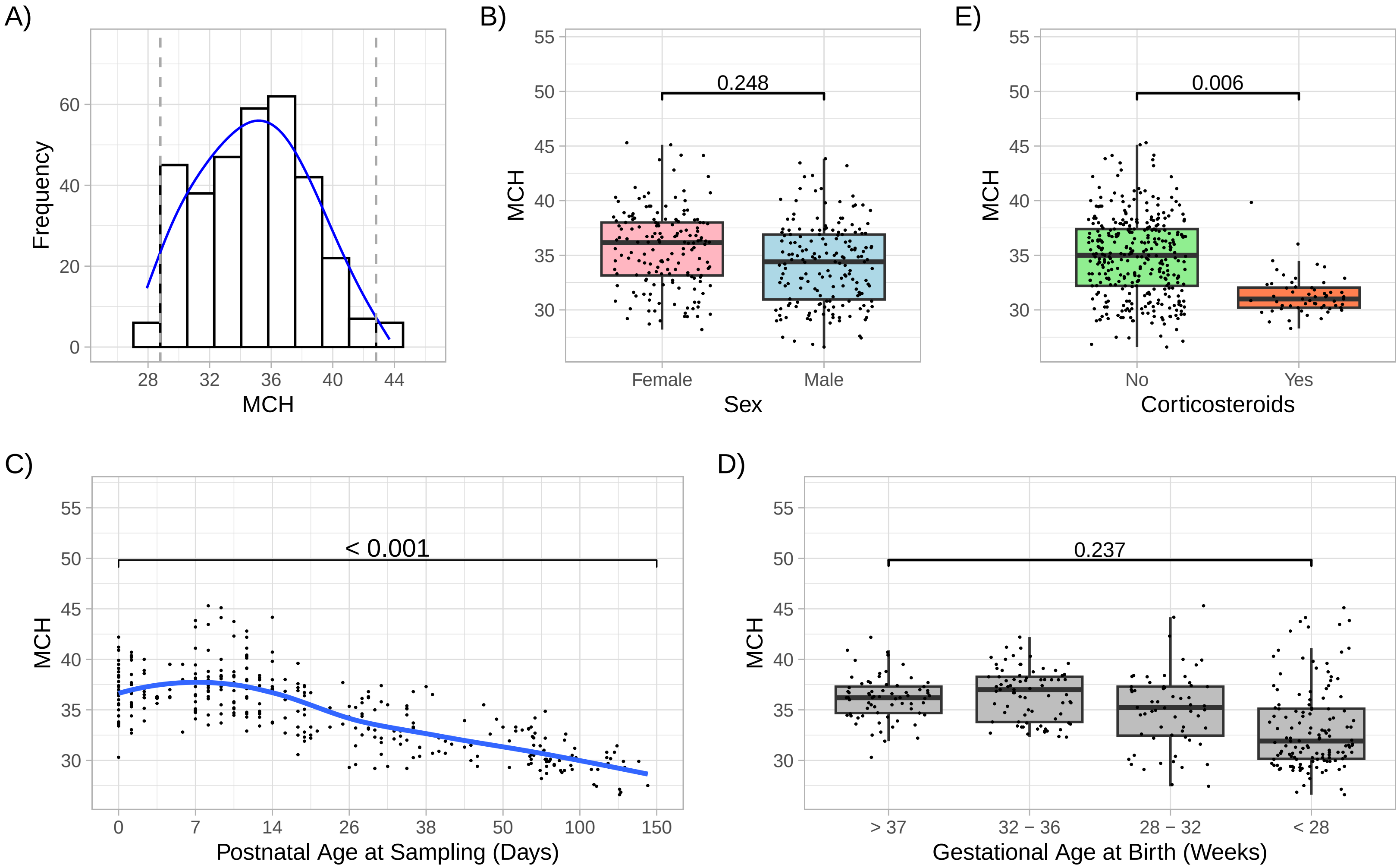

Figure S5: Evaluation of the baseline profile for mean cell haemoglobin (pg) in hospitalised but clinically well neonates. (A) Histogram with reference ranges (grey dotted lines) and a distribution curve (blue line). (B) Box plot of sex. (C) Scatter plot of postnatal age at sampling with a fitted curve (blue line). (D) Box plot of gestational age at birth. (E) Box plot of corticosteroid exposure. Corticosteroid exposed values were removed in plots A to D.

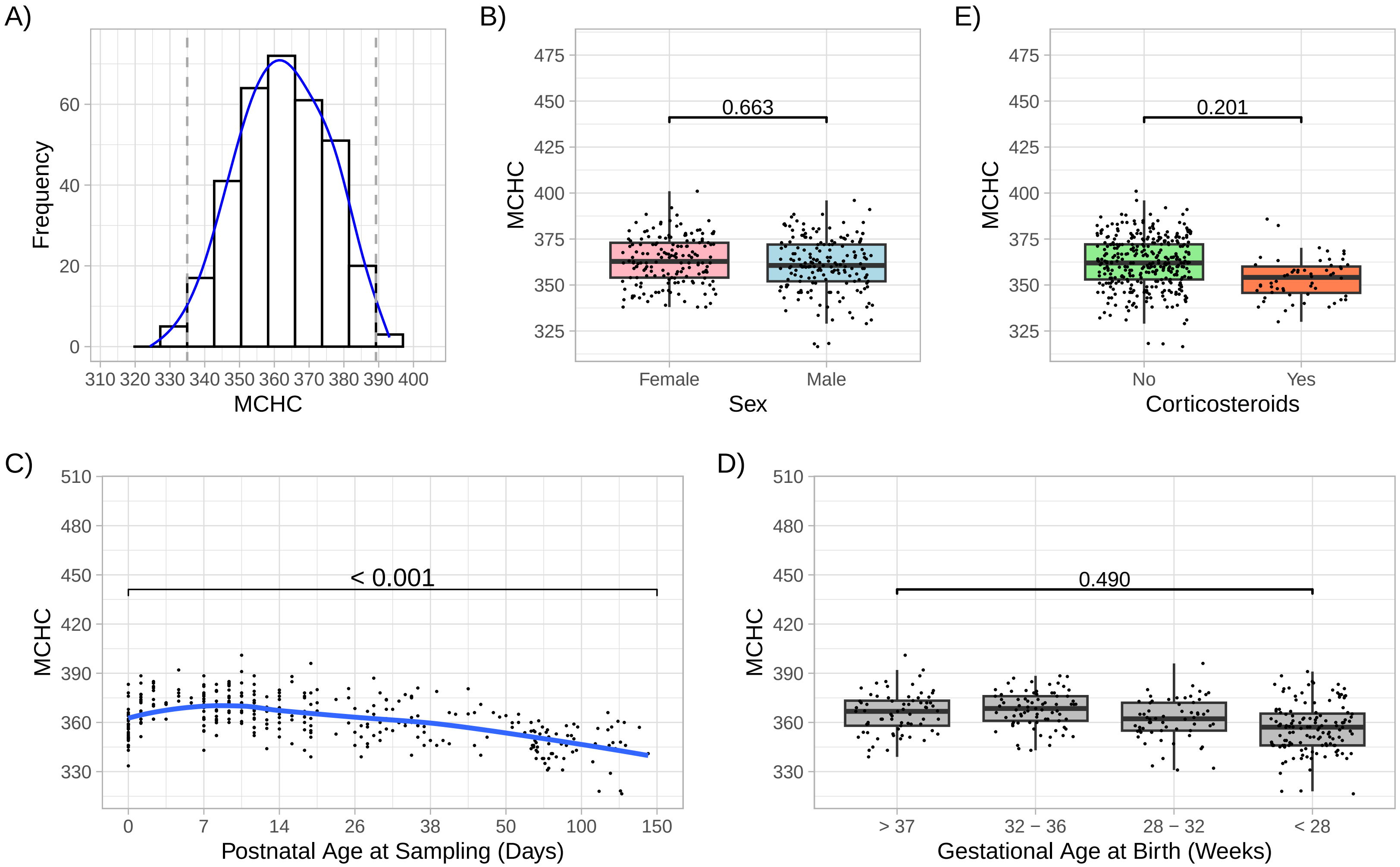

Figure S6: Evaluation of the baseline profile for mean cell haemoglobin concentration (g/L) in hospitalised but clinically well neonates. (A) Histogram with reference ranges (grey dotted lines) and a distribution curve (blue line). (B) Box plot of sex. (C) Scatter plot of postnatal age at sampling with a fitted curve (blue line). (D) Box plot of gestational age at birth. (E) Box plot of corticosteroid exposure. Corticosteroid exposed values were removed in plots A to D.

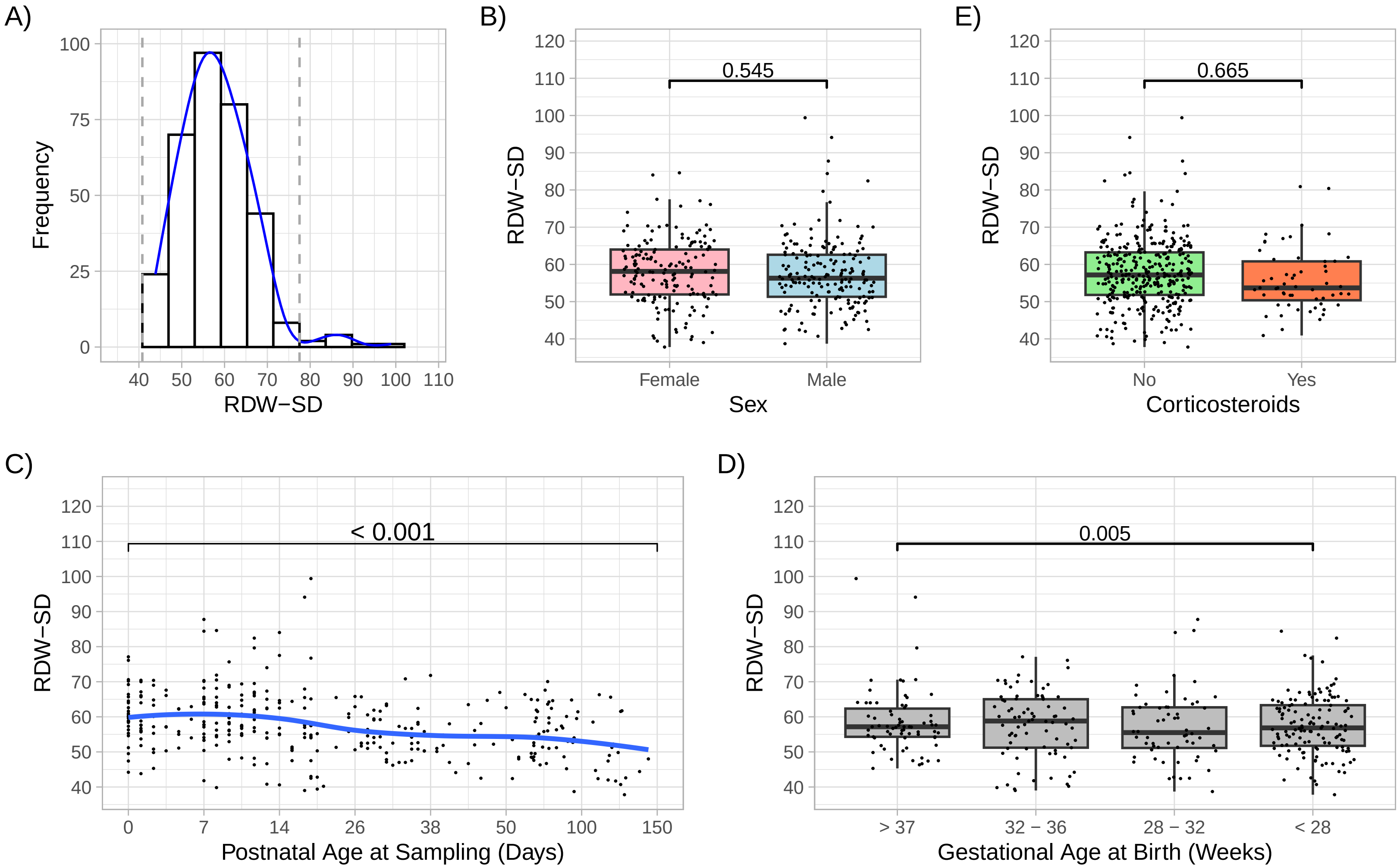

Figure S7: Evaluation of the baseline profile for red blood cell distribution width (fL) in hospitalised but clinically well neonates. (A) Histogram with reference ranges (grey dotted lines) and a distribution curve (blue line). (B) Box plot of sex. (C) Scatter plot of postnatal age at sampling with a fitted curve (blue line). (D) Box plot of gestational age at birth. (E) Box plot of corticosteroid exposure. Corticosteroid exposed values were removed in plots A to D.

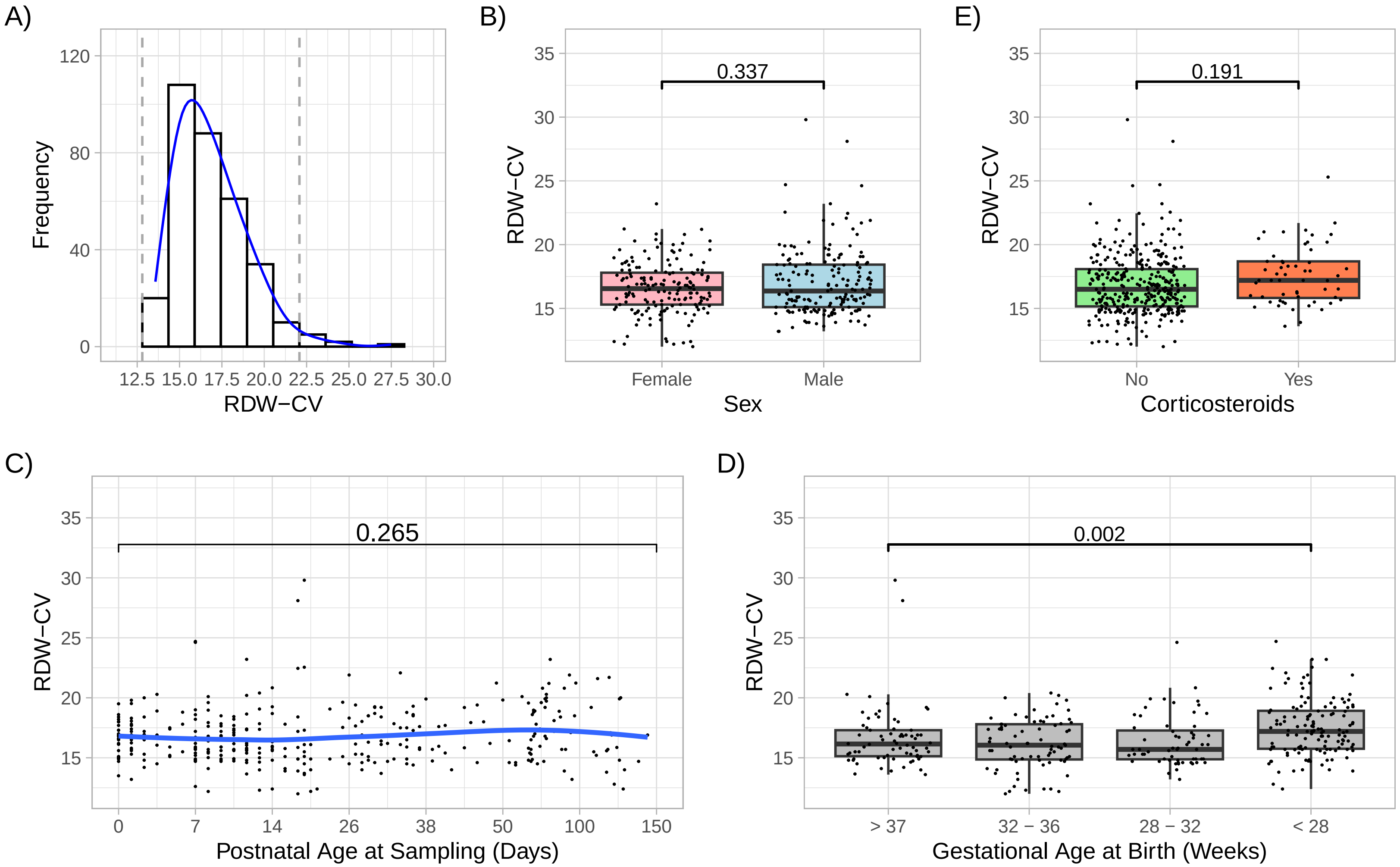

Figure S8: Evaluation of the baseline profile for red blood cell distribution width (%) in hospitalised but clinically well neonates. (A) Histogram with reference ranges (grey dotted lines) and a distribution curve (blue line). (B) Box plot of sex. (C) Scatter plot of postnatal age at sampling with a fitted curve (blue line). (D) Box plot of gestational age at birth. (E) Box plot of corticosteroid exposure. Corticosteroid exposed values were removed in plots A to D.

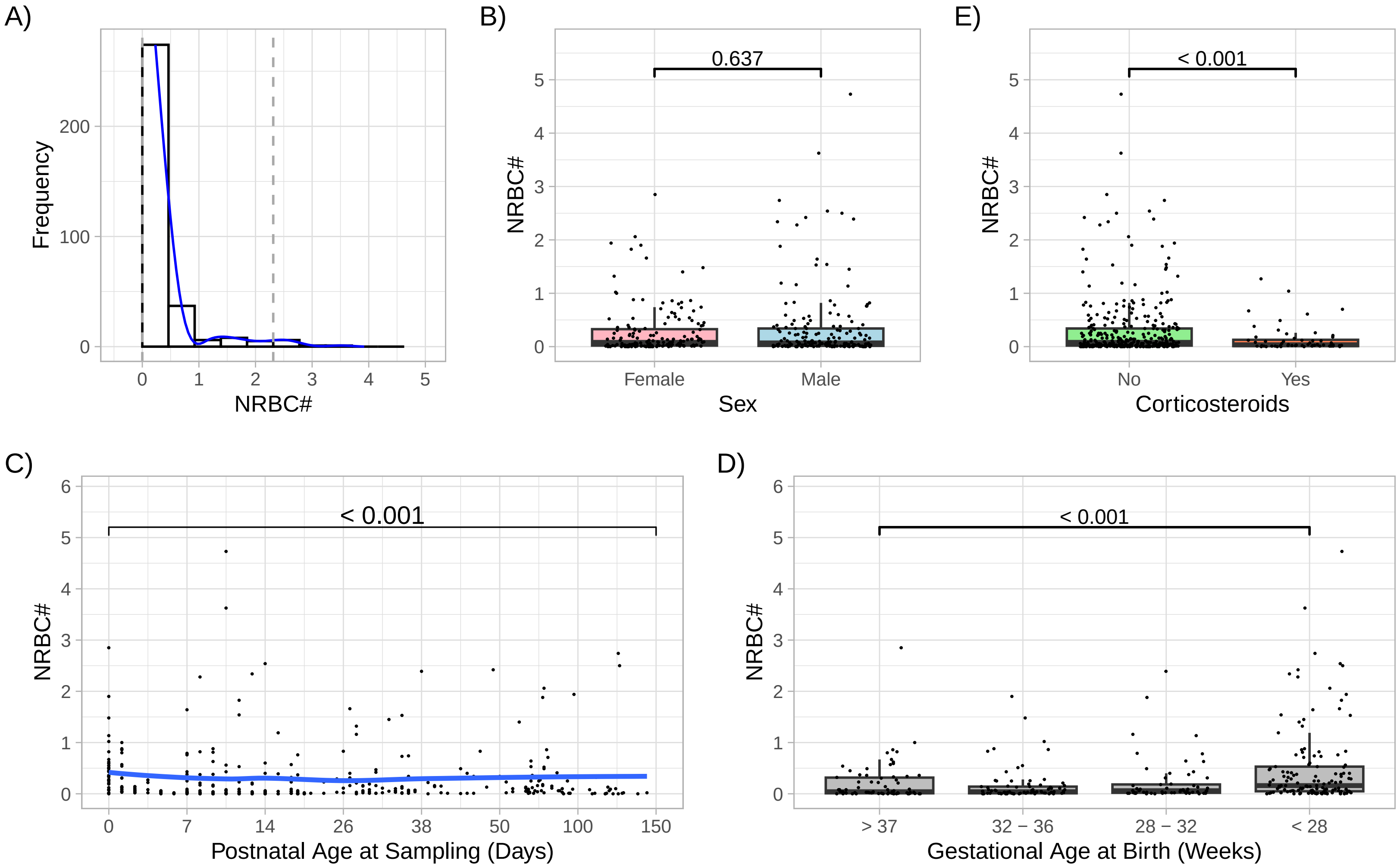

Figure S9: Evaluation of the baseline profile for nucleated red blood cell count (x10^9/L) in hospitalised but clinically well neonates. (A) Histogram with reference ranges (grey dotted lines) and a distribution curve (blue line). (B) Box plot of sex. (C) Scatter plot of postnatal age at sampling with a fitted curve (blue line). (D) Box plot of gestational age at birth. (E) Box plot of corticosteroid exposure. Corticosteroid exposed values were removed in plots A to D.

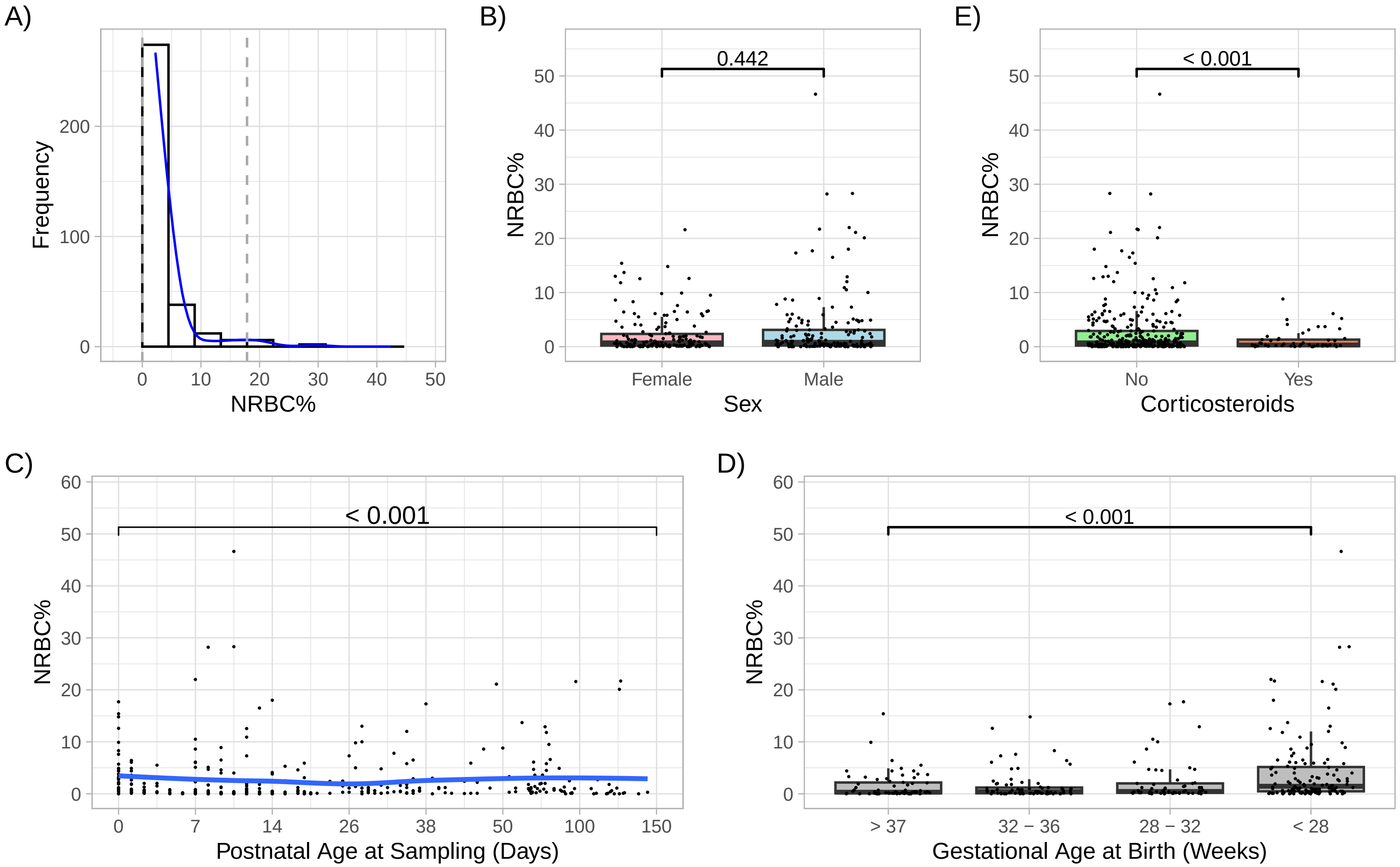

Figure S10: Evaluation of the baseline profile for nucleated red blood cell count (%) in hospitalised but clinically well neonates. (A) Histogram with reference ranges (grey dotted lines) and a distribution curve (blue line). (B) Box plot of sex. (C) Scatter plot of postnatal age at sampling with a fitted curve (blue line). (D) Box plot of gestational age at birth. (E) Box plot of corticosteroid exposure. Corticosteroid exposed values were removed in plots A to D.

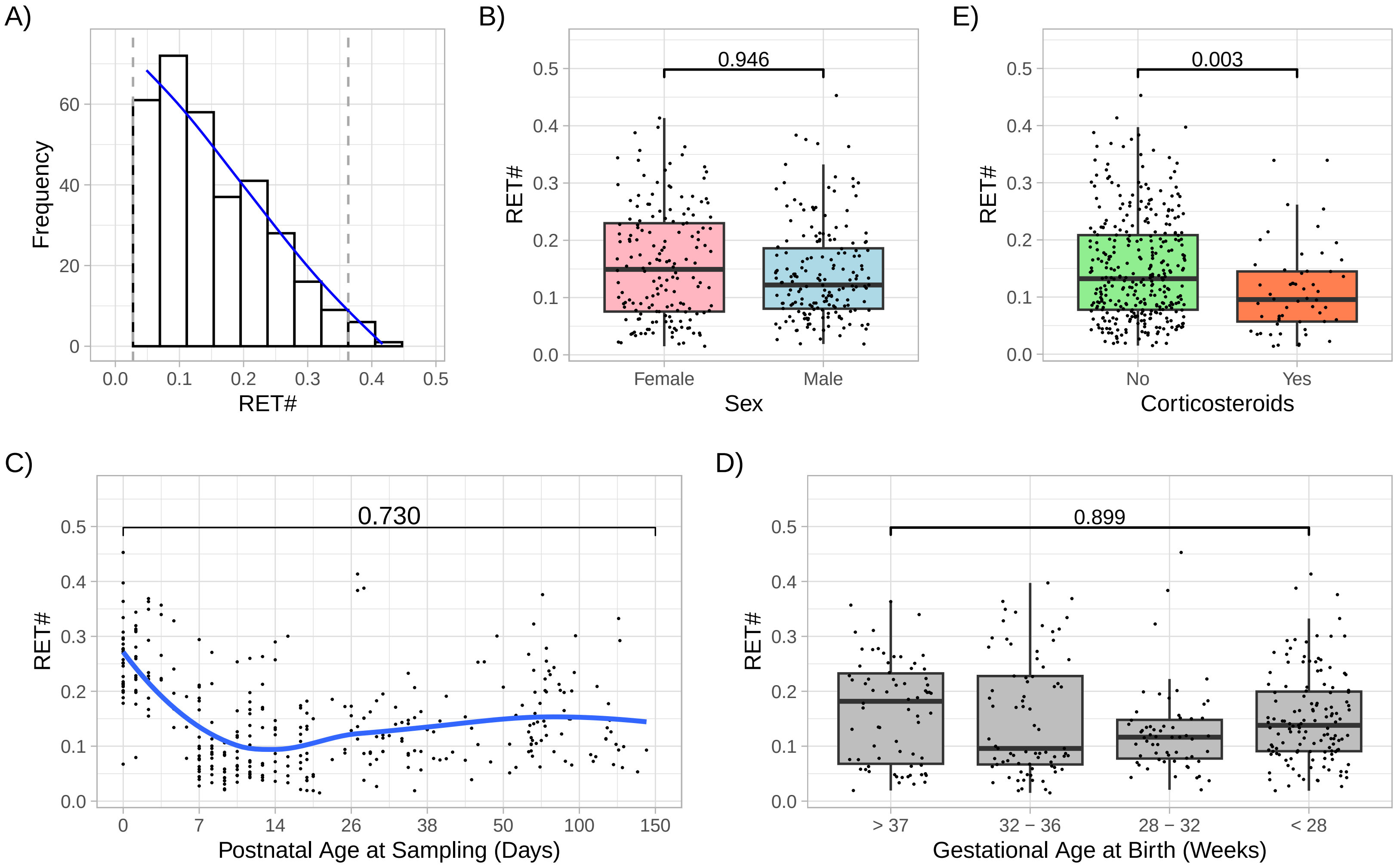

Figure S11: Evaluation of the baseline profile for reticulocyte count (x10^12/L) in hospitalised but clinically well neonates. (A) Histogram with reference ranges (grey dotted lines) and a distribution curve (blue line). (B) Box plot of sex. (C) Scatter plot of postnatal age at sampling with a fitted curve (blue line). (D) Box plot of gestational age at birth. (E) Box plot of corticosteroid exposure. Corticosteroid exposed values were removed in plots A to D.

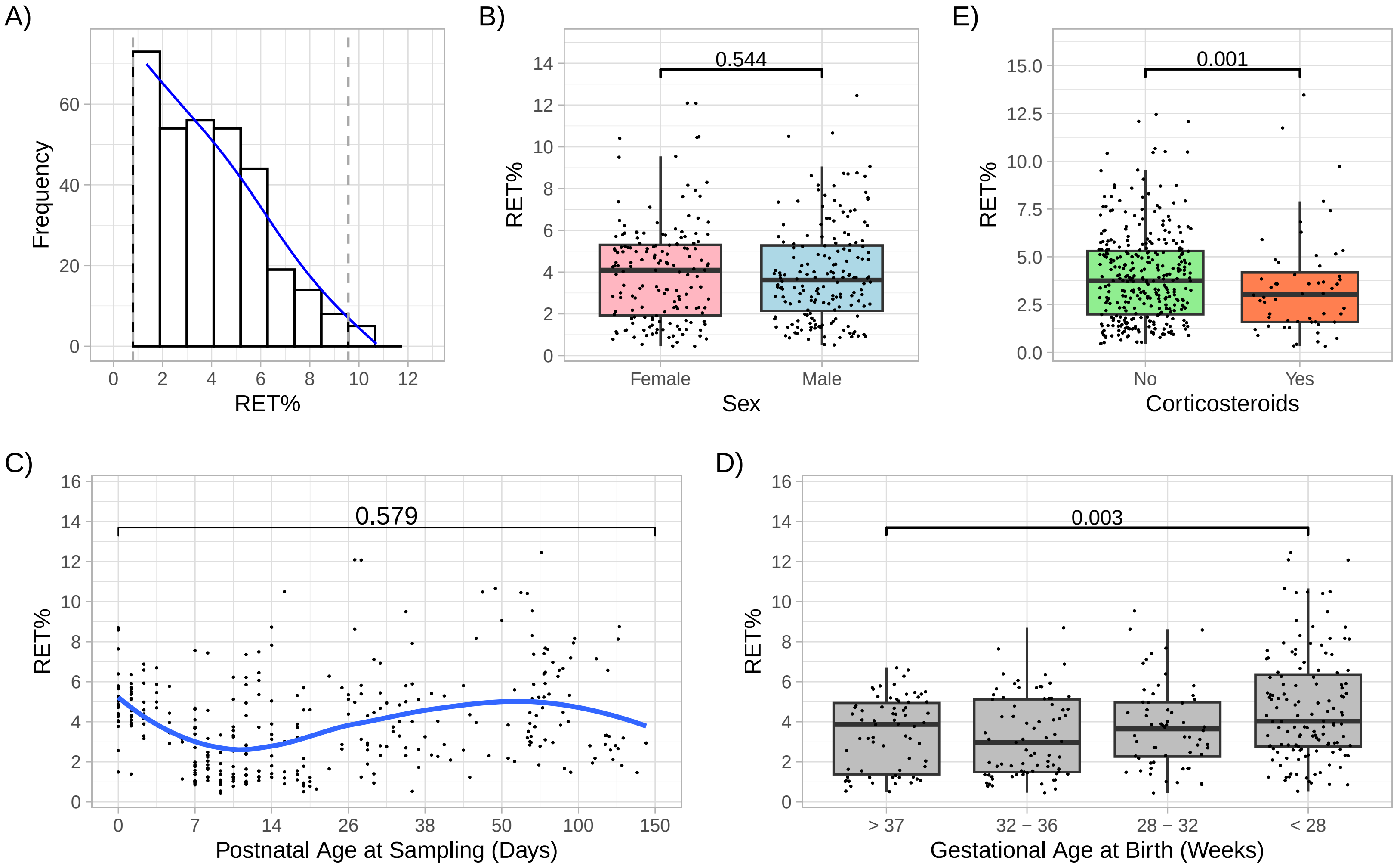

Figure S12: Evaluation of the baseline profile for reticulocyte count (%) in hospitalised but clinically well neonates. (A) Histogram with reference ranges (grey dotted lines) and a distribution curve (blue line). (B) Box plot of sex. (C) Scatter plot of postnatal age at sampling with a fitted curve (blue line). (D) Box plot of gestational age at birth. (E) Box plot of corticosteroid exposure. Corticosteroid exposed values were removed in plots A to D.

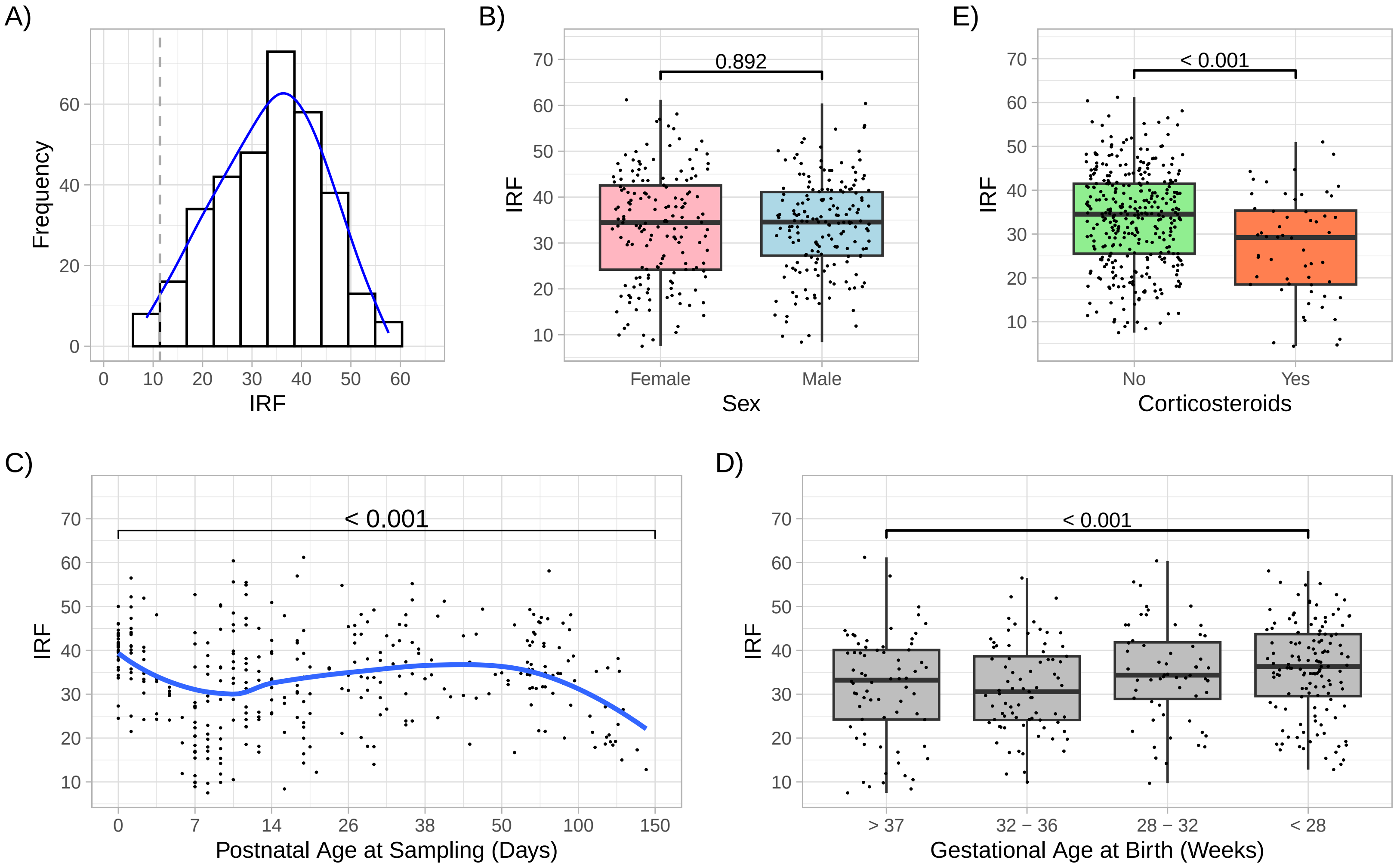

Figure S13: Evaluation of the baseline profile for immature reticulocyte fraction (%) in hospitalised but clinically well neonates. (A) Histogram with reference ranges (grey dotted lines) and a distribution curve (blue line). (B) Box plot of sex. (C) Scatter plot of postnatal age at sampling with a fitted curve (blue line). (D) Box plot of gestational age at birth. (E) Box plot of corticosteroid exposure. Corticosteroid exposed values were removed in plots A to D.

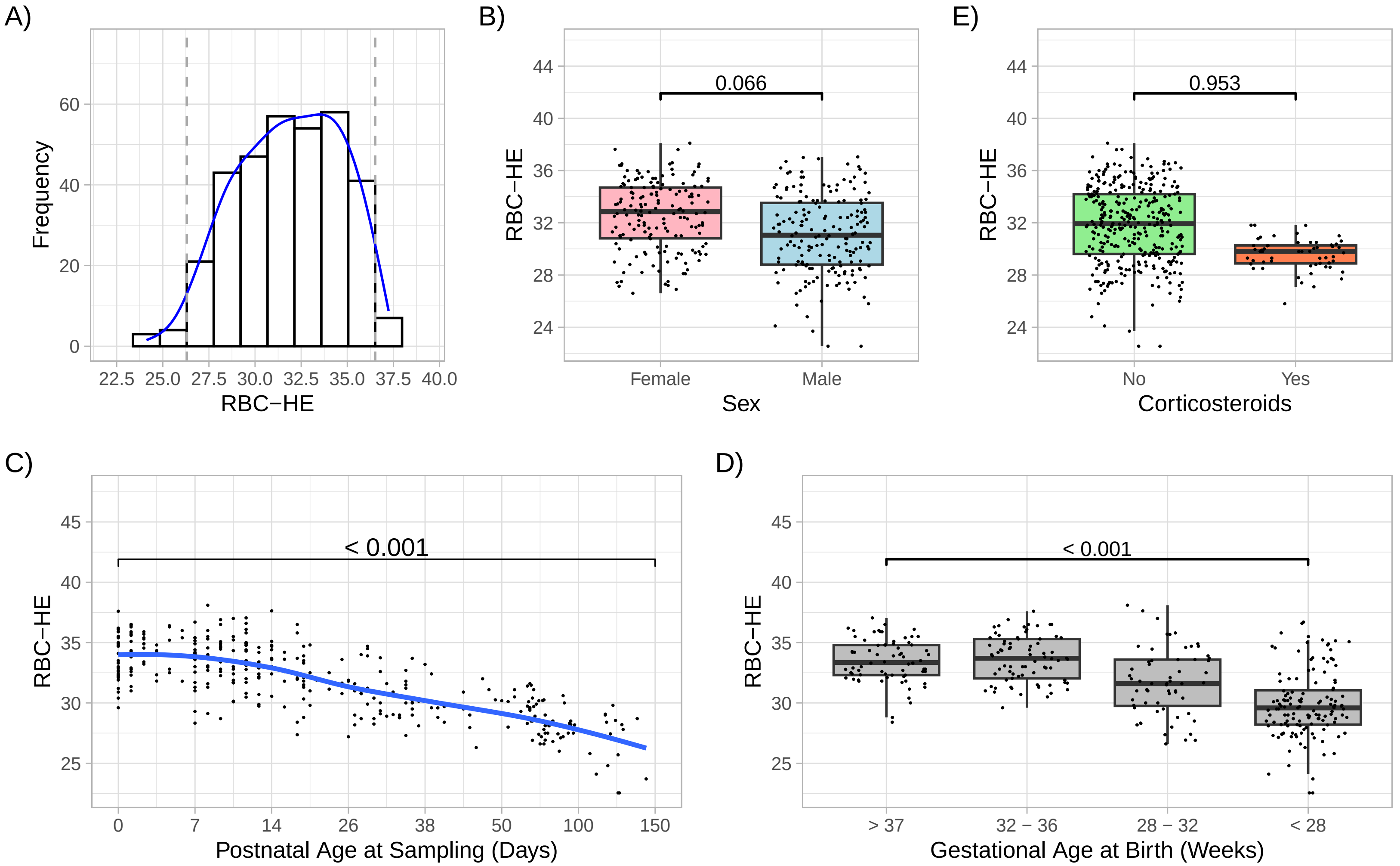

Figure S14: Evaluation of the baseline profile for red blood cell haemoglobin equivalent (pg) in hospitalised but clinically well neonates. (A) Histogram with reference ranges (grey dotted lines) and a distribution curve (blue line). (B) Box plot of sex. (C) Scatter plot of postnatal age at sampling with a fitted curve (blue line). (D) Box plot of gestational age at birth. (E) Box plot of corticosteroid exposure. Corticosteroid exposed values were removed in plots A to D.

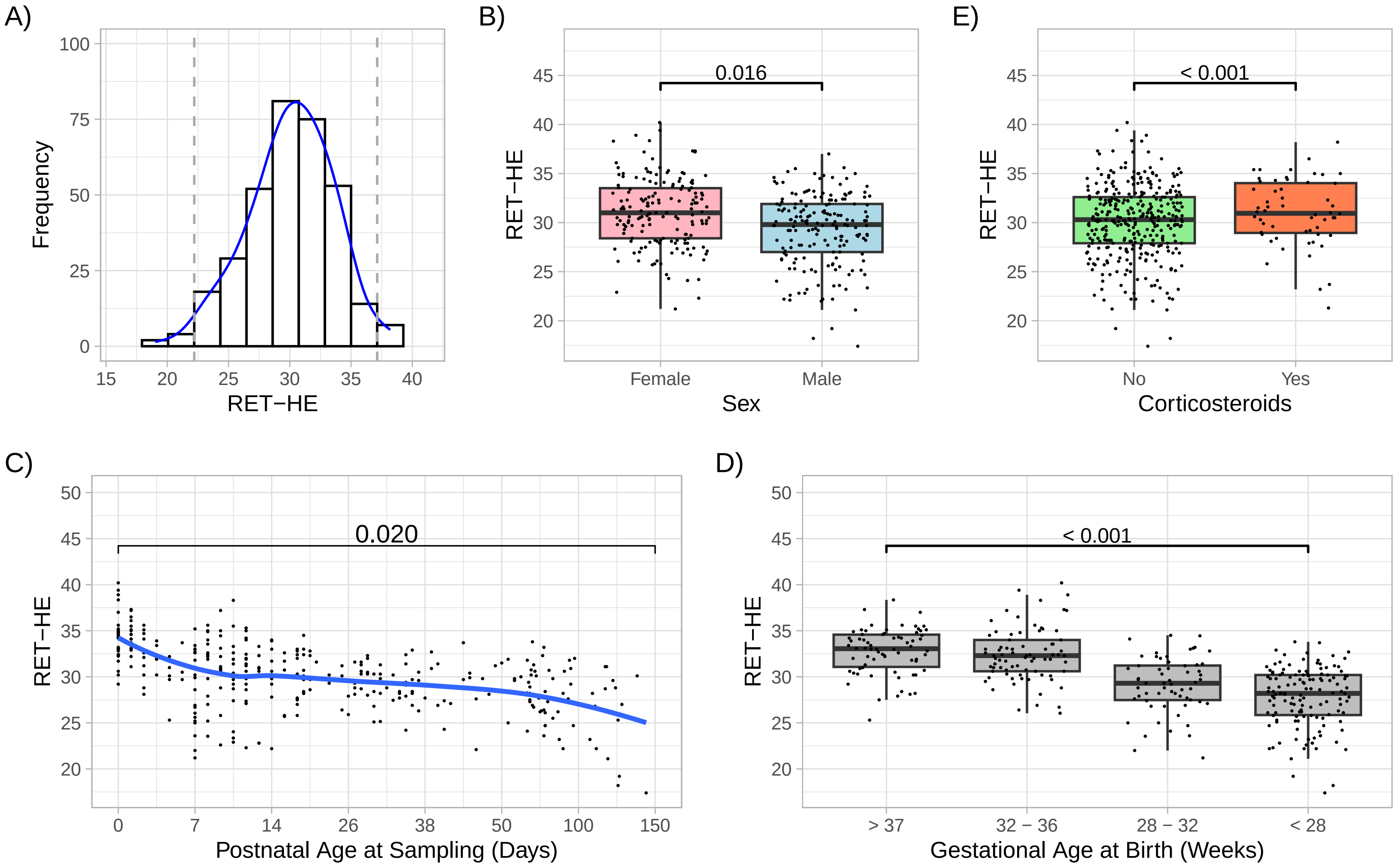

Figure S15: Evaluation of the baseline profile for reticulocyte haemoglobin equivalent (pg) in hospitalised but clinically well neonates. (A) Histogram with reference ranges (grey dotted lines) and a distribution curve (blue line). (B) Box plot of sex. (C) Scatter plot of postnatal age at sampling with a fitted curve (blue line). (D) Box plot of gestational age at birth. (E) Box plot of corticosteroid exposure. Corticosteroid exposed values were removed in plots A to D.

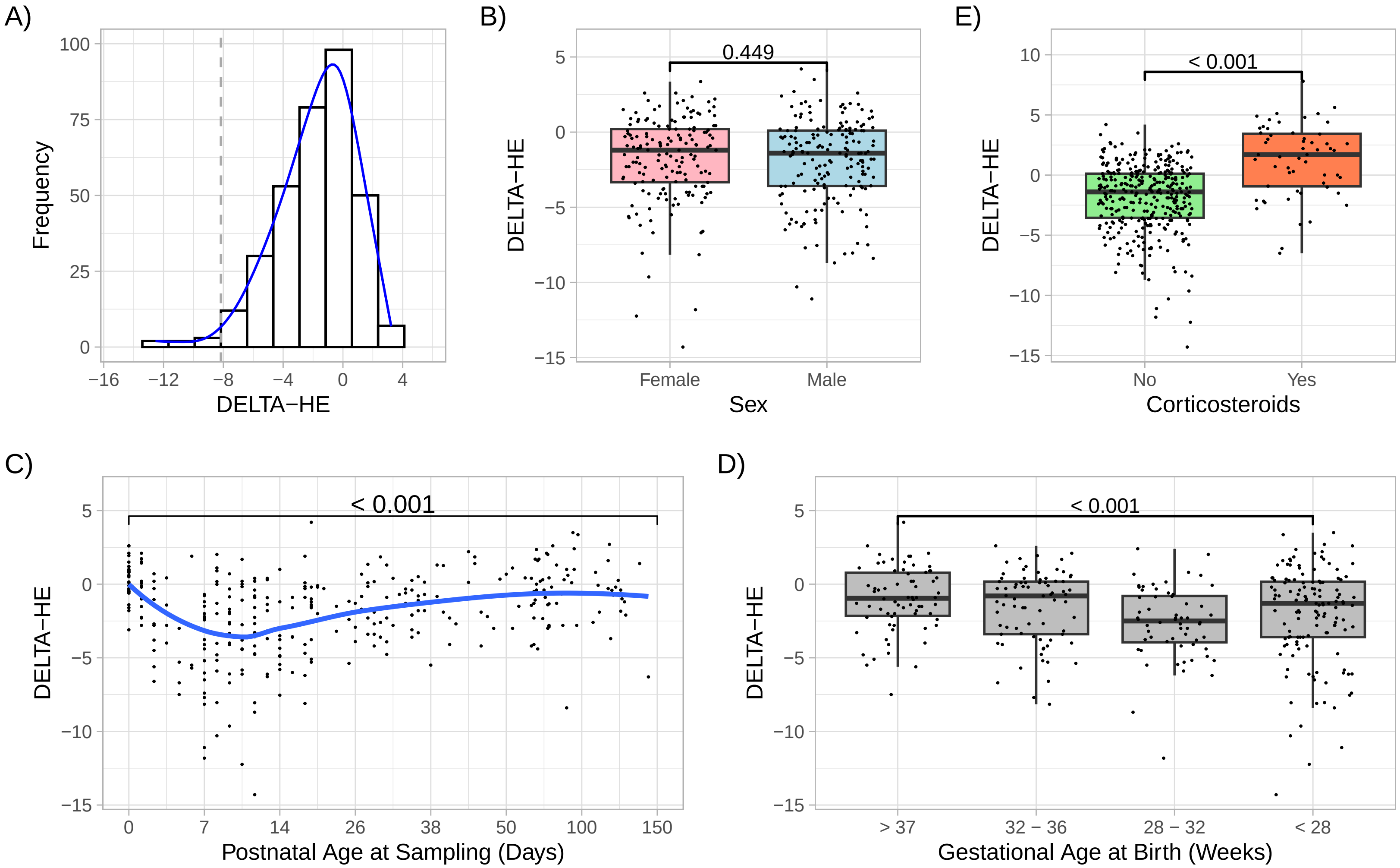

Figure S16: Evaluation of the baseline profile for haemoglobin equivalent difference (pg) in hospitalised but clinically well neonates. (A) Histogram with reference ranges (grey dotted lines) and a distribution curve (blue line). (B) Box plot of sex. (C) Scatter plot of postnatal age at sampling with a fitted curve (blue line). (D) Box plot of gestational age at birth. (E) Box plot of corticosteroid exposure. Corticosteroid exposed values were removed in plots A to D.

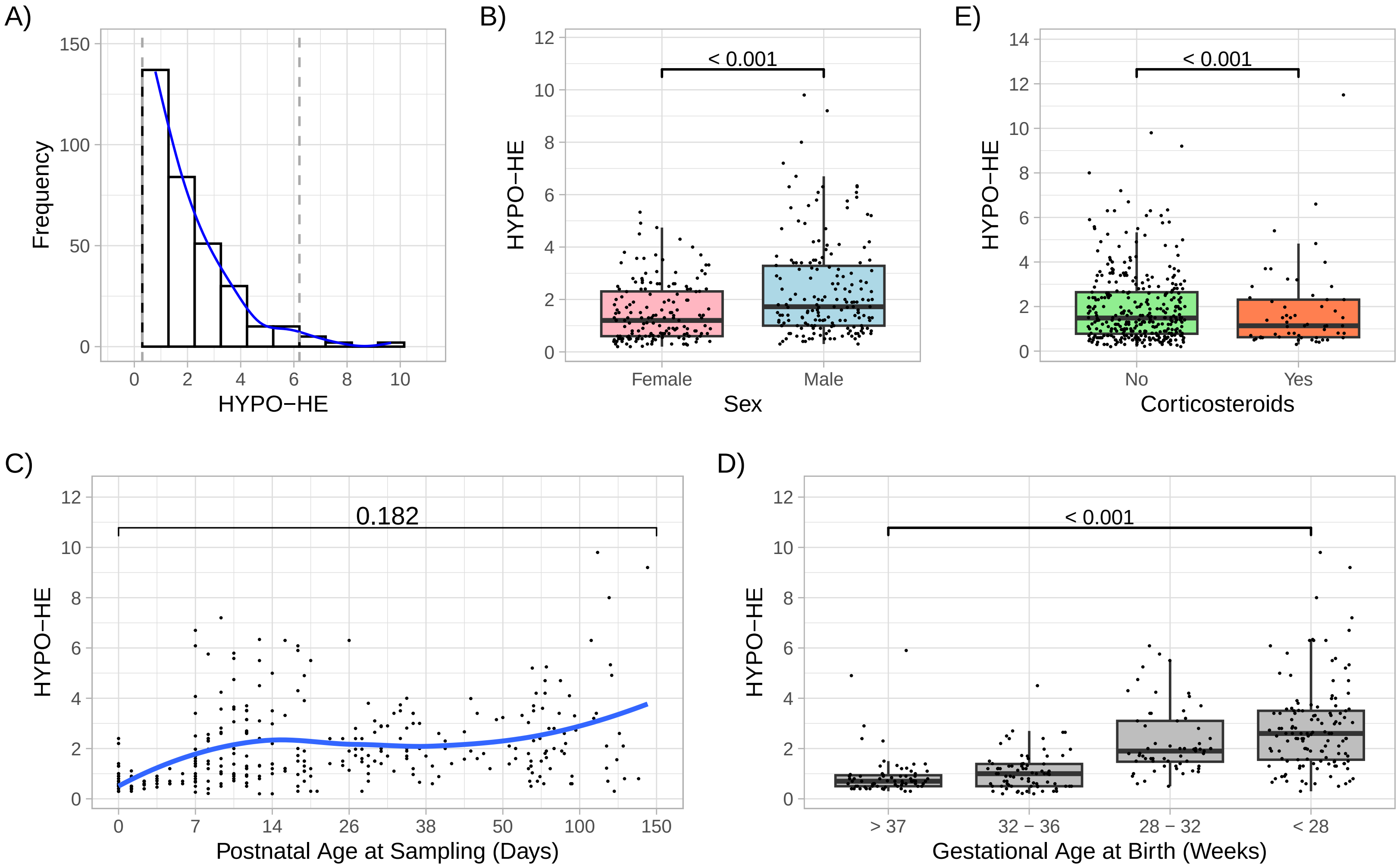

Figure S17: Evaluation of the baseline profile for hypochromic red blood cell count (%) in hospitalised but clinically well neonates. (A) Histogram with reference ranges (grey dotted lines) and a distribution curve (blue line). (B) Box plot of sex. (C) Scatter plot of postnatal age at sampling with a fitted curve (blue line). (D) Box plot of gestational age at birth. (E) Box plot of corticosteroid exposure. Corticosteroid exposed values were removed in plots A to D.

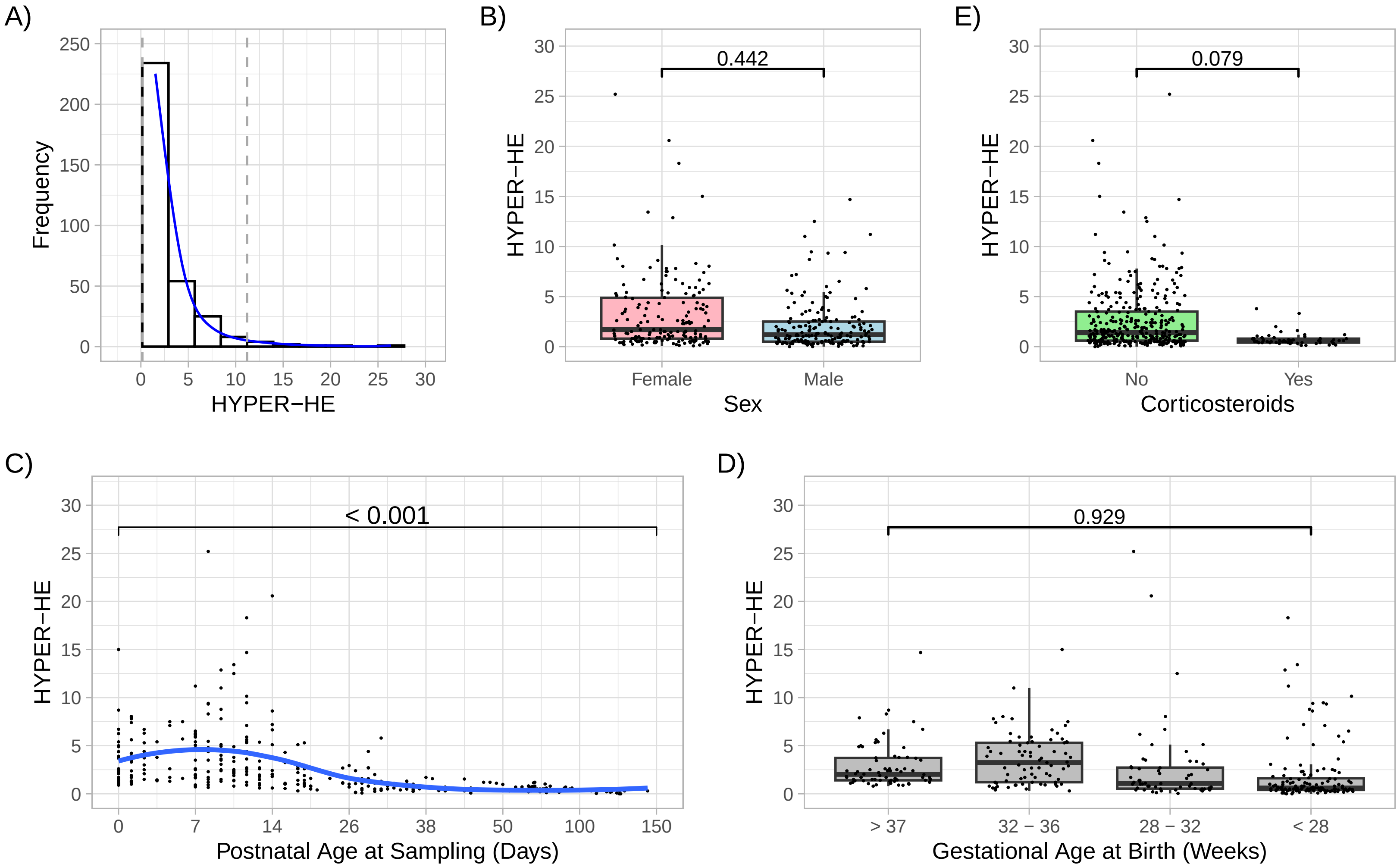

Figure S18: Evaluation of the baseline profile for hyperchromic red blood cell count (%) in hospitalised but clinically well neonates. (A) Histogram with reference ranges (grey dotted lines) and a distribution curve (blue line). (B) Box plot of sex. (C) Scatter plot of postnatal age at sampling with a fitted curve (blue line). (D) Box plot of gestational age at birth. (E) Box plot of corticosteroid exposure. Corticosteroid exposed values were removed in plots A to D.

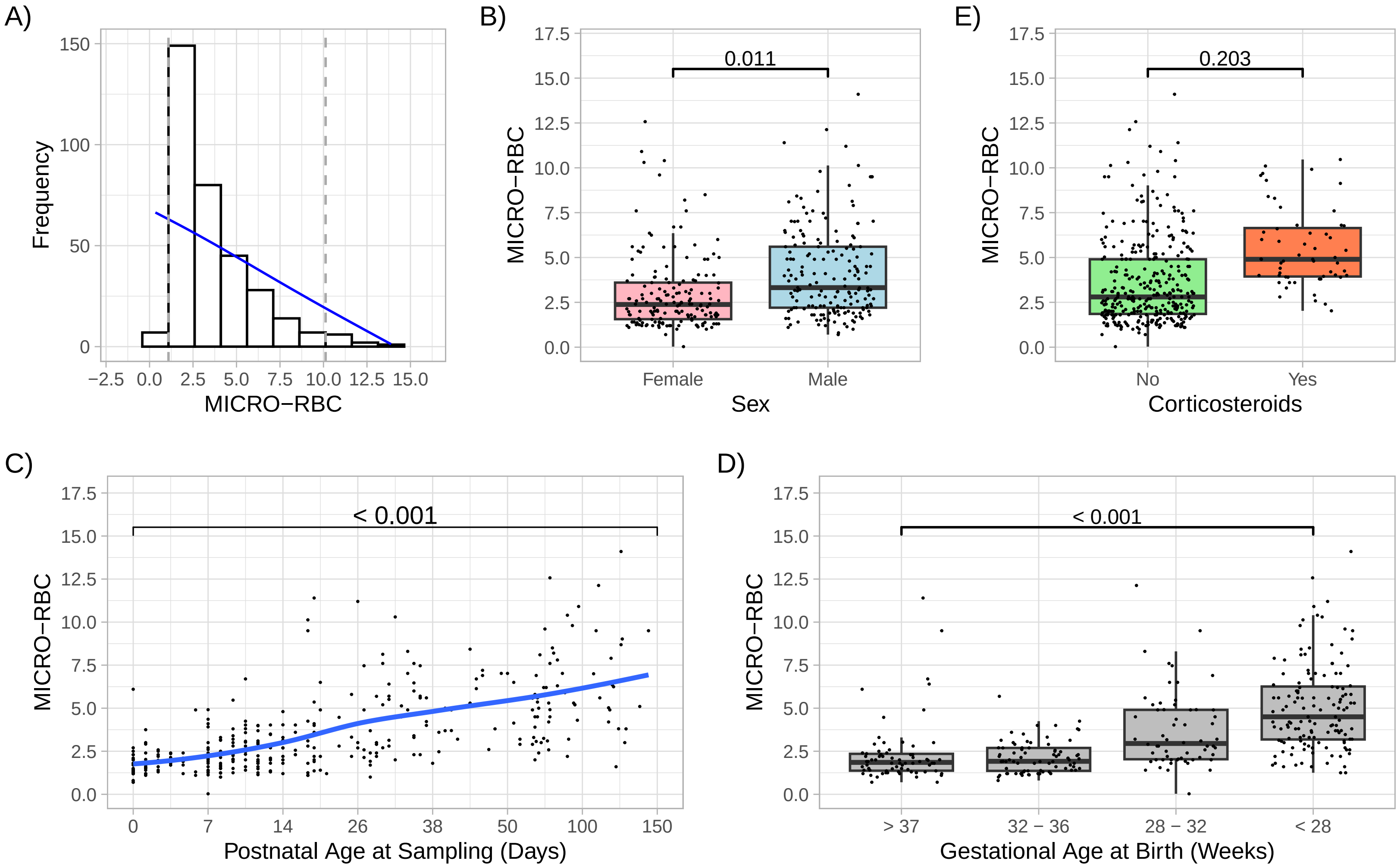

Figure S19: Evaluation of the baseline profile for microcytic red blood cell count (%) in hospitalised but clinically well neonates. (A) Histogram with reference ranges (grey dotted lines) and a distribution curve (blue line). (B) Box plot of sex. (C) Scatter plot of postnatal age at sampling with a fitted curve (blue line). (D) Box plot of gestational age at birth. (E) Box plot of corticosteroid exposure. Corticosteroid exposed values were removed in plots A to D.

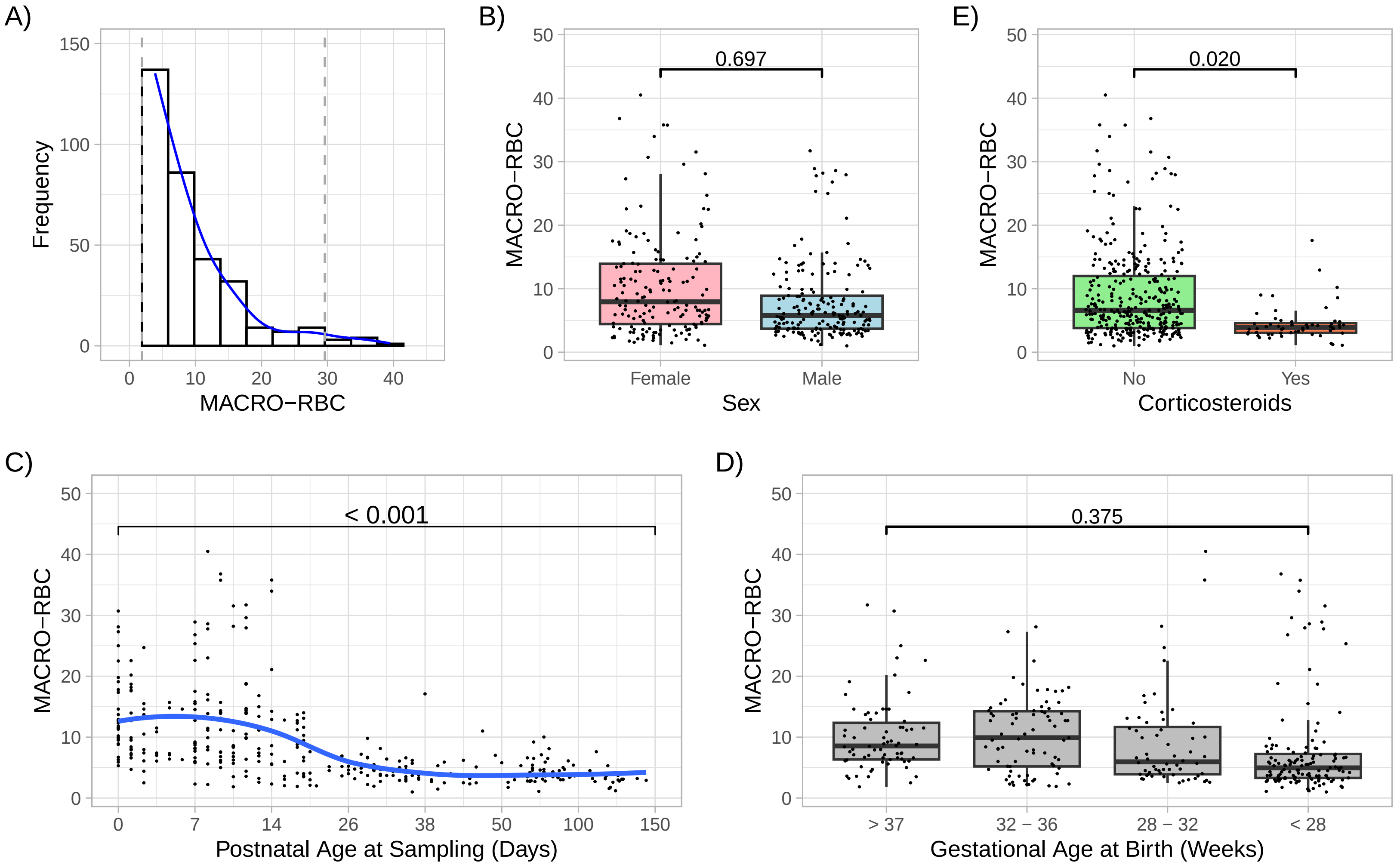

Figure S20: Evaluation of the baseline profile for macrocytic red blood cell count (%) in hospitalised but clinically well neonates. (A) Histogram with reference ranges (grey dotted lines) and a distribution curve (blue line). (B) Box plot of sex. (C) Scatter plot of postnatal age at sampling with a fitted curve (blue line). (D) Box plot of gestational age at birth. (E) Box plot of corticosteroid exposure. Corticosteroid exposed values were removed in plots A to D.

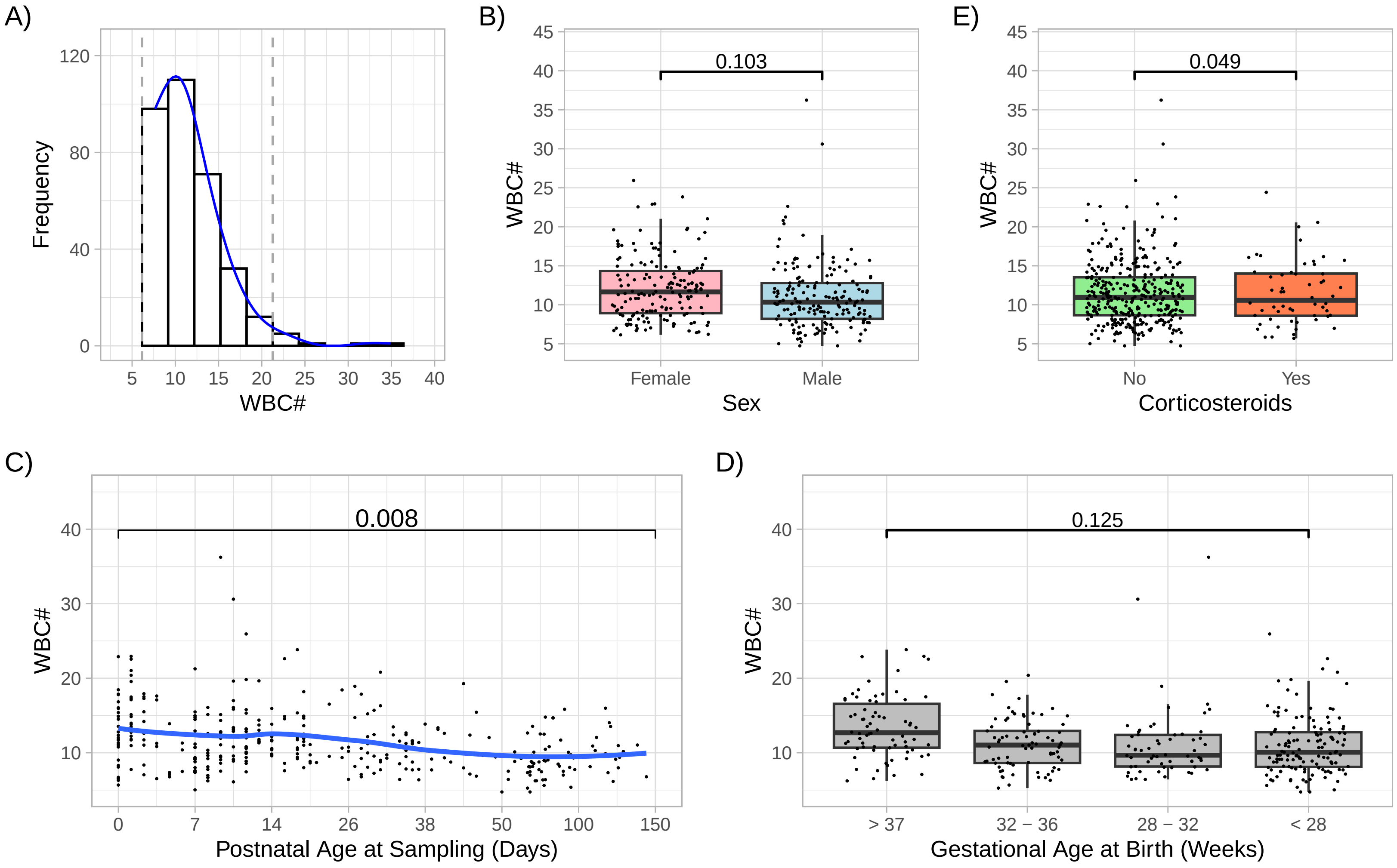

Figure S21: Evaluation of the baseline profile for white blood cell count (x10^9/L) in hospitalised but clinically well neonates. (A) Histogram with reference ranges (grey dotted lines) and a distribution curve (blue line). (B) Box plot of sex. (C) Scatter plot of postnatal age at sampling with a fitted curve (blue line). (D) Box plot of gestational age at birth. (E) Box plot of corticosteroid exposure. Corticosteroid exposed values were removed in plots A to D.

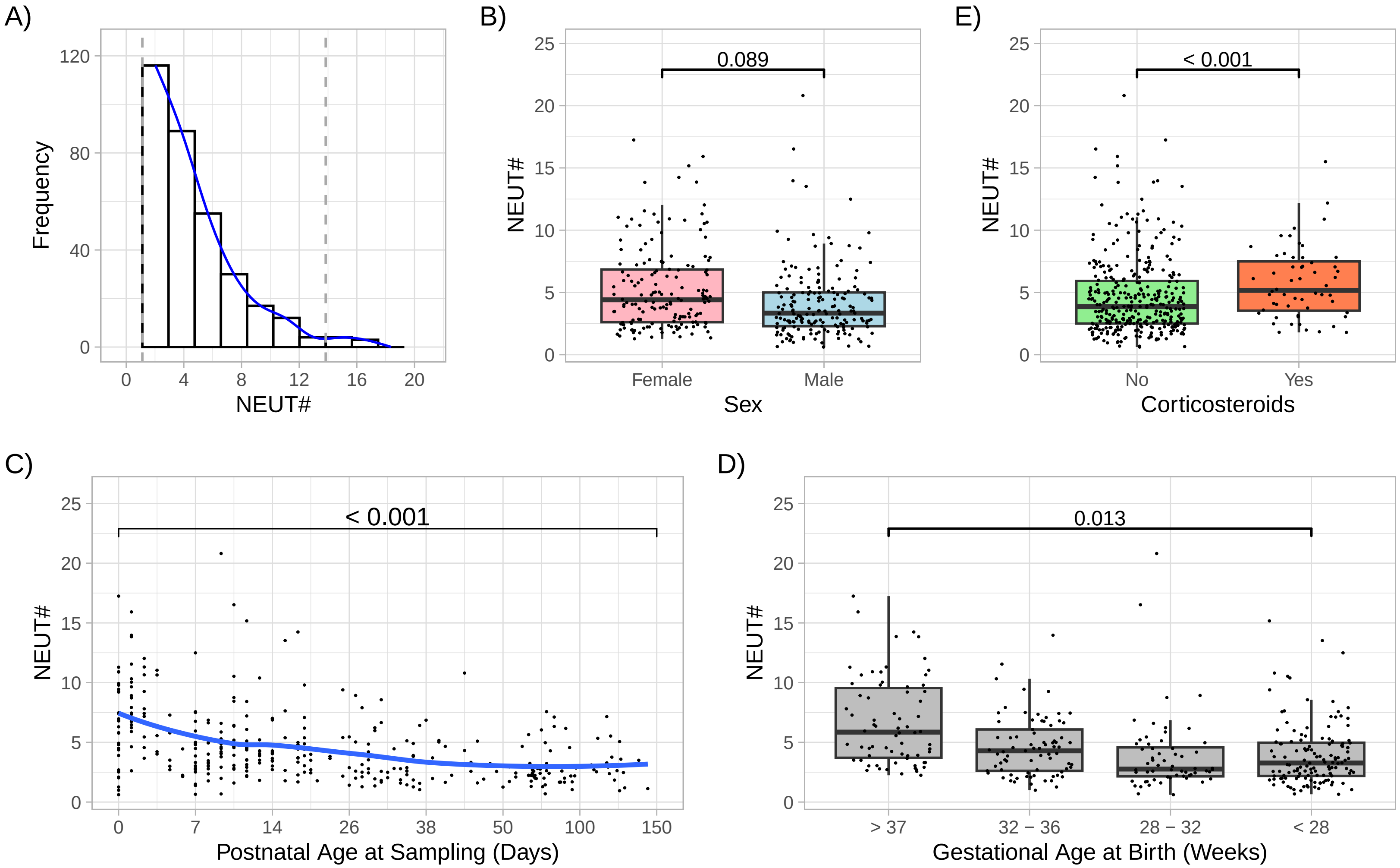

Figure S22: Evaluation of the baseline profile for neutrophil count (x10^9/L) in hospitalised but clinically well neonates. (A) Histogram with reference ranges (grey dotted lines) and a distribution curve (blue line). (B) Box plot of sex. (C) Scatter plot of postnatal age at sampling with a fitted curve (blue line). (D) Box plot of gestational age at birth. (E) Box plot of corticosteroid exposure. Corticosteroid exposed values were removed in plots A to D.

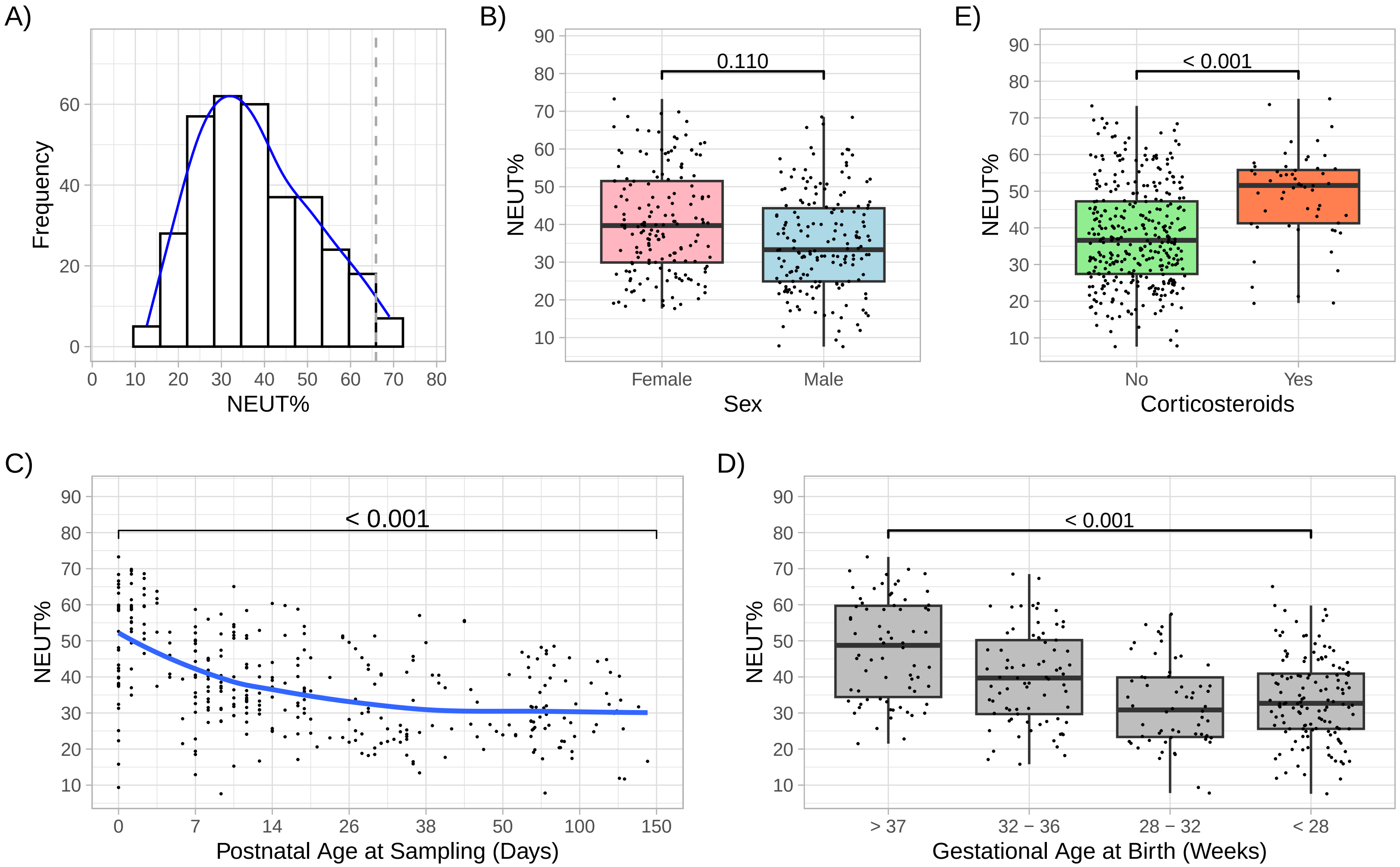

Figure S23: Evaluation of the baseline profile for neutrophil count (%) in hospitalised but clinically well neonates. (A) Histogram with reference ranges (grey dotted lines) and a distribution curve (blue line). (B) Box plot of sex. (C) Scatter plot of postnatal age at sampling with a fitted curve (blue line). (D) Box plot of gestational age at birth. (E) Box plot of corticosteroid exposure. Corticosteroid exposed values were removed in plots A to D.

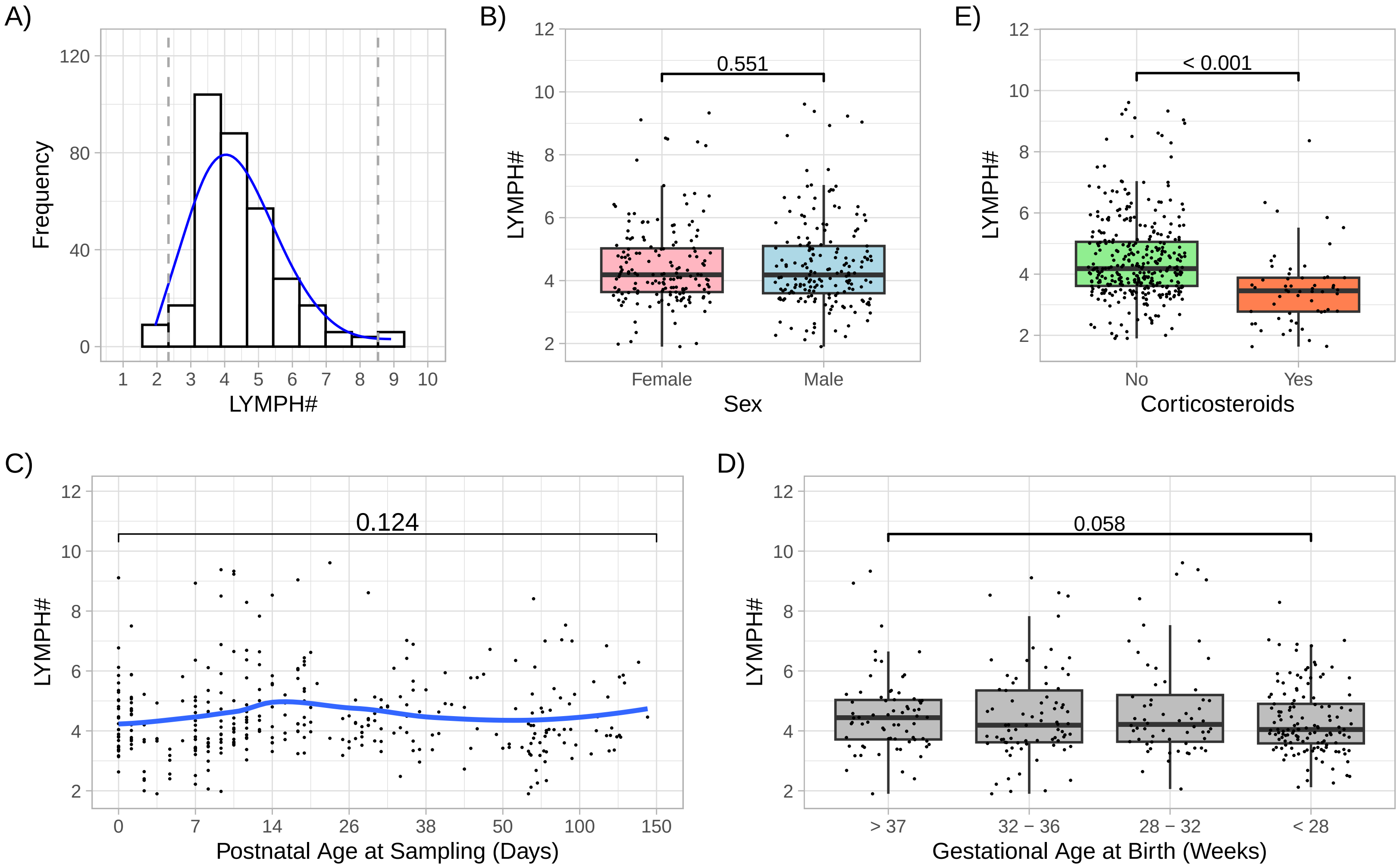

Figure S24: Evaluation of the baseline profile for lymphocyte count (x10^9/L) in hospitalised but clinically well neonates. (A) Histogram with reference ranges (grey dotted lines) and a distribution curve (blue line). (B) Box plot of sex. (C) Scatter plot of postnatal age at sampling with a fitted curve (blue line). (D) Box plot of gestational age at birth. (E) Box plot of corticosteroid exposure. Corticosteroid exposed values were removed in plots A to D.

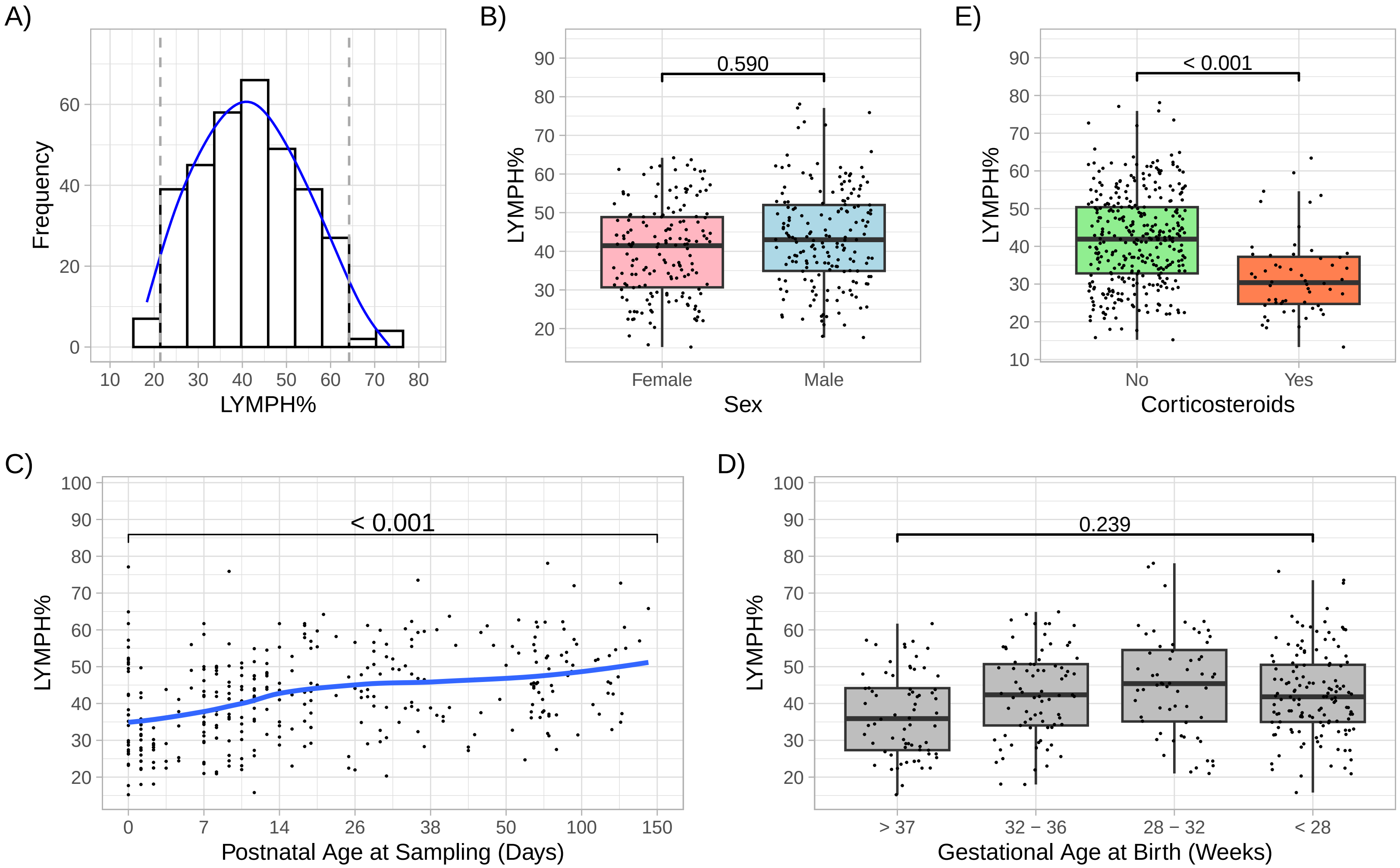

Figure S25: Evaluation of the baseline profile for lymphocyte count (%) in hospitalised but clinically well neonates. (A) Histogram with reference ranges (grey dotted lines) and a distribution curve (blue line). (B) Box plot of sex. (C) Scatter plot of postnatal age at sampling with a fitted curve (blue line). (D) Box plot of gestational age at birth. (E) Box plot of corticosteroid exposure. Corticosteroid exposed values were removed in plots A to D.

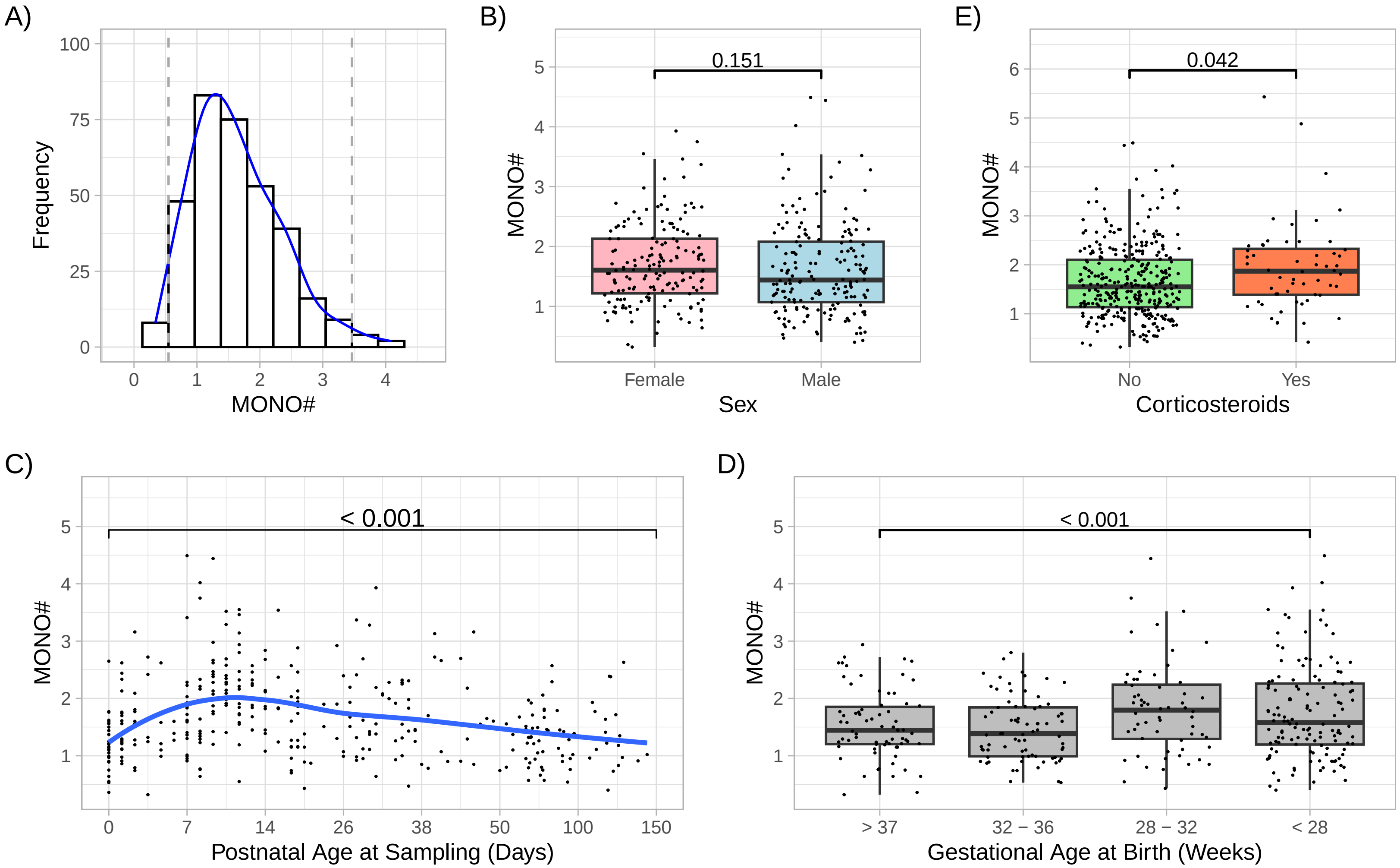

Figure S26: Evaluation of the baseline profile for monocyte count (x10^9/L) in hospitalised but clinically well neonates. (A) Histogram with reference ranges (grey dotted lines) and a distribution curve (blue line). (B) Box plot of sex. (C) Scatter plot of postnatal age at sampling with a fitted curve (blue line). (D) Box plot of gestational age at birth. (E) Box plot of corticosteroid exposure. Corticosteroid exposed values were removed in plots A to D.

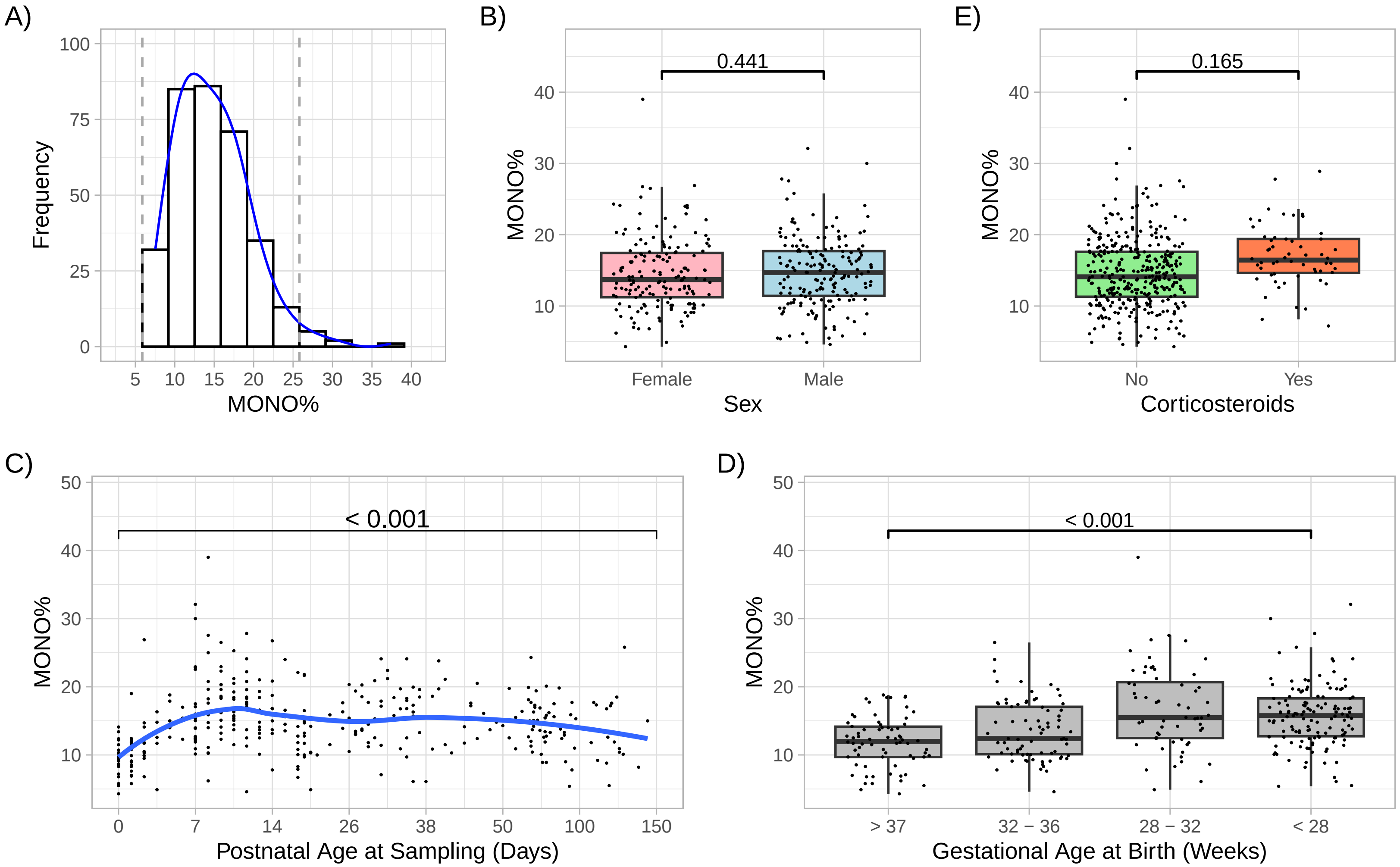

Figure S27: Evaluation of the baseline profile for monocyte count (%) in hospitalised but clinically well neonates. (A) Histogram with reference ranges (grey dotted lines) and a distribution curve (blue line). (B) Box plot of sex. (C) Scatter plot of postnatal age at sampling with a fitted curve (blue line). (D) Box plot of gestational age at birth. (E) Box plot of corticosteroid exposure. Corticosteroid exposed values were removed in plots A to D.

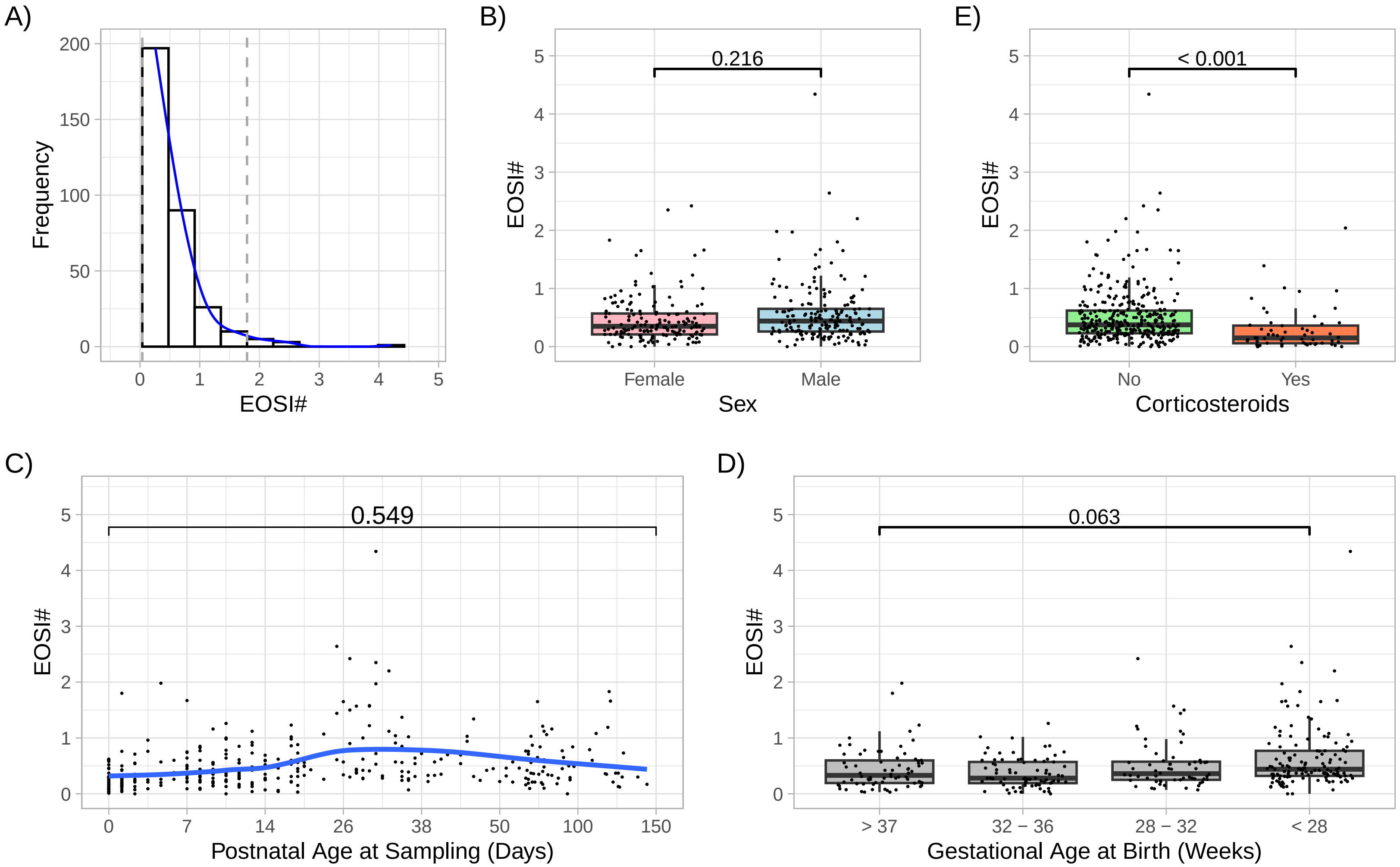

Figure S28: Evaluation of the baseline profile for eosinophil count (x10^9/L) in hospitalised but clinically well neonates. (A) Histogram with reference ranges (grey dotted lines) and a distribution curve (blue line). (B) Box plot of sex. (C) Scatter plot of postnatal age at sampling with a fitted curve (blue line). (D) Box plot of gestational age at birth. (E) Box plot of corticosteroid exposure. Corticosteroid exposed values were removed in plots A to D.

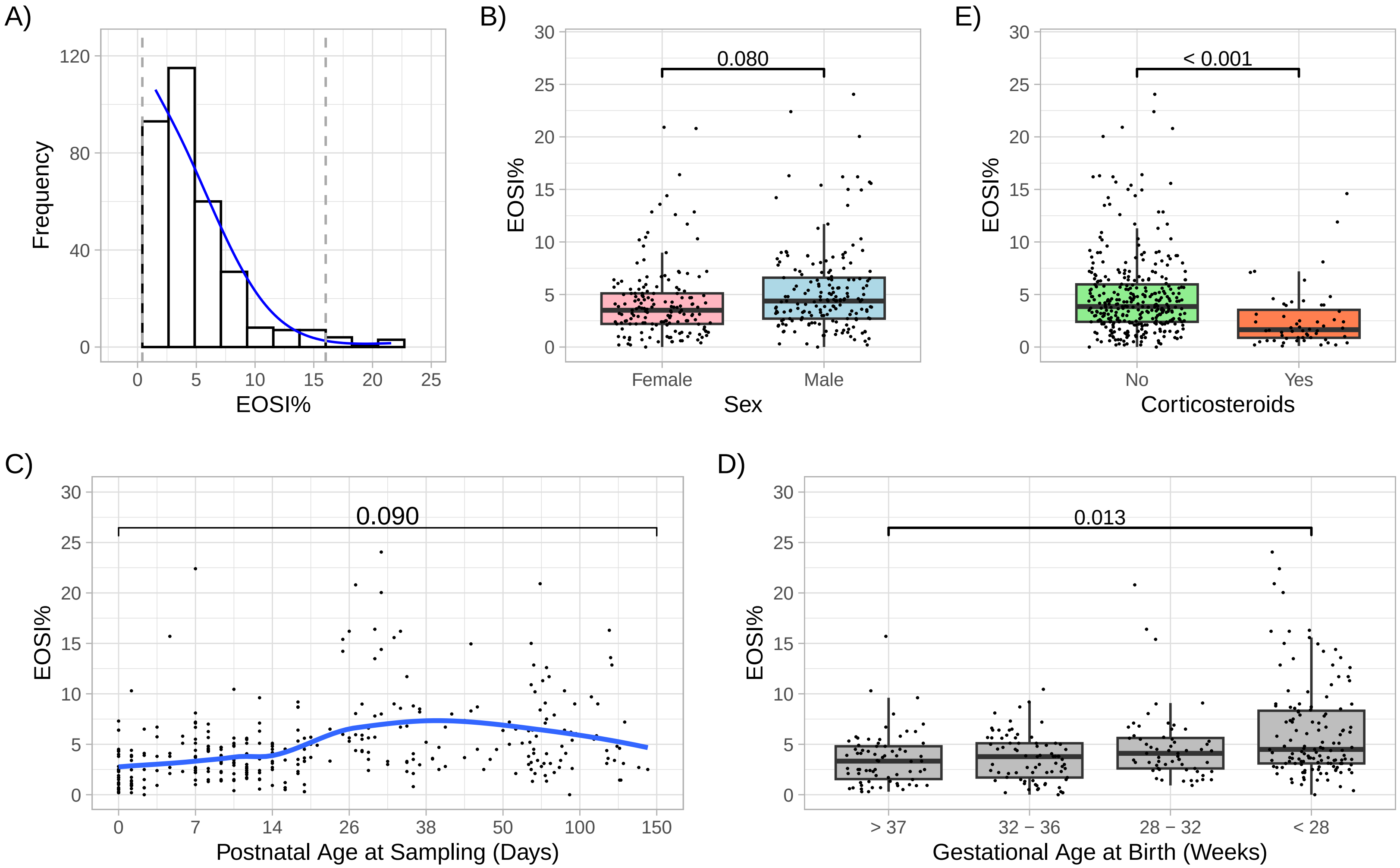

Figure S29: Evaluation of the baseline profile for eosinophil count (%) in hospitalised but clinically well neonates. (A) Histogram with reference ranges (grey dotted lines) and a distribution curve (blue line). (B) Box plot of sex. (C) Scatter plot of postnatal age at sampling with a fitted curve (blue line). (D) Box plot of gestational age at birth. (E) Box plot of corticosteroid exposure. Corticosteroid exposed values were removed in plots A to D.

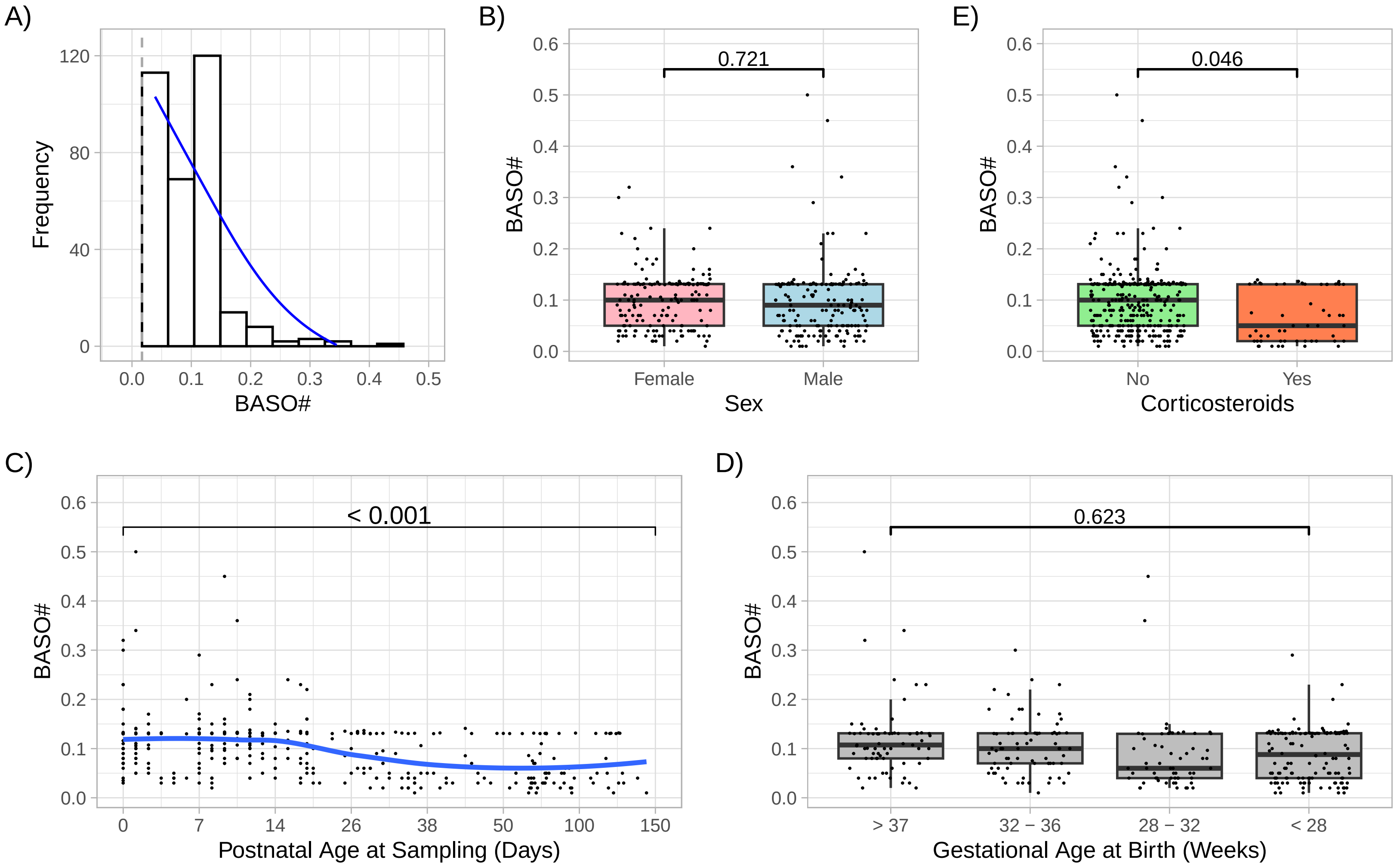

Figure S30: Evaluation of the baseline profile for basophil count (x10^9/L) in hospitalised but clinically well neonates. (A) Histogram with reference ranges (grey dotted lines) and a distribution curve (blue line). (B) Box plot of sex. (C) Scatter plot of postnatal age at sampling with a fitted curve (blue line). (D) Box plot of gestational age at birth. (E) Box plot of corticosteroid exposure. Corticosteroid exposed values were removed in plots A to D.

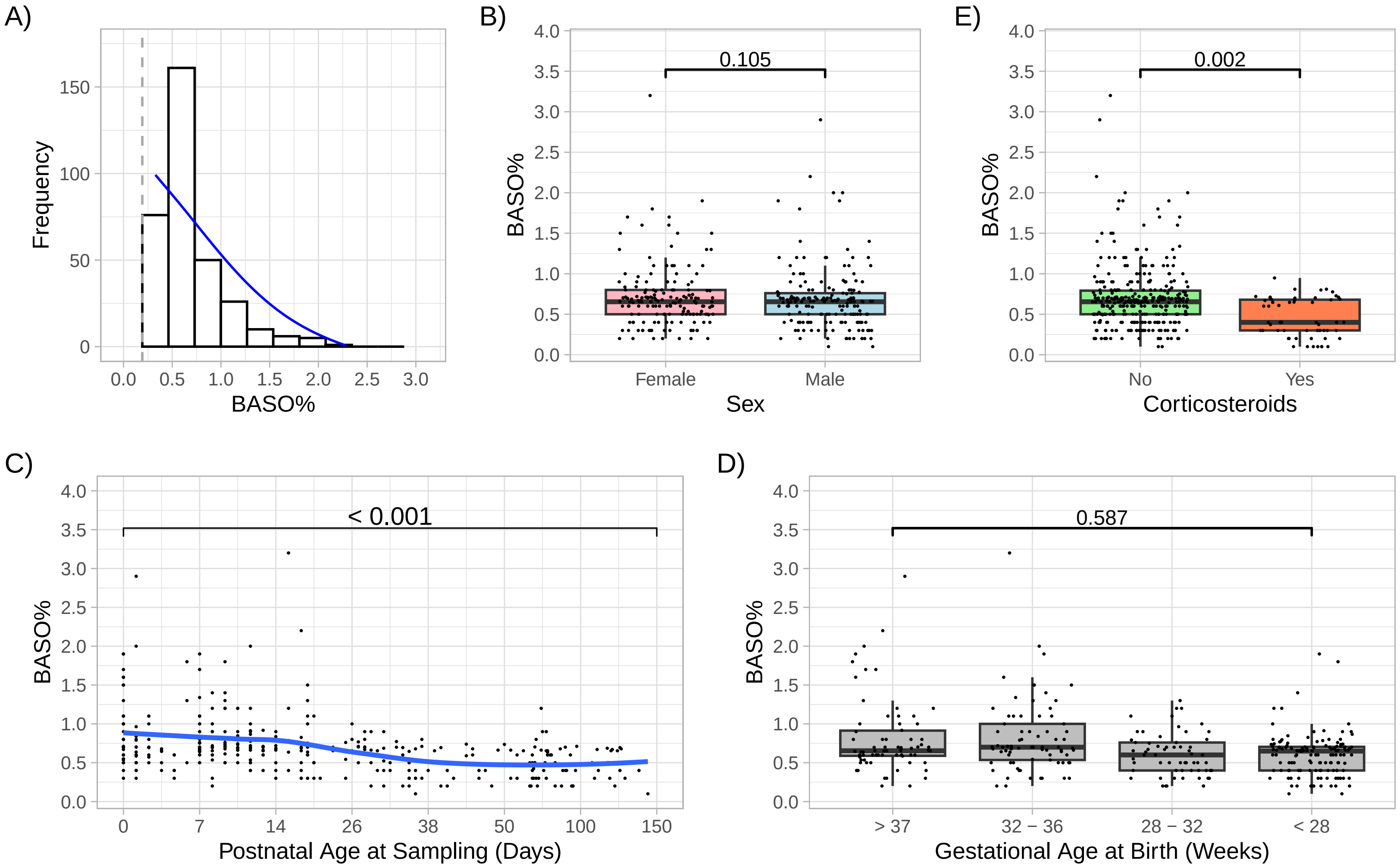

Figure S31: Evaluation of the baseline profile for basophil count (%) in hospitalised but clinically well neonates. (A) Histogram with reference ranges (grey dotted lines) and a distribution curve (blue line). (B) Box plot of sex. (C) Scatter plot of postnatal age at sampling with a fitted curve (blue line). (D) Box plot of gestational age at birth. (E) Box plot of corticosteroid exposure. Corticosteroid exposed values were removed in plots A to D.

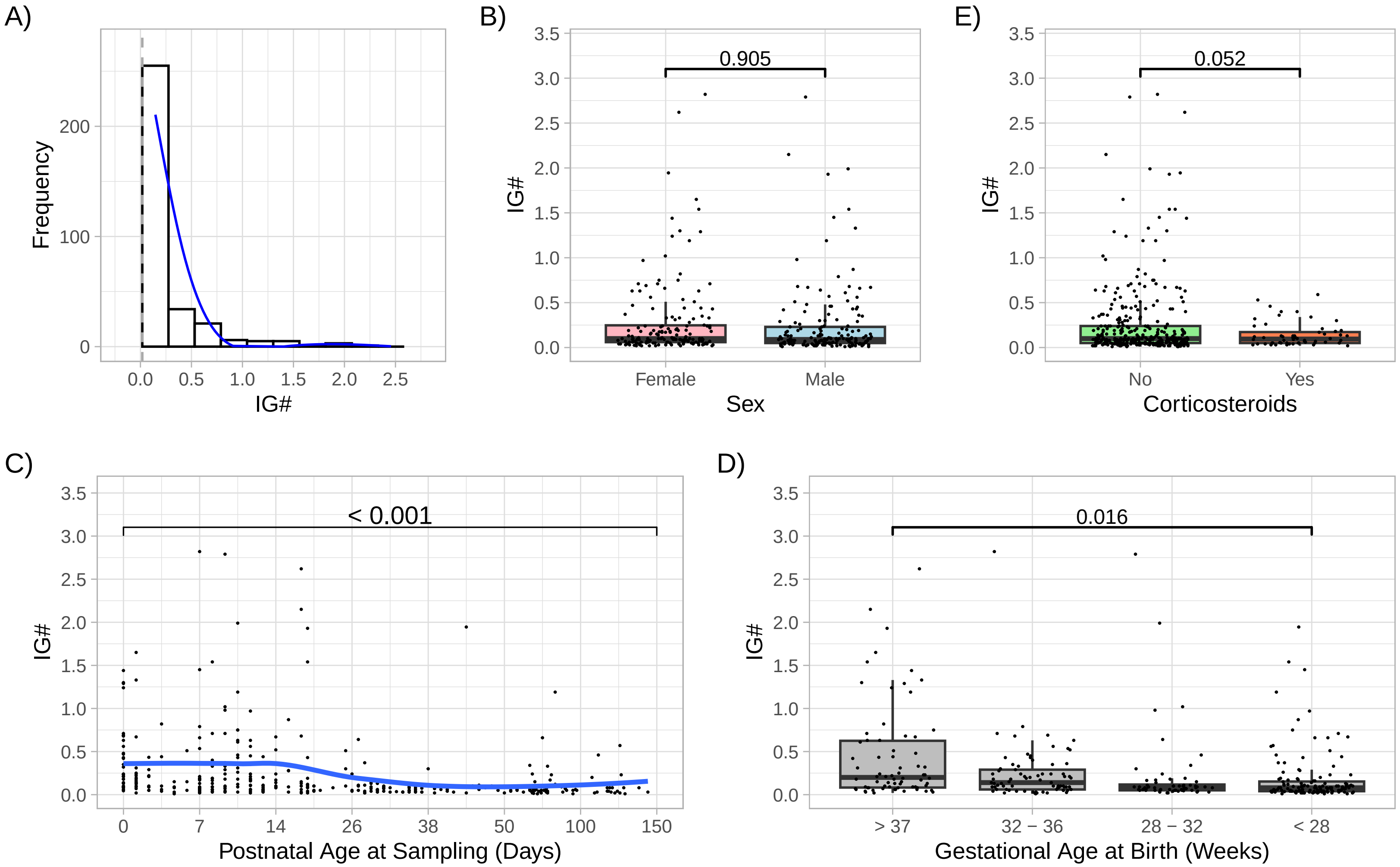

Figure S32: Evaluation of the baseline profile for immature granulocyte count (x10^9/L) in hospitalised but clinically well neonates. (A) Histogram with reference ranges (grey dotted lines) and a distribution curve (blue line). (B) Box plot of sex. (C) Scatter plot of postnatal age at sampling with a fitted curve (blue line). (D) Box plot of gestational age at birth. (E) Box plot of corticosteroid exposure. Corticosteroid exposed values were removed in plots A to D.

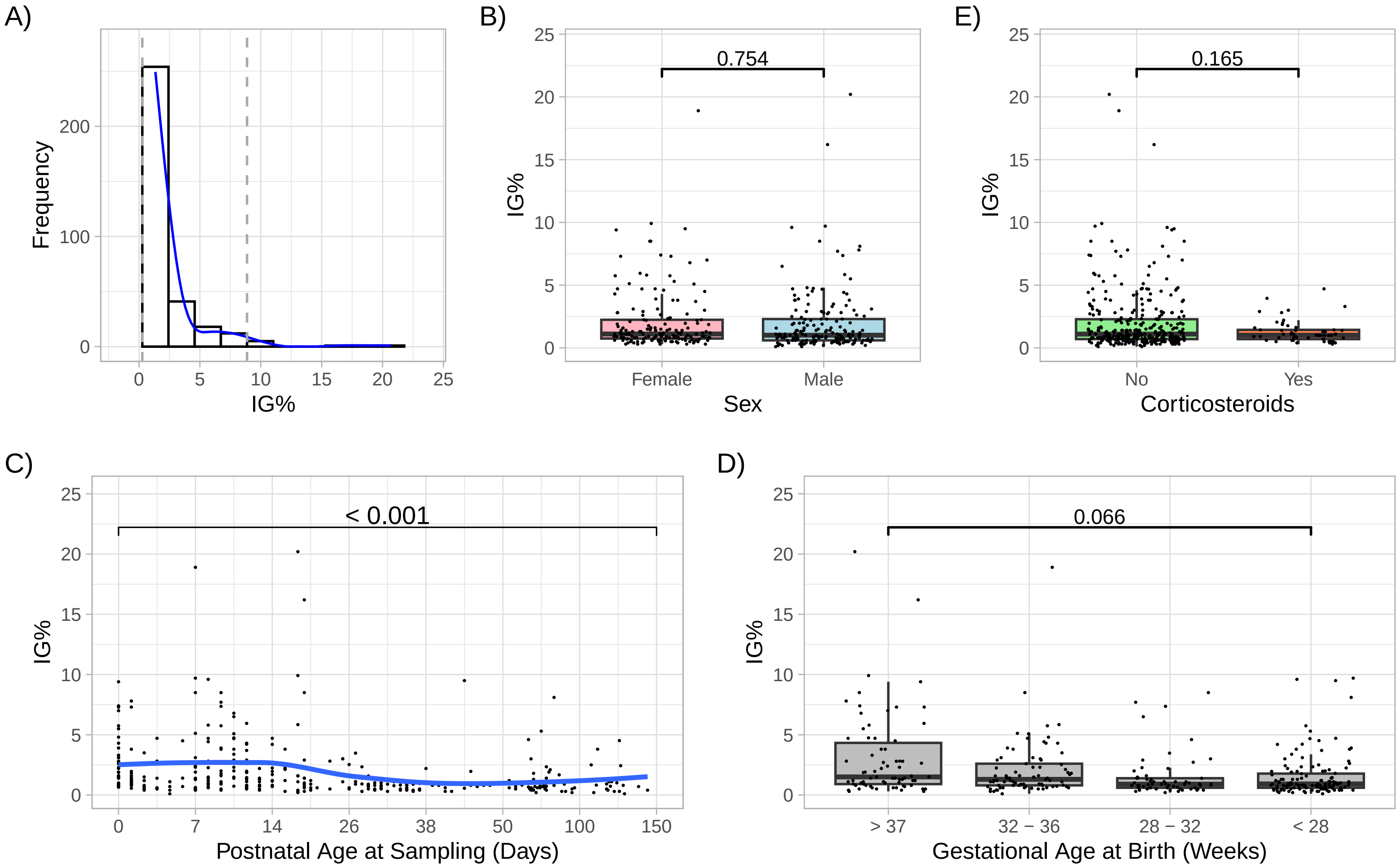

Figure S33: Evaluation of the baseline profile for immature granulocyte count (%) in hospitalised but clinically well neonates. (A) Histogram with reference ranges (grey dotted lines) and a distribution curve (blue line). (B) Box plot of sex. (C) Scatter plot of postnatal age at sampling with a fitted curve (blue line). (D) Box plot of gestational age at birth. (E) Box plot of corticosteroid exposure. Corticosteroid exposed values were removed in plots A to D.

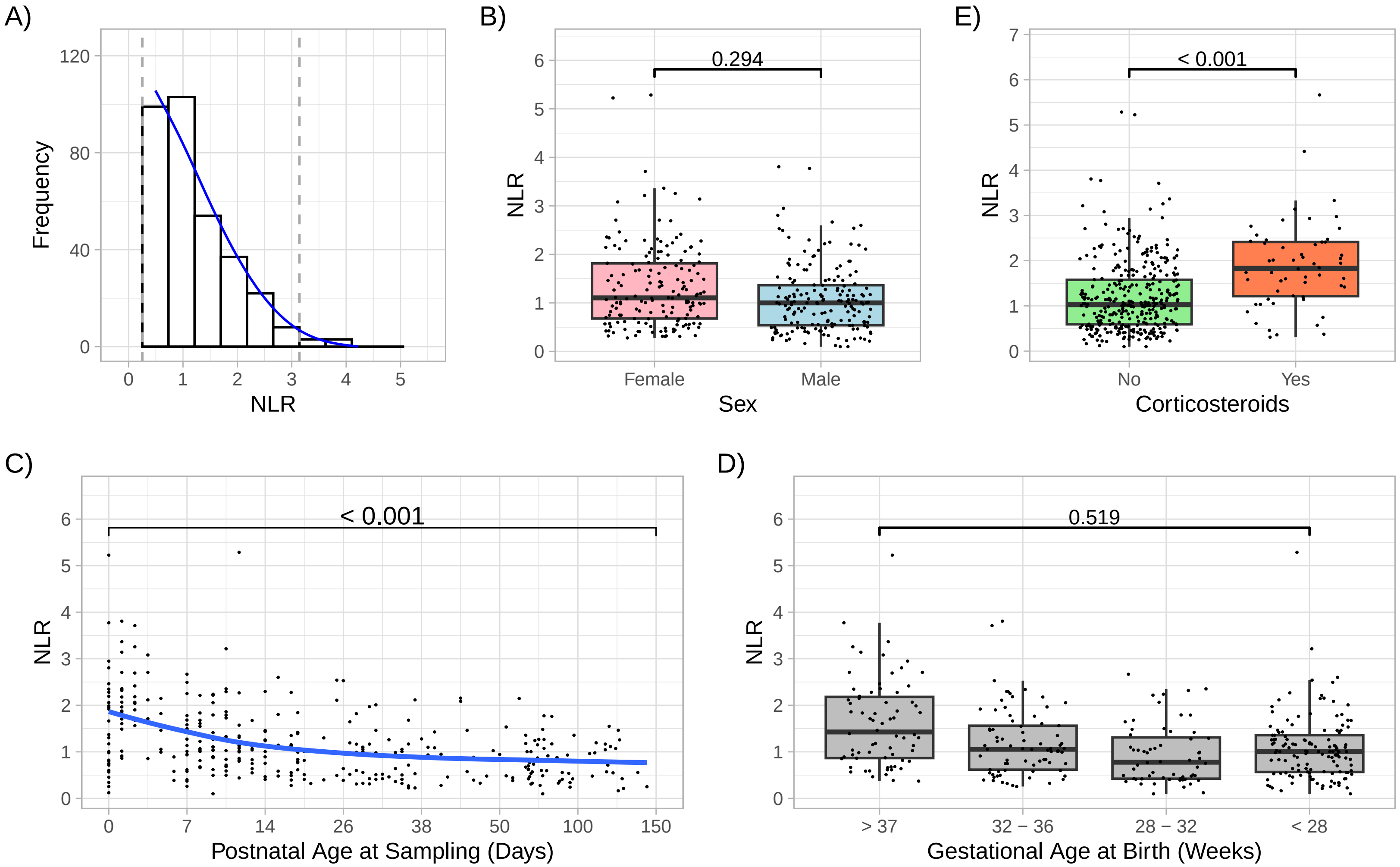

Figure S34: Evaluation of the baseline profile for neutrophil lymphocyte ratio (%) in hospitalised but clinically well neonates. (A) Histogram with reference ranges (grey dotted lines) and a distribution curve (blue line). (B) Box plot of sex. (C) Scatter plot of postnatal age at sampling with a fitted curve (blue line). (D) Box plot of gestational age at birth. (E) Box plot of corticosteroid exposure. Corticosteroid exposed values were removed in plots A to D.

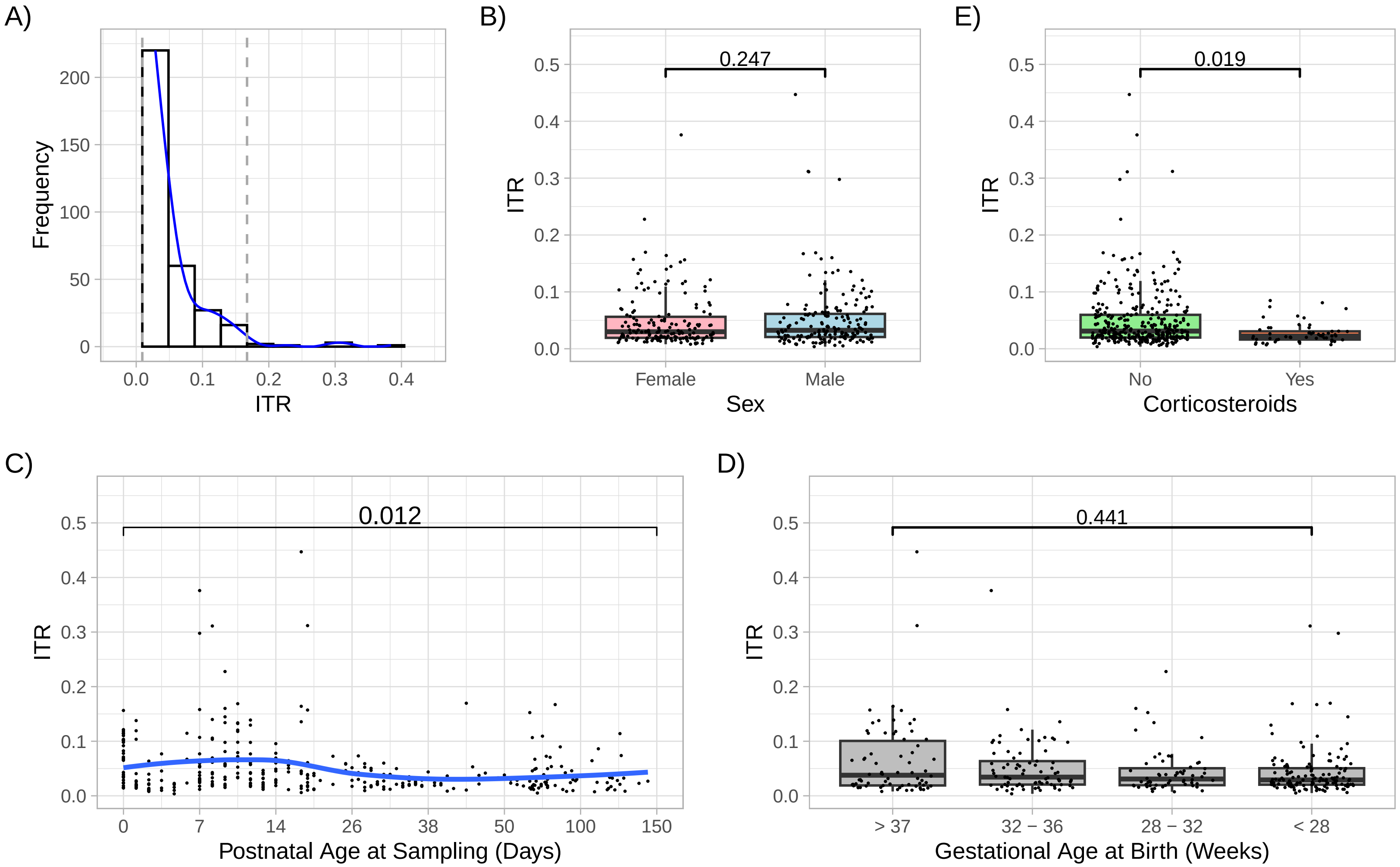

Figure S35: Evaluation of the baseline profile for immature to total neutrophil ratio (%) in hospitalised but clinically well neonates. (A) Histogram with reference ranges (grey dotted lines) and a distribution curve (blue line). (B) Box plot of sex. (C) Scatter plot of postnatal age at sampling with a fitted curve (blue line). (D) Box plot of gestational age at birth. (E) Box plot of corticosteroid exposure. Corticosteroid exposed values were removed in plots A to D.

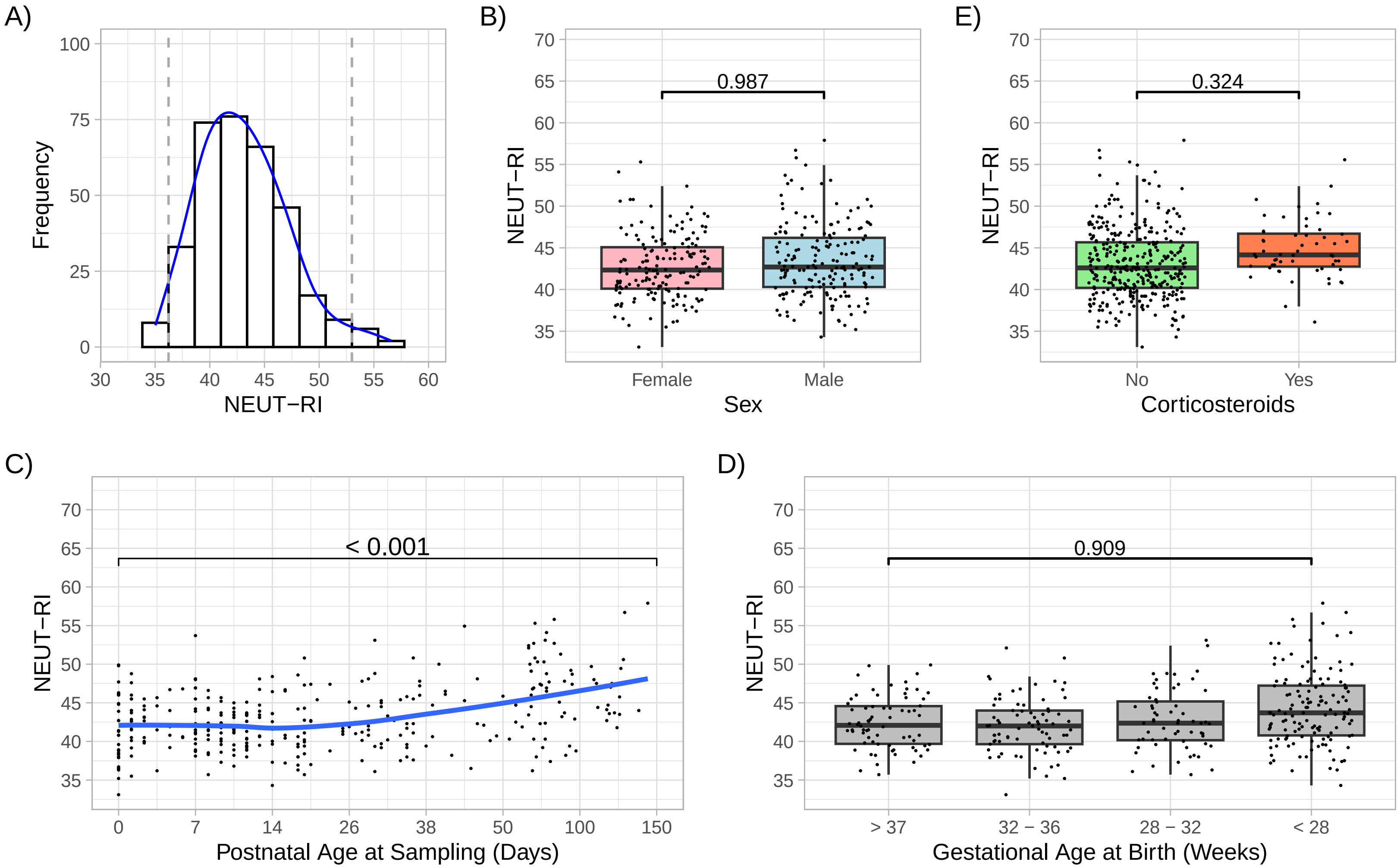

Figure S36: Evaluation of the baseline profile for neutrophil reactive intensity (FI) in hospitalised but clinically well neonates. (A) Histogram with reference ranges (grey dotted lines) and a distribution curve (blue line). (B) Box plot of sex. (C) Scatter plot of postnatal age at sampling with a fitted curve (blue line). (D) Box plot of gestational age at birth. (E) Box plot of corticosteroid exposure. Corticosteroid exposed values were removed in plots A to D.

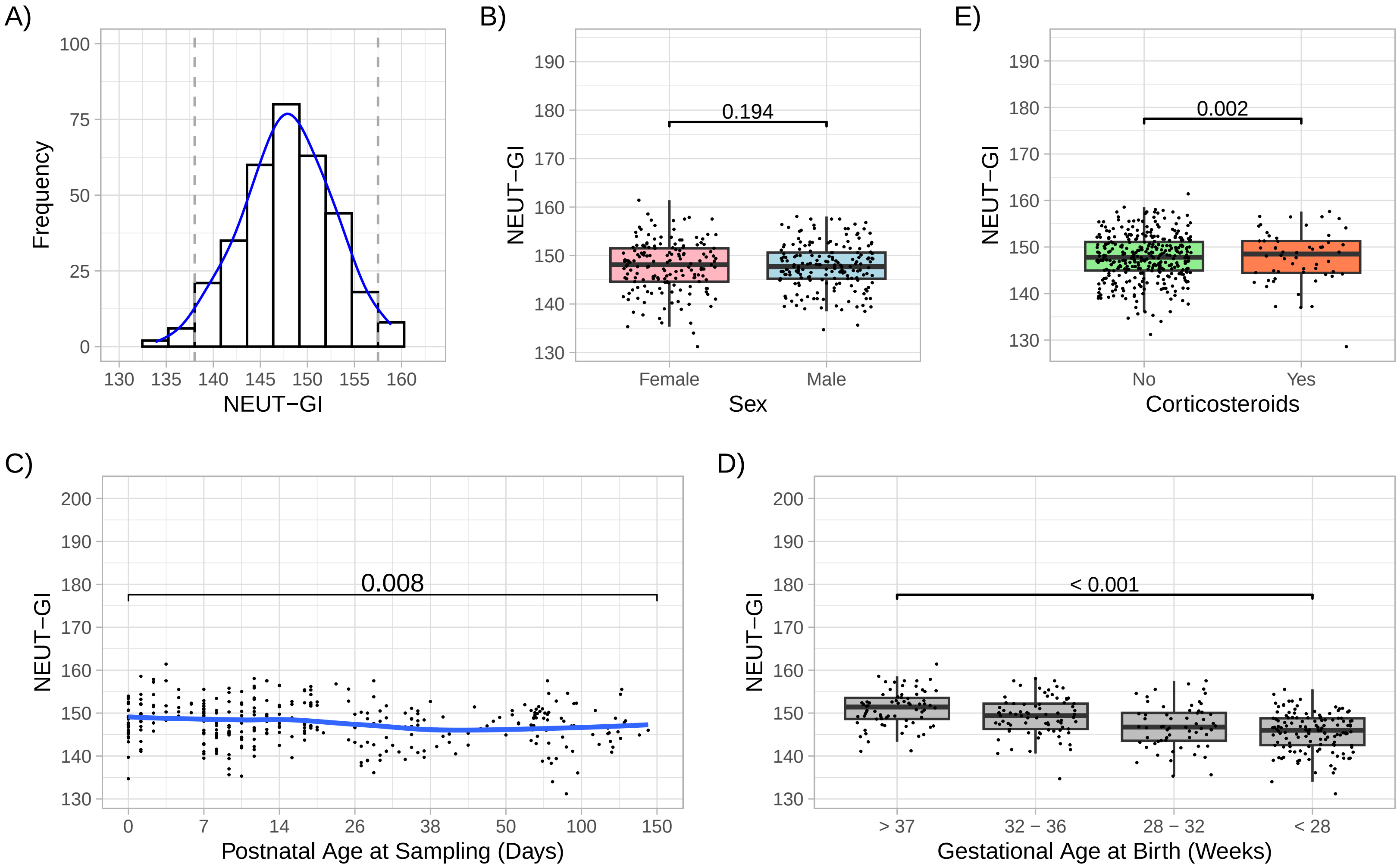

Figure S37: Evaluation of the baseline profile for neutrophil granularity intensity (SI) in hospitalised but clinically well neonates. (A) Histogram with reference ranges (grey dotted lines) and a distribution curve (blue line). (B) Box plot of sex. (C) Scatter plot of postnatal age at sampling with a fitted curve (blue line). (D) Box plot of gestational age at birth. (E) Box plot of corticosteroid exposure. Corticosteroid exposed values were removed in plots A to D.

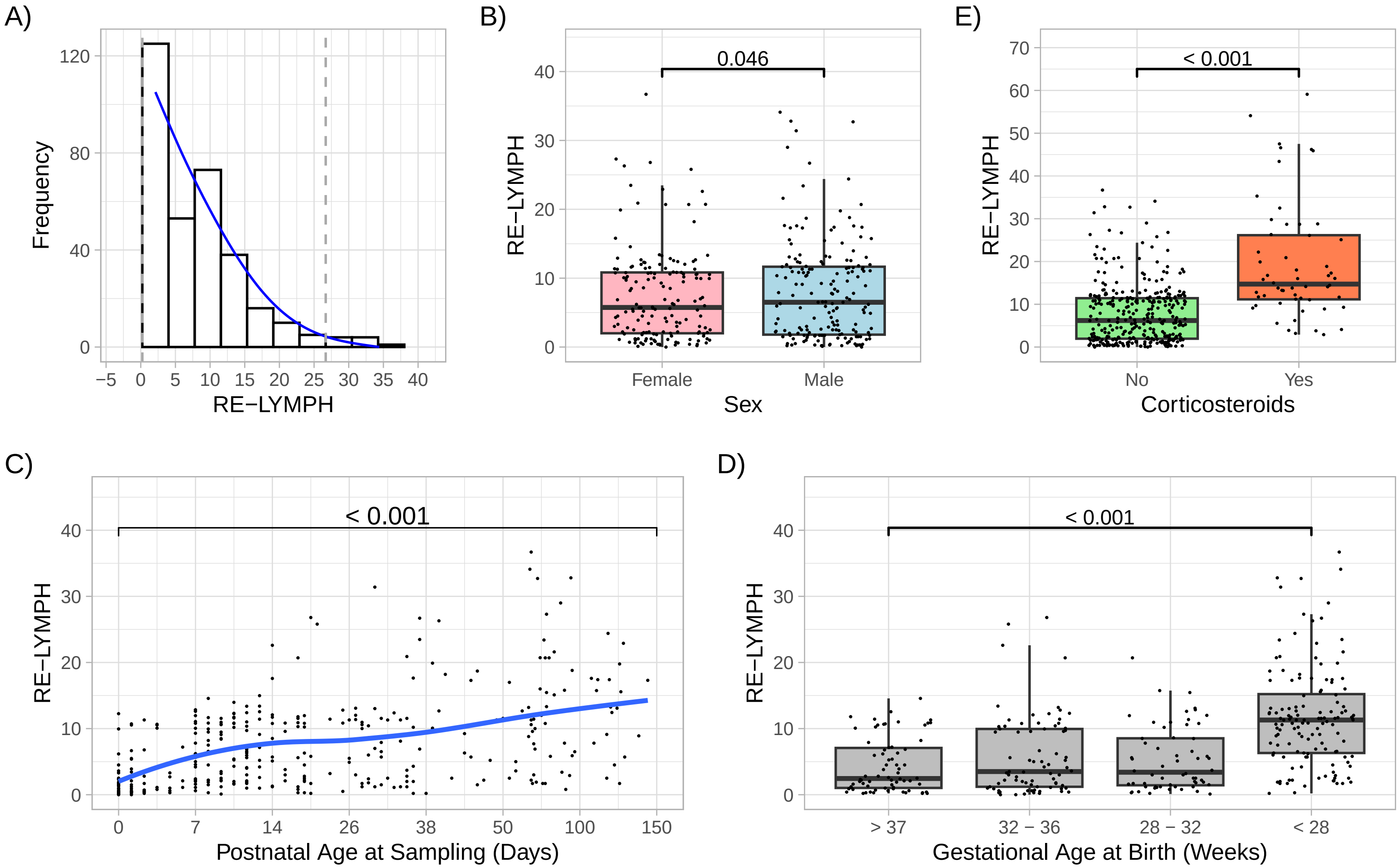

Figure S38: Evaluation of the baseline profile for reactive lymphocyte count (%L) in hospitalised but clinically well neonates. (A) Histogram with reference ranges (grey dotted lines) and a distribution curve (blue line). (B) Box plot of sex. (C) Scatter plot of postnatal age at sampling with a fitted curve (blue line). (D) Box plot of gestational age at birth. (E) Box plot of corticosteroid exposure. Corticosteroid exposed values were removed in plots A to D.

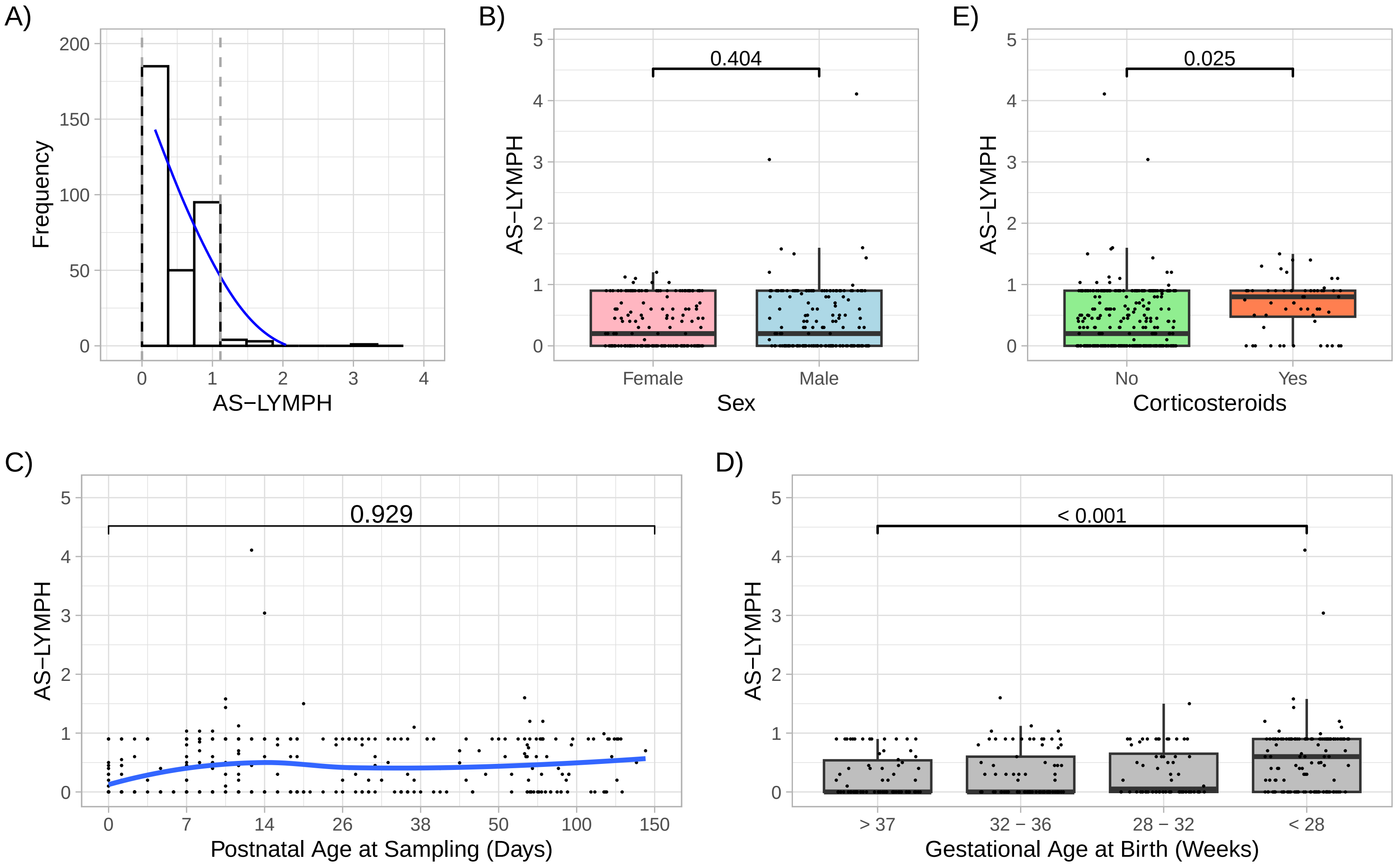

Figure S39: Evaluation of the baseline profile for antibody−synthesising lymphocyte count (%L) in hospitalised but clinically well neonates. (A) Histogram with reference ranges (grey dotted lines) and a distribution curve (blue line). (B) Box plot of sex. (C) Scatter plot of postnatal age at sampling with a fitted curve (blue line). (D) Box plot of gestational age at birth. (E) Box plot of corticosteroid exposure. Corticosteroid exposed values were removed in plots A to D.

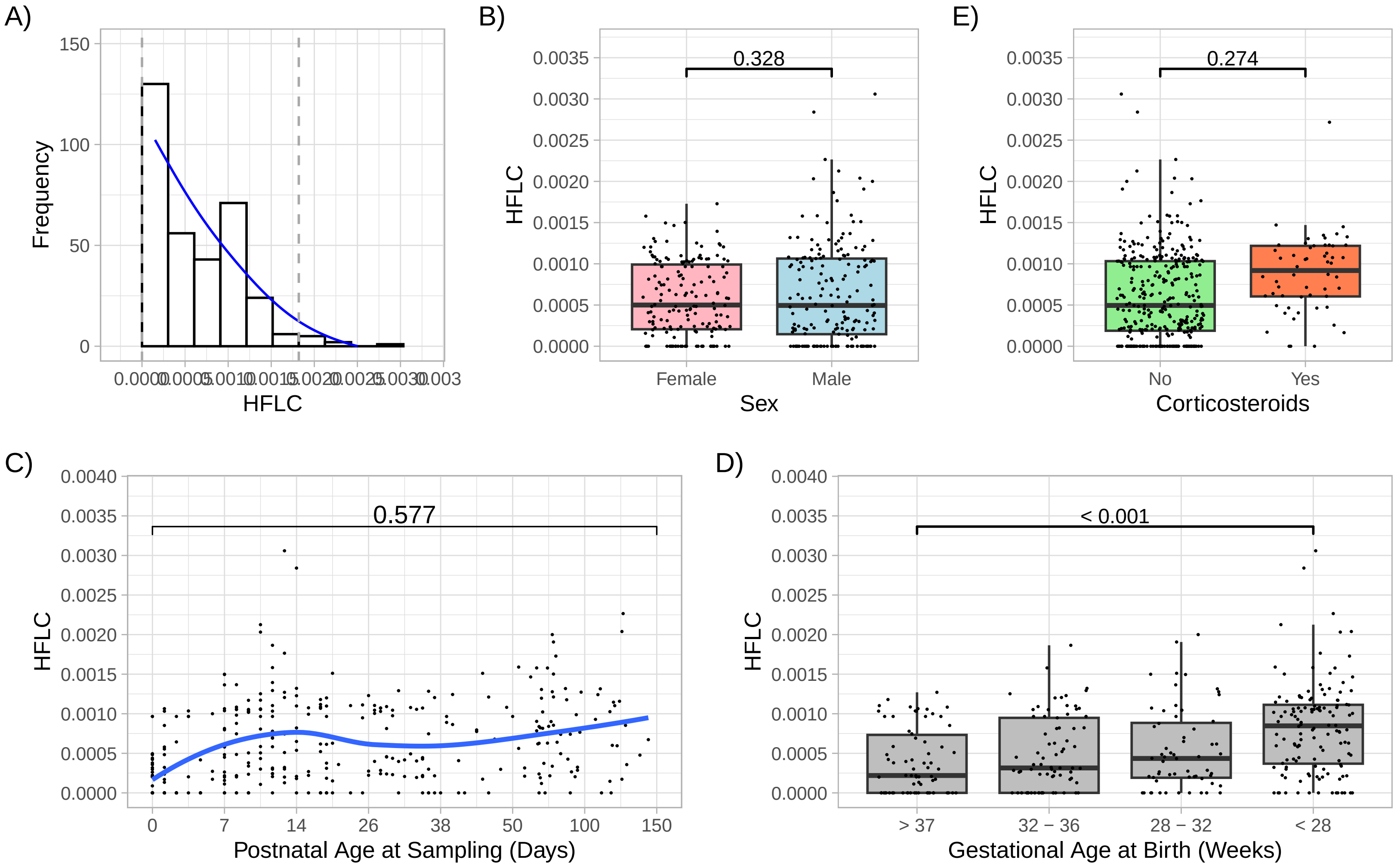

Figure S40: Evaluation of the baseline profile for high fluorescing lymphocyte count (%L) in hospitalised but clinically well neonates. (A) Histogram with reference ranges (grey dotted lines) and a distribution curve (blue line). (B) Box plot of sex. (C) Scatter plot of postnatal age at sampling with a fitted curve (blue line). (D) Box plot of gestational age at birth. (E) Box plot of corticosteroid exposure. Corticosteroid exposed values were removed in plots A to D.

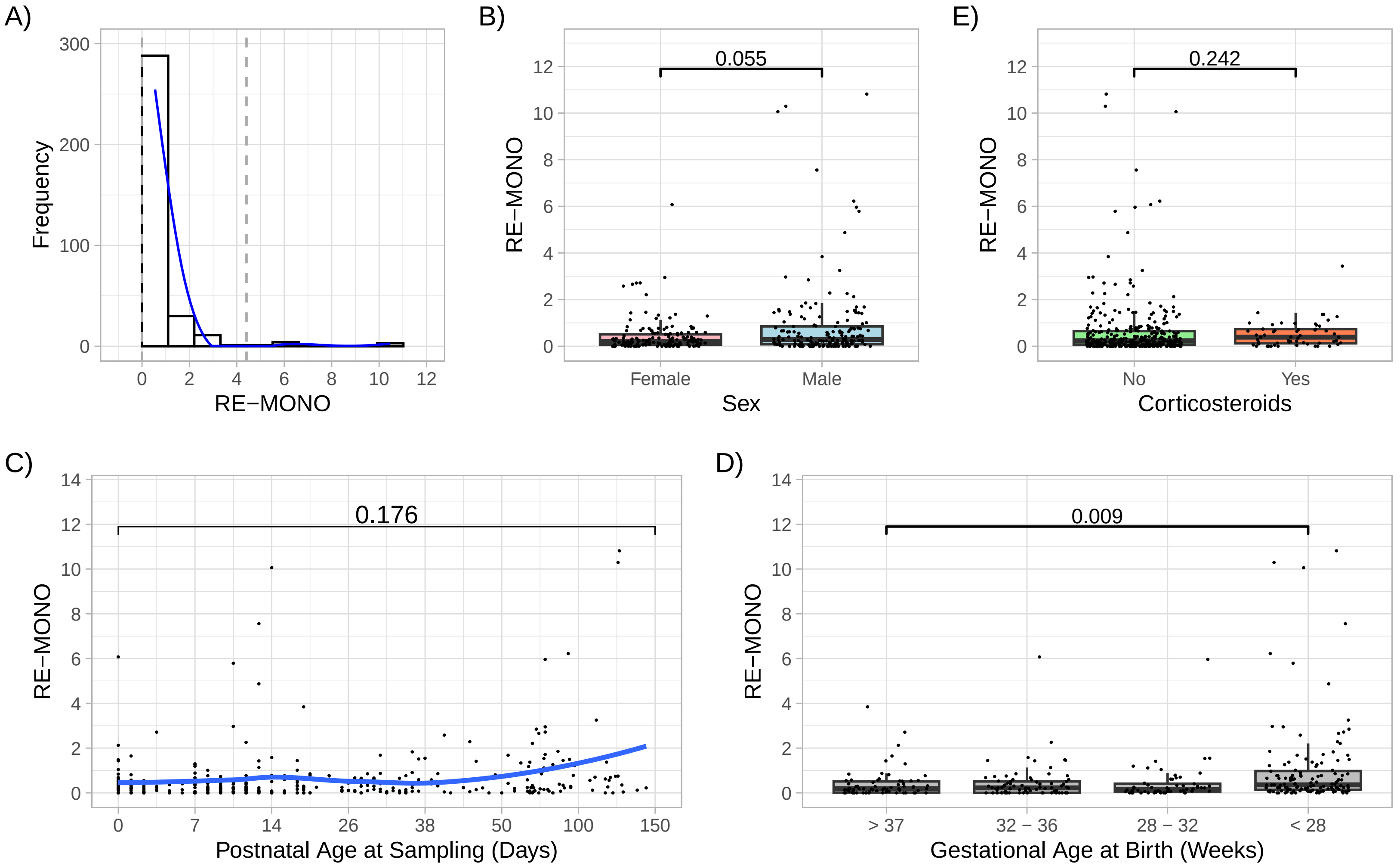

Figure S41: Evaluation of the baseline profile for reactive monocyte count (%M) in hospitalised but clinically well neonates. (A) Histogram with reference ranges (grey dotted lines) and a distribution curve (blue line). (B) Box plot of sex. (C) Scatter plot of postnatal age at sampling with a fitted curve (blue line). (D) Box plot of gestational age at birth. (E) Box plot of corticosteroid exposure. Corticosteroid exposed values were removed in plots A to D.

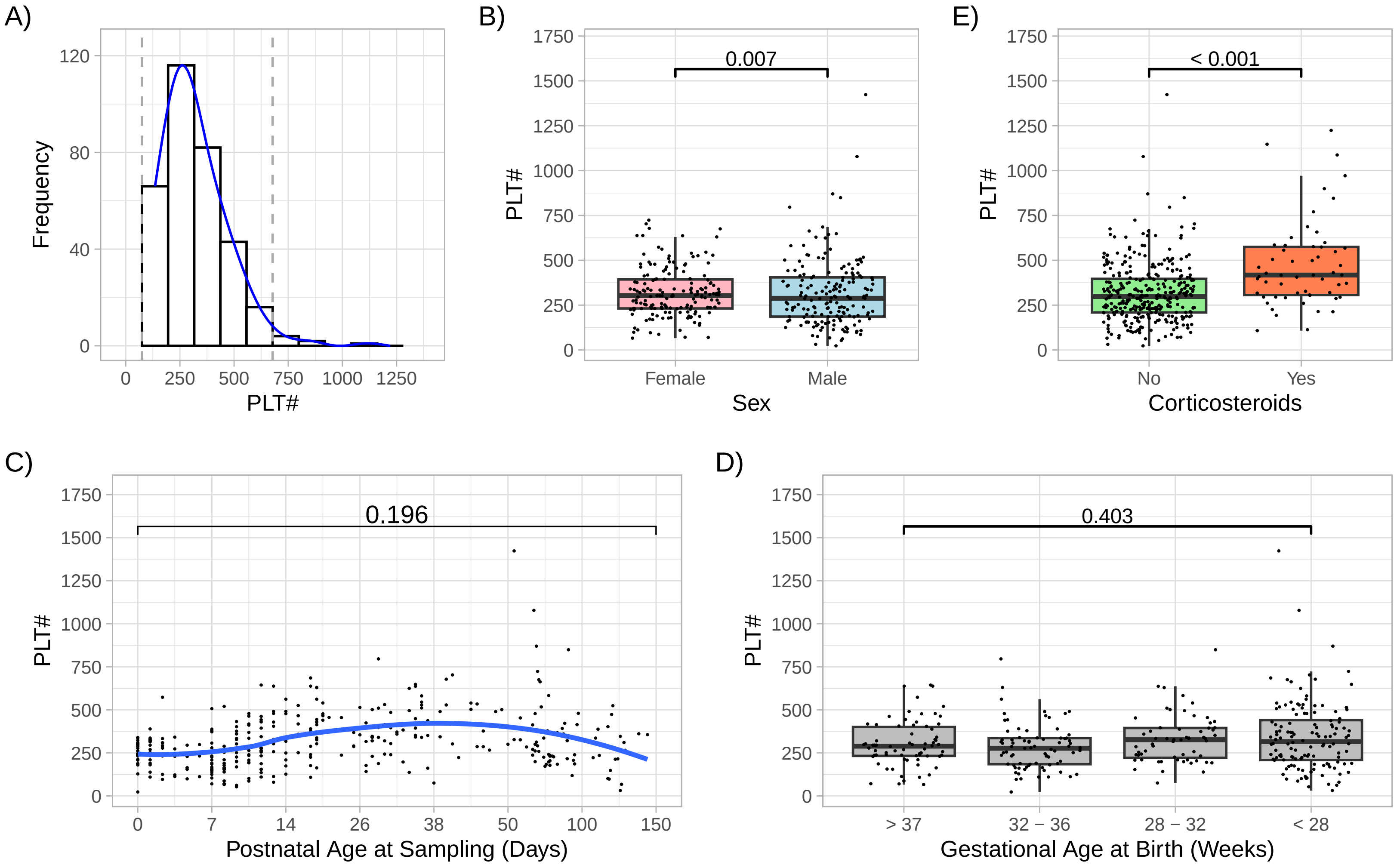

Figure S42: Evaluation of the baseline profile for platelet count (x10^9/L) in hospitalised but clinically well neonates. (A) Histogram with reference ranges (grey dotted lines) and a distribution curve (blue line). (B) Box plot of sex. (C) Scatter plot of postnatal age at sampling with a fitted curve (blue line). (D) Box plot of gestational age at birth. (E) Box plot of corticosteroid exposure. Corticosteroid exposed values were removed in plots A to D.

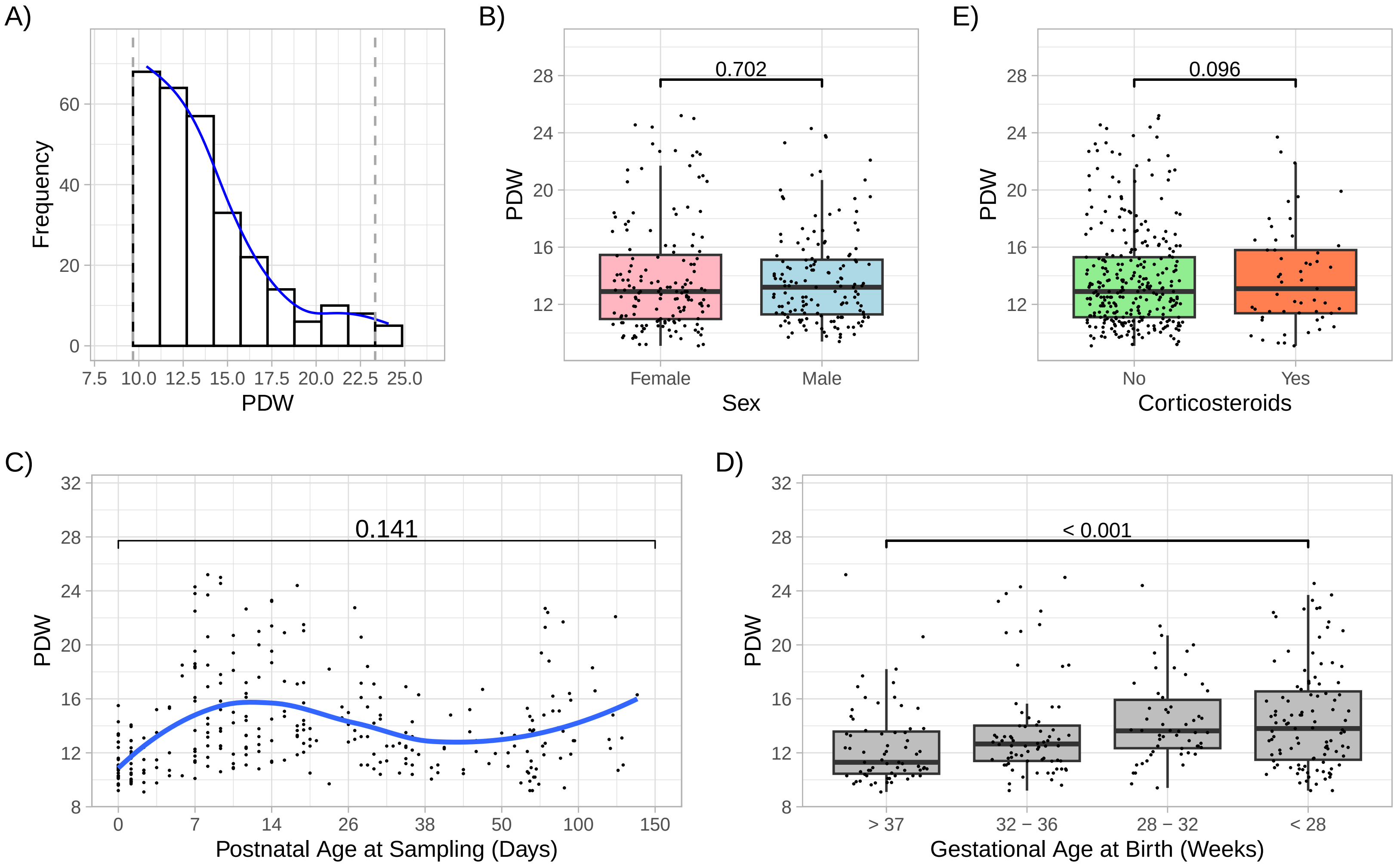

Figure S43: Evaluation of the baseline profile for platelet distribution width (%) in hospitalised but clinically well neonates. (A) Histogram with reference ranges (grey dotted lines) and a distribution curve (blue line). (B) Box plot of sex. (C) Scatter plot of postnatal age at sampling with a fitted curve (blue line). (D) Box plot of gestational age at birth. (E) Box plot of corticosteroid exposure. Corticosteroid exposed values were removed in plots A to D.

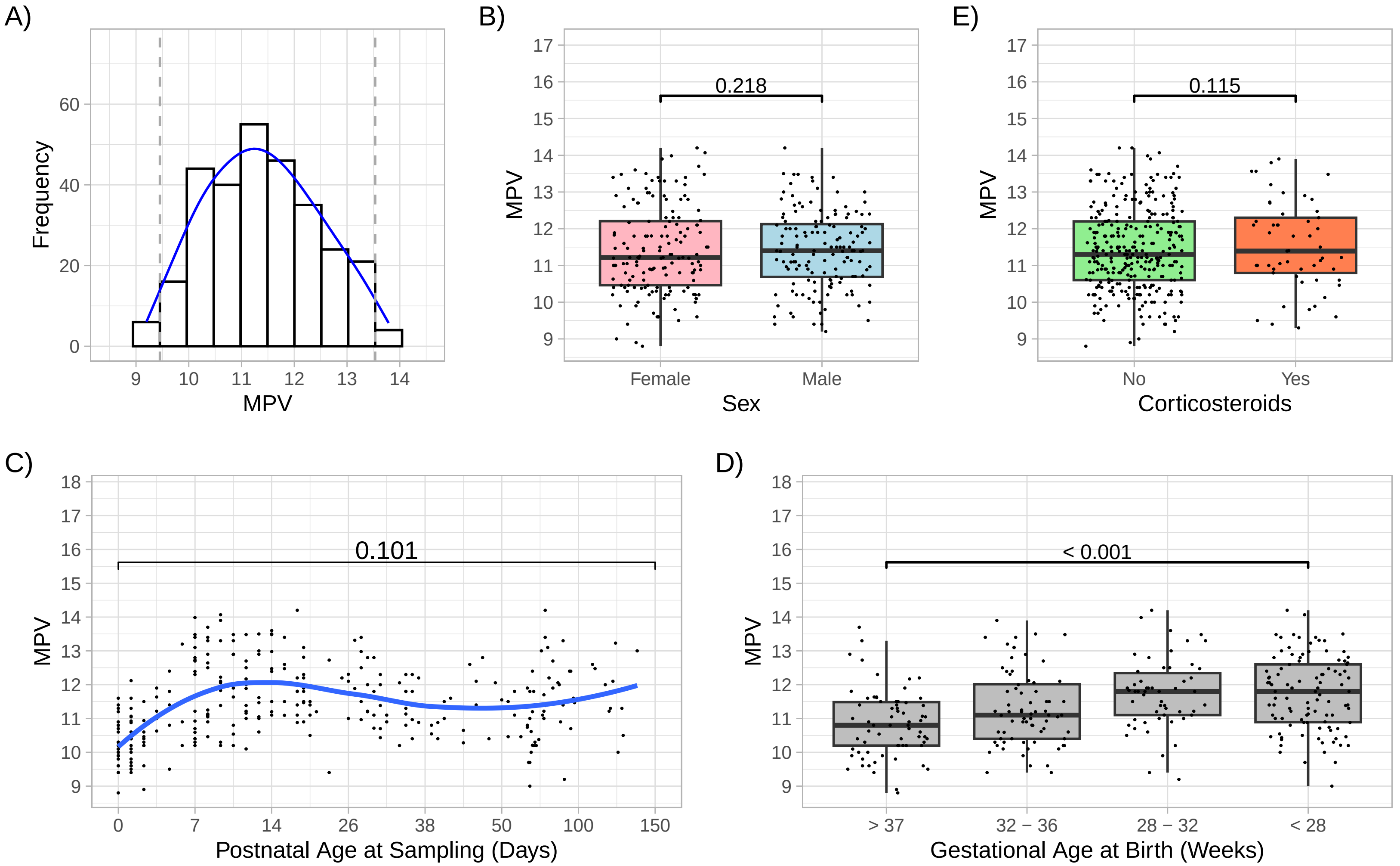

Figure S44: Evaluation of the baseline profile for mean platelet volume (fL) in hospitalised but clinically well neonates. (A) Histogram with reference ranges (grey dotted lines) and a distribution curve (blue line). (B) Box plot of sex. (C) Scatter plot of postnatal age at sampling with a fitted curve (blue line). (D) Box plot of gestational age at birth. (E) Box plot of corticosteroid exposure. Corticosteroid exposed values were removed in plots A to D.

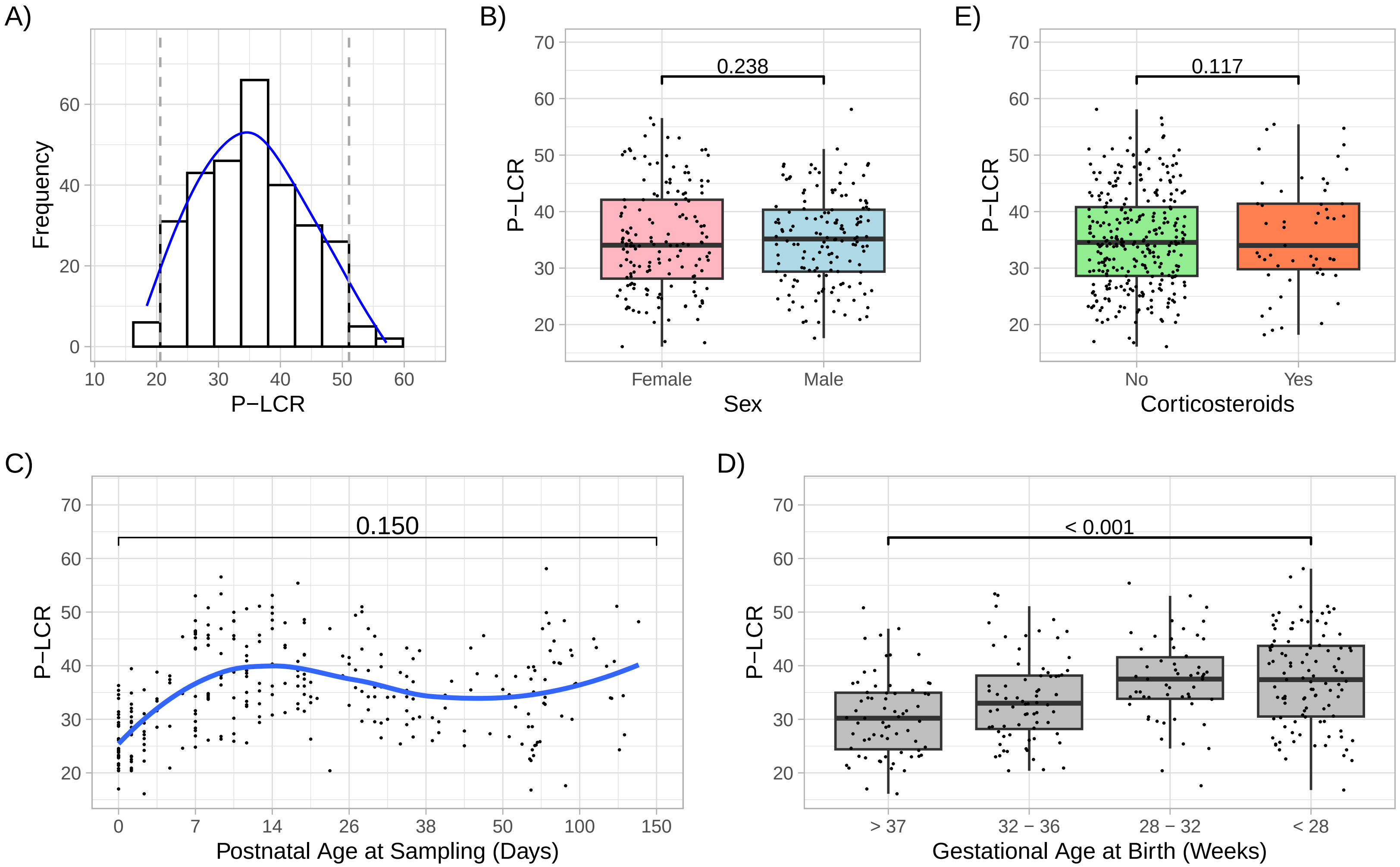

Figure S45: Evaluation of the baseline profile for platelet large cell ratio (%) in hospitalised but clinically well neonates. (A) Histogram with reference ranges (grey dotted lines) and a distribution curve (blue line). (B) Box plot of sex. (C) Scatter plot of postnatal age at sampling with a fitted curve (blue line). (D) Box plot of gestational age at birth. (E) Box plot of corticosteroid exposure. Corticosteroid exposed values were removed in plots A to D.

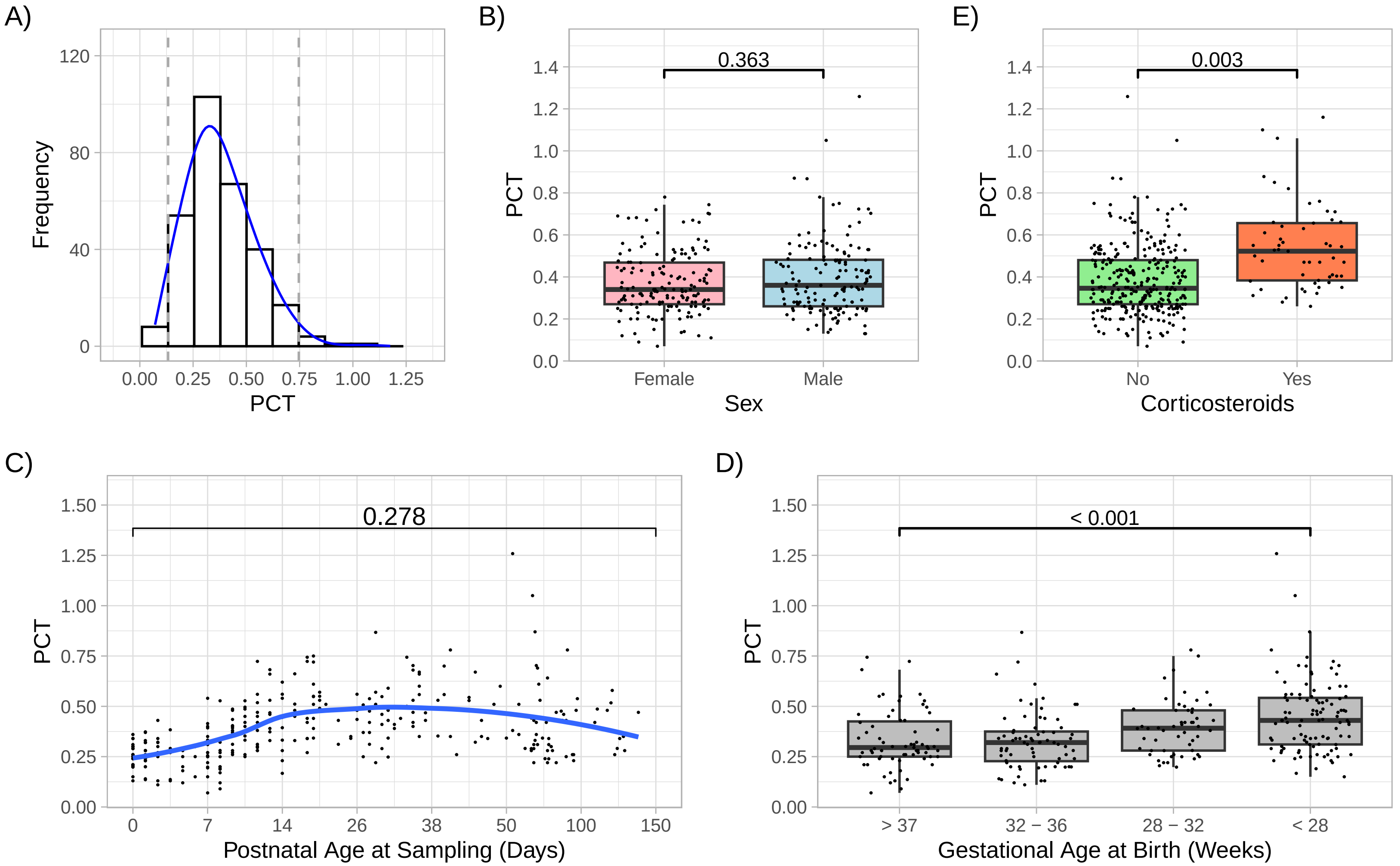

Figure S46: Evaluation of the baseline profile for plateletcrit (%) in hospitalised but clinically well neonates. (A) Histogram with reference ranges (grey dotted lines) and a distribution curve (blue line). (B) Box plot of sex. (C) Scatter plot of postnatal age at sampling with a fitted curve (blue line). (D) Box plot of gestational age at birth. (E) Box plot of corticosteroid exposure. Corticosteroid exposed values were removed in plots A to D.

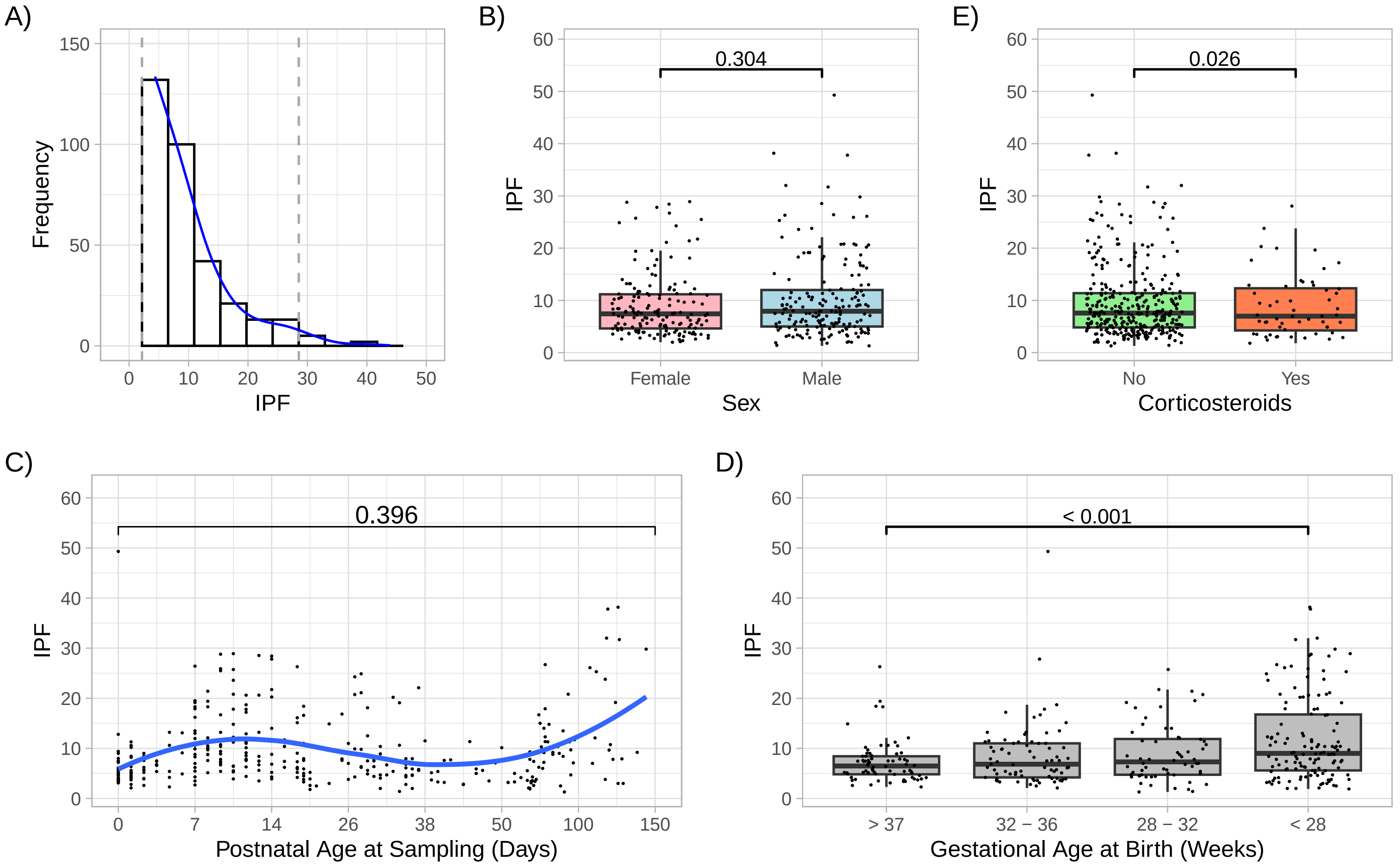

Figure S47: Evaluation of the baseline profile for immature platelet fraction (%) in hospitalised but clinically well neonates. (A) Histogram with reference ranges (grey dotted lines) and a distribution curve (blue line). (B) Box plot of sex. (C) Scatter plot of postnatal age at sampling with a fitted curve (blue line). (D) Box plot of gestational age at birth. (E) Box plot of corticosteroid exposure. Corticosteroid exposed values were removed in plots A to D.

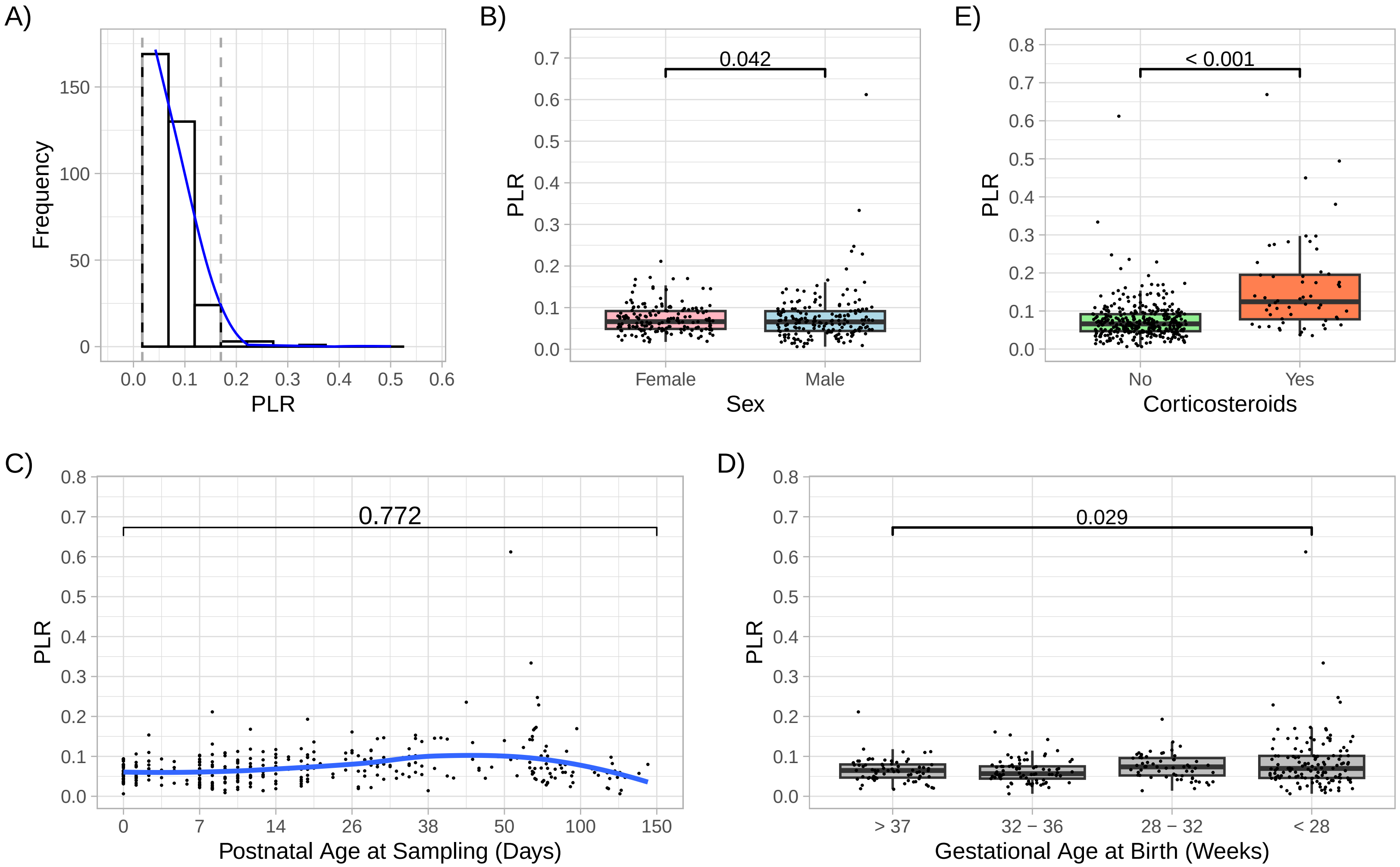

Figure S48: Evaluation of the baseline profile for platelet to lymphocyte ratio (%) in hospitalised but clinically well neonates. (A) Histogram with reference ranges (grey dotted lines) and a distribution curve (blue line). (B) Box plot of sex. (C) Scatter plot of postnatal age at sampling with a fitted curve (blue line). (D) Box plot of gestational age at birth. (E) Box plot of corticosteroid exposure. Corticosteroid exposed values were removed in plots A to D.
